# Fecal Microbiota Transplantation Exerts Neuroprotective Effects in a Mouse Spinal Cord Injury Model by Modulating the Microenvironment at the Lesion Site

**DOI:** 10.1128/spectrum.00177-22

**Published:** 2022-04-25

**Authors:** Yingli Jing, Fan Bai, Limiao Wang, Degang Yang, Yitong Yan, Qiuying Wang, Yanbing Zhu, Yan Yu, Zhiguo Chen

**Affiliations:** a China Rehabilitation Science Institute, Feng tai District, Beijing, People's Republic of China; b China Rehabilitation Research Center, Feng tai District, Beijing, People's Republic of China; c Beijing Key Laboratory of Neural Injury and Rehabilitation, Feng tai District, Beijing, People's Republic of China; d School of Rehabilitation Medicine, Capital Medical University, Feng tai District, Beijing, People's Republic of China; e Center of Neural Injury and Repair, Beijing Institute for Brain Disorders, Feng tai District, Beijing, People's Republic of China; f Experimental and Translational Research Center, Beijing Friendship Hospital, Capital Medical University, Xicheng District, Beijing, People's Republic of China; g Cell Therapy Center, Beijing Institute of Geriatrics, Xuanwu Hospital Capital Medical University, Xicheng District, Beijing, People's Republic of China; h National Clinical Research Center for Geriatric Diseases, and Key Laboratory of Neurodegenerative Diseases, Ministry of Education, Xicheng District, Beijing, People's Republic of China; Nanchang University

**Keywords:** fecal microbiota transplantation, microenvironment, spinal cord injury, vascular repair, inflammation, β-alanine

## Abstract

The primary traumatic event that causes spinal cord injury (SCI) is followed by a progressive secondary injury featured by vascular disruption and ischemia, inflammatory responses and the release of cytotoxic debris, which collectively add to the hostile microenvironment of the lesioned cord and inhibit tissue regeneration and functional recovery. In a previous study, we reported that fecal microbiota transplantation (FMT) promotes functional recovery in a contusion SCI mouse model; yet whether and how FMT treatment may impact the microenvironment at the injury site are not well known. In the current study, we examined individual niche components and investigated the effects of FMT on microcirculation, inflammation and trophic factor secretion in the spinal cord of SCI mice. FMT treatment significantly improved spinal cord tissue sparing, vascular perfusion and pericyte coverage and blood-spinal cord-barrier (BSCB) integrity, suppressed the activation of microglia and astrocytes, and enhanced the secretion of neurotrophic factors. Suppression of inflammation and upregulation of trophic factors, jointly, may rebalance the niche homeostasis at the injury site and render it favorable for reparative and regenerative processes, eventually leading to functional recovery. Furthermore, microbiota metabolic profiling revealed that amino acids including β-alanine constituted a major part of the differentially detected metabolites between the groups. Supplementation of β-alanine in SCI mice reduced BSCB permeability and increased the number of surviving neurons, suggesting that β-alanine may be one of the mediators of FMT that participates in the modulation and rebalancing of the microenvironment at the injured spinal cord.

**IMPORTANCE** FMT treatment shows a profound impact on the microenvironment that involves microcirculation, blood-spinal cord-barrier, activation of immune cells, and secretion of neurotrophic factors. Analysis of metabolic profiles reveals around 22 differentially detected metabolites between the groups, and β-alanine was further chosen for functional validation experiments. Supplementation of SCI mice with β-alanine significantly improves neuronal survival, and the integrity of blood-spinal cord-barrier at the lesion site, suggesting that β-alanine might be one of the mediators following FMT that has contributed to the recovery.

## INTRODUCTION

Spinal cord injury (SCI) is a devastating health issue that often results in complete and permanent loss of motor, sensory and autonomic functions (1). Among the estimated 27 million SCI cases worldwide, about 90% result from trauma (2, 3). During the process of primary injury, blood vessels at the injury site are often damaged, which leads to local hypoxia within and around the lesioned cord as well as the infiltration of inflammatory cells – an important part of the secondary injury mechanism. Following primary injury, a multiphasic degenerative and inflammatory response takes place, and is coupled with severely restricted vascular and neuronal repair, leading to permanent functional deficits (4, 5).

Currently, treatment options for SCI are limited due to the restricted capacity to regenerate the damaged tissue and neuronal circuits. Recently, a growing body of evidence suggests that intervention with intestinal microbiota modulation may offer a potential therapeutic strategy for SCI (6–9). The human intestinal microbiota is composed of 10^13^ to 10^14^ microorganisms whose collective genome (“microbiome”) contains comparable genetic content to their host (10, 11). The intestinal microbiota participates in many physiological processes of the host, including nutrient absorption, metabolism, development and maturation of the immune system and resistance to foreign pathogens (12–15). The intestinal microbiota not only communicates with the central nervous system (CNS) directly through neuronal synapses, but also communicates indirectly with the CNS by secreting metabolites that can cross the blood-brain barrier (6, 16–18).

Following SCI, disruption of the intestinal microbiota exacerbates the pathological changes of the spinal cord, and gut microbiota remodeling is not only beneficial for the repair of intestinal function, but also improves neurological functions (6, 7). Fecal microbiota transplantation (FMT) is the process of transferring functional microbiota from healthy human feces into the gastrointestinal tract of patients, which might impact disease progression by reconstructing the intestinal microbiota (19). FMT and gut microbiota remodeling have shown effects in multiple CNS diseases such as Parkinson's disease, Alzheimer’s disease, stroke, autism spectrum disorders and multiple sclerosis (MS) (20–25). Germ-free mice receiving FMT from PD patients display increased motor dysfunction, whereas FMT from healthy mice shows a neuroprotective effect by inhibiting neuroinflammation and TLR/TNF-α signaling in PD mice (20). In APP/PS1 transgenic mice, FMT alleviates Alzheimer’s disease-like pathogenesis by improving cognitive functions and synaptic plasticity and reducing neuroinflammation (22). In rat middle cerebral artery occlusion models, FMT intervention significantly ameliorates neurological damage, eliminates brain edema, reduces infarct volume, and decreases blood lipid levels (24). Overall, these studies suggest that FMT may impact the deleterious pathological processes activated after the onset of CNS diseases.

Our pervious study showed that FMT facilitates functional recovery and promotes neuronal axonal regeneration following SCI (9). However, the underlying mechanism remains unclear. Numerous studies have shown that alteration of the niche homeostasis is mainly mediated by secondary injury, consequently affecting functional recovery (26, 27). In this study, we aimed to investigate the impact of FMT on secondary injury and functional recovery at the tissue, cellular and molecular levels in SCI mice. We hypothesized that FMT treatment may play a neuroprotective role in neurological function sparing/recovery by improving the niche homeostasis after SCI.

## RESULTS

### FMT treatment significantly improves neuronal survival and white matter sparing in the spinal cord.

**FIG 1:**
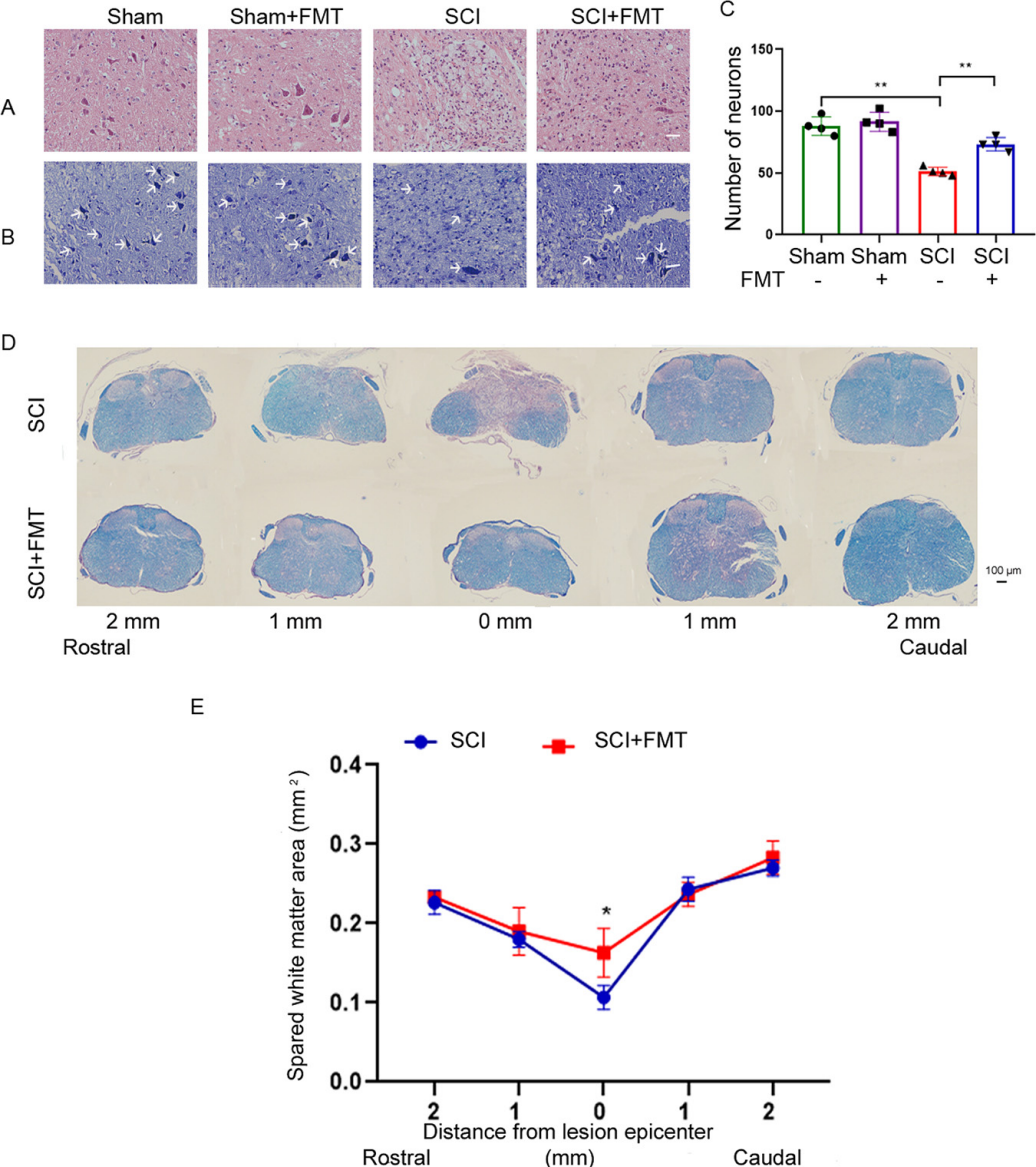
FMT treatment ameliorates pathological features after SCI. (A) Representative images of spinal cord transverse sections after H&E staining. Scale bar = 50 μm. (B) Representative images of spinal cord transverse sections after Nissl staining. Scale bar = 50 μm. (C) The numbers of neurons in the ventral horn of the spinal cord were determined. (D) Representative images of sections spanning from 2 mm rostral to the lesion epicenter to 2 mm caudal after Luxol Fast Blue (LFB) staining. (E) Quantification of spared white matter by LFB staining (*n* = 4); *, *P* < 0.05 compared to the SCI group; **, *P* < 0.01 compared to the SCI group.

In a previous study, we showed that FMT improves locomotor functions and alleviates pathology in the injured spinal cord (9). In the current study, we first aimed to confirm the effect of FMT by examining the pathology of the spinal cord in different groups. H&E staining (Fig. 1A) showed that neurons in the ventral horn exhibited normal morphology in sham controls, with larger cell bodies and intact axons. 4 weeks following SCI, most neurons at the lesion site showed shrunken cell bodies and degenerated axons, accompanied by extensive infiltration of inflammatory cells. Compared with the SCI group, significantly elevated amounts of surviving neurons were observed at the injury site in FMT-treated mice. Consistent with H&E staining, Nissl staining also showed that ventral horn neurons were significantly spared after FMT treatment (Fig. 1B and C). By using Luxol fast blue staining, we investigated white matter damage. FMT administration was associated with a higher degree of white matter preservation, as revealed by white matter volume, in comparison with the SCI control group (Fig. 1D and E). The results confirmed that FMT treatment can improve spinal cord tissue preservation and alleviate pathological features following SCI.

### FMT reduces spinal cord ischemia in SCI mice.

**FIG 2:**
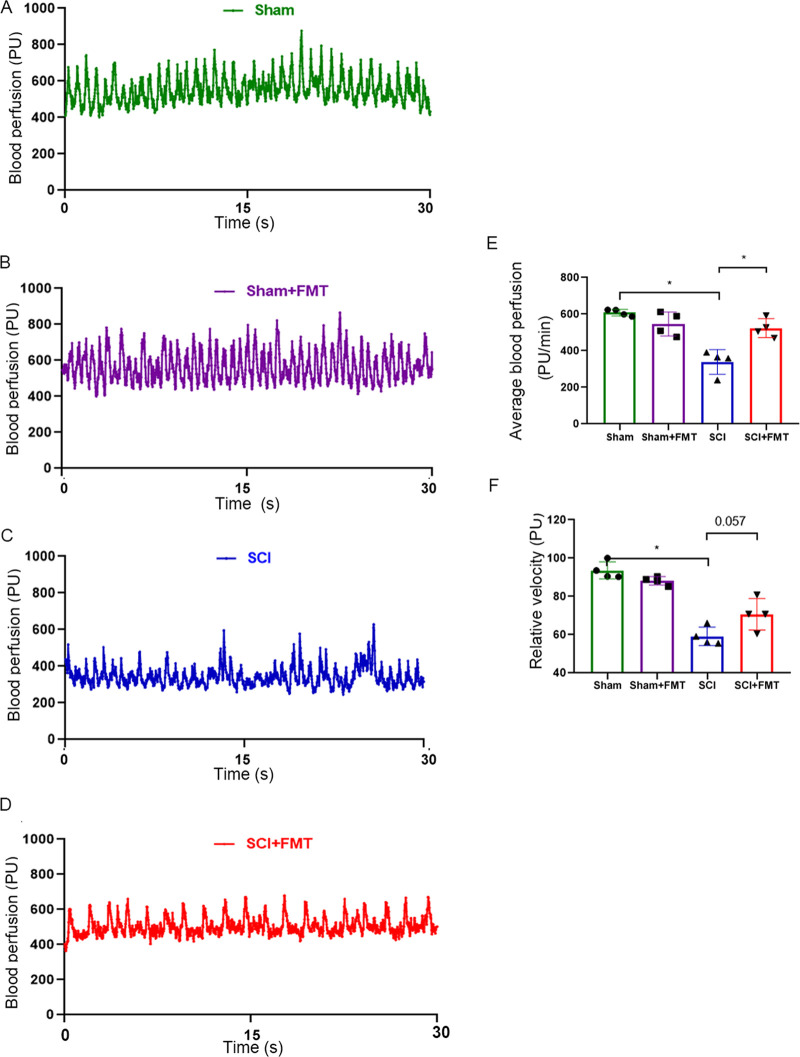
FMT treatment restores blood perfusion following SCI. (A to D) 30-s samplings of raw blood flow outputs from the combined probe among different groups. Functional parameters of hemodynamics, including (E) average blood perfusion and (F) relative velocity, were examined by Laser Doppler perfusion monitoring (*n* = 4). *, *P* < 0.05 compared to the SCI group; **, *P* < 0.01 compared to the SCI group (NS: no significant).

Spinal cord ischemia represents an important secondary damage following contusion injury, which can lead to a series of pathophysiological changes. We applied the Laser Doppler technology to monitor blood perfusion at 4 weeks following SCI in different groups. Figure 2A to D showed the time-domain Laser Doppler signals in which periodicity variation was observed over time in different groups.

Compared with sham controls, average blood perfusion in SCI mice was markedly reduced, which was significantly reversed by FMT treatment (Fig. 2E). The relative velocity was also significantly decreased in SCI mice compared with sham controls, and FMT treatment increased the relative velocity, although the difference was not statistically significant (*P* = 0.057, Fig. 2F). These results suggested that FMT administration improved blood perfusion following SCI.

### FMT treatment improves vascular repair and pericyte coverage.

**FIG 3:**
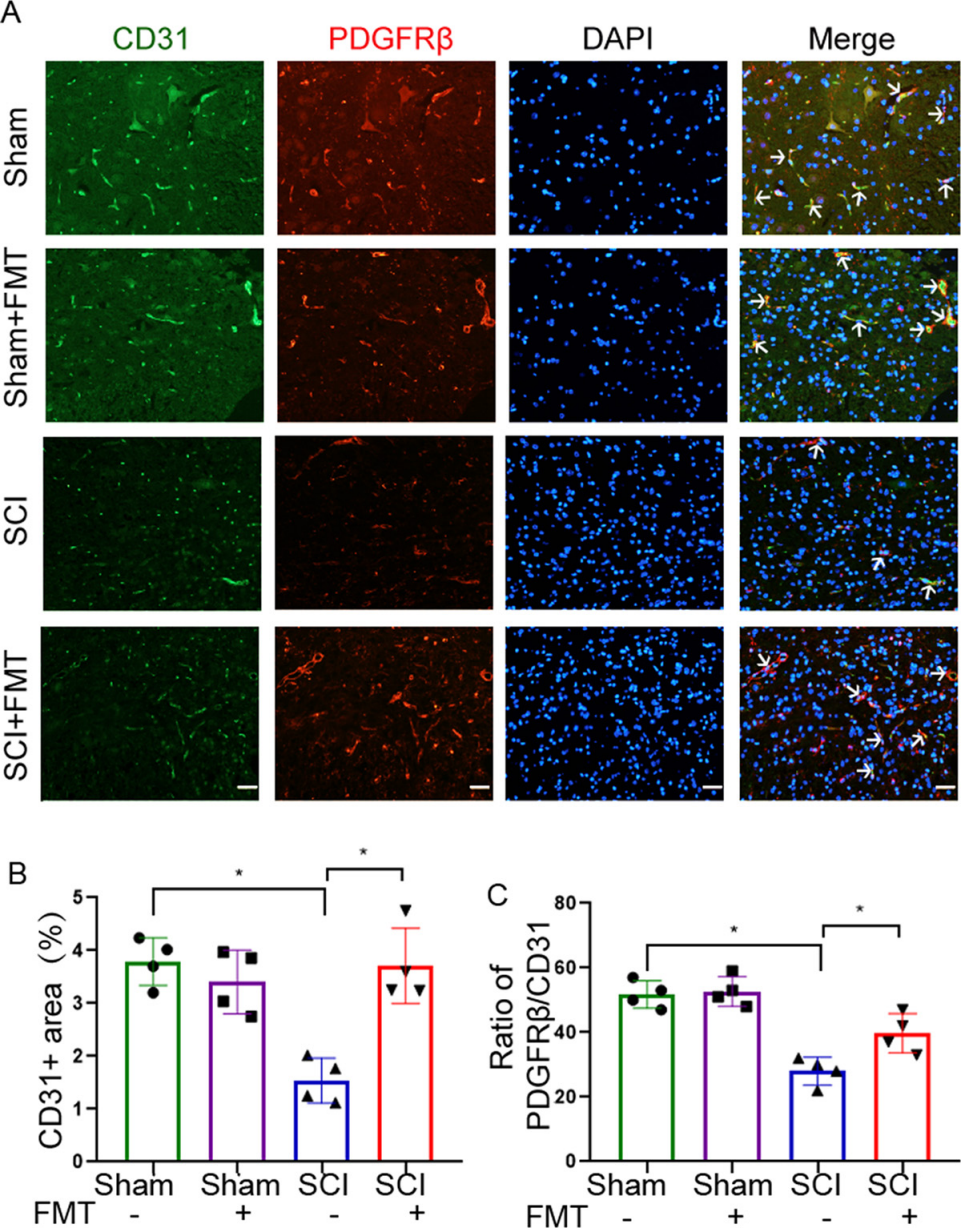
FMT treatment alleviates blood vessel damage and increases pericyte coverage of microvessels after SCI. (A) Representative images showing double immunofluorescent staining for CD31 and PDGFRβ at the lesion epicenter. Scale bar = 50 μm. (B) The proportion of CD31-stained area was quantified 4 weeks post-injury on transverse sections. (C) Pericyte coverage was assessed as the ratio of PDGFRβ+ area to CD31^+^ area (*n* = 4). *, *P* < 0.05 compared to the SCI group; **, *P* < 0.01 compared to the SCI group.

Improvement in blood perfusion is often accompanied by vascular repair. We further determined the effect of the FMT on vascular repair after SCI. By immunofluorescent staining, we examined the expression of CD31, an endothelial marker, at the injury site. Compared with the sham group, SCI mice were characterized by significantly reduced fraction of the area covered by blood vessels at the ventral horn of the spinal cord, which was reversed by FMT treatment (Fig. 3A and B). Pericytes are another important component of the neurovascular unit required for vascular integrity and stability (28). We tested the co-localization of endothelial (CD31) and pericyte (PDGFRβ) markers by immunostaining and found that pericyte coverage in the ventral horn of the spinal cord was larger in the FMT group compared with the SCI group (Fig. 3A and C), suggesting a more stable vascular network after FMT treatment.

### FMT treatment protects against blood-spinal cord-barrier (BSCB) disruption.

**FIG 4:**
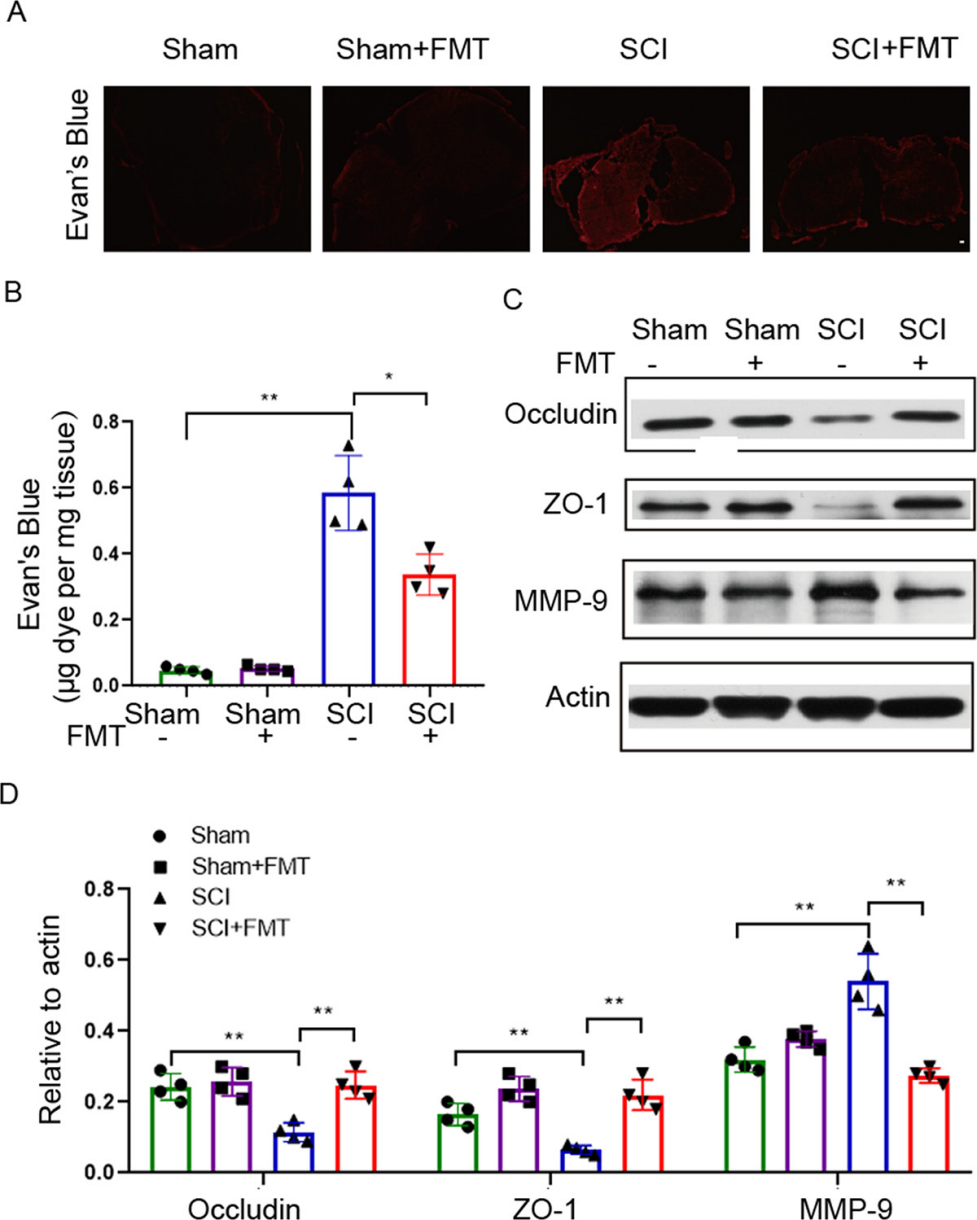
FMT treatment improves BSCB integrity 4 weeks after SCI. (A) Representative fluorescent images of Evan’s blue extravasation at the spinal parenchyma. Scale bar = 50 μm. (B) Quantification of extravasated Evan’s blue (*n* = 4). *, *P* < 0.05 versus the SCI group; **, *P* < 0.01 versus the SCI group. (C to D) Expression levels of occludin, ZO-1 and MMP-9 assessed by Western blotting; relative amounts were quantified (*n* = 4).

Pericyte coverage is not only involved in the stabilization of vessel architecture, but also plays an important role in the maintenance of BSCB integrity (28–30). Next, we examined BSCB permeability by the Evan’s Blue assay. As shown in Fig. 4A, SCI caused a marked increase in Evan’s Blue dye extravasation compared with the sham group, and the amount of extravasated dye was significantly reduced by FMT treatment. The observation on the tissue sections (Fig. 4A) was consistent with the quantitative measurement of dye concentration in the spinal cord tissue (Fig. 4B). Tight junction (TJ) proteins are critical structural proteins for maintaining BSCB integrity (31). To determine the effect of FMT on BSCB integrity, the amounts of the tight junction (TJ) proteins occludin and ZO-1 were assessed by Western blotting. Occludin and ZO-1 were significantly downregulated following SCI, and FMT treatment markedly reversed this effect (Fig. 4C and D). It was reported that matrix metalloproteinase 9 (MMP-9) degrades VE-cadherin, occludin, claudin‐5 and ZO‐1, leading to higher blood‐brain barrier (BBB) permeability (32). We therefore investigated the expression levels of MMP-9 by Western blotting, which showed that FMT treatment reversed MMP-9 upregulation following SCI (Fig. 4C and D). These results suggested that FMT could exert a protective effect on BSCB integrity, possibly through inhibition of MMP-9 and the preservation of TJ structure.

### FMT alleviates gliosis and neuroinflammation in SCI mice.

**FIG 5:**
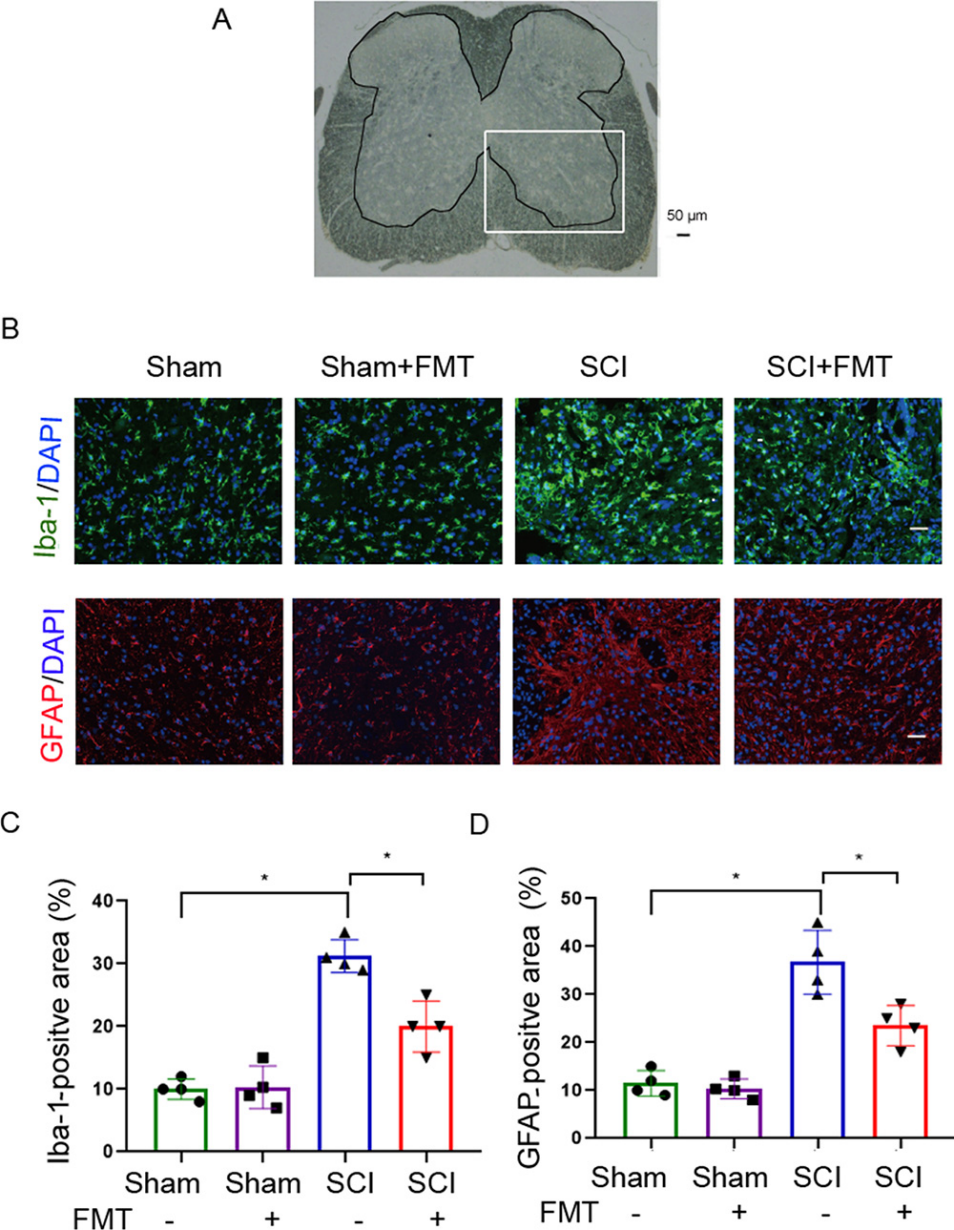
FMT treatment alleviates the activation of astrocytes and microglia in the spinal cord after injury. (A) A spinal cord section with outlined gray matter area and a squared inset of ventral horn. (B) Representative images of immunofluorescent staining for Iba-1 (a marker of microglia) and GFAP (a marker of astrocytes) detection in the ventral horn of the injured spinal cord on day 28 (Scale bar = 50 μm). (C) Quantification of the Iba-1-positive area in the ventral horn of spinal cord on day 28 post-SCI (*n* = 4). (D) Quantification of the GFAP-positive area in the ventral horn of spinal cord on day 28 post-SCI (*n* = 4). *, *P* < 0.05 compared to the SCI group; **, *P* < 0.01 compared to the SCI group.

Next, we examined the levels of gliosis and neuroinflammation in different groups. Microglia and astrocytes are resident glial cells in the CNS that are activated following injury. Iba-1 is a marker of both resting and activated microglia, and GFAP for astrocytes. Immunofluorescent staining of Iba-1 and GFAP at 4 weeks showed that microglia and astrocytes were activated in the ventral horn of the injured spinal cord. The level of activation was markedly reduced in FMT-treated SCI animals (Fig. 5). Microglia assumed a morphology of round cell bodies and shortened branches following SCI, and after FMT treatment of SCI mice, the morphology of microglia returned to a less activated state, with smaller cell bodies and elongated branches (Fig. 5B). Similarly, astrocytes presented a less activated morphology after FMT treatment in SCI mice (Fig. 5B). These data suggested that FMT treatment reduced gliosis and neuroinflammation following SCI.

### FMT treatment reverses the downregulation of neurotrophic factors.

**FIG 6:**
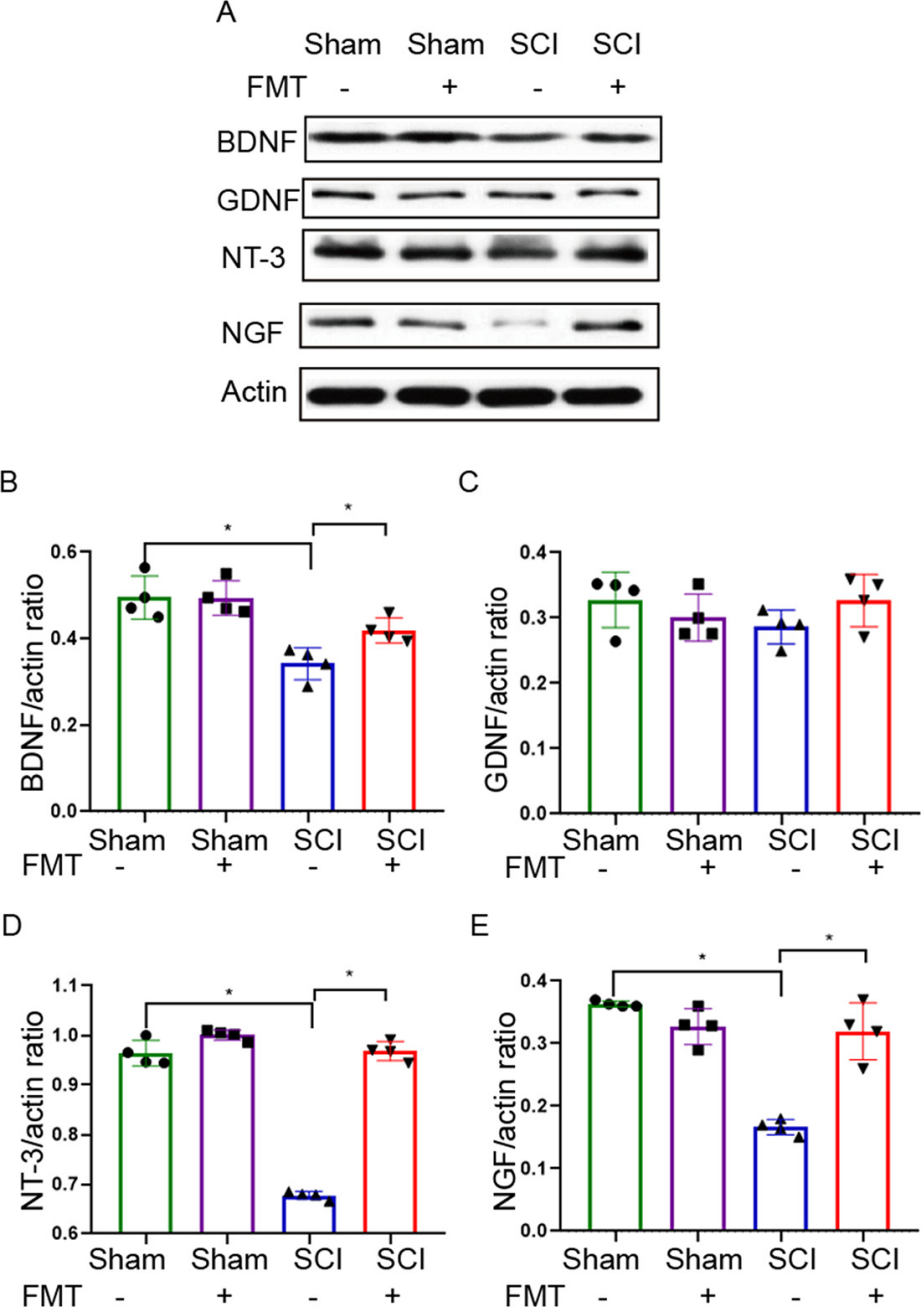
FMT treatment alters the expression of neurotrophic factors in the spinal cord 4 weeks following injury. (A) Expression of BDNF, GDNF, NT-3 and NGF analyzed by Western blotting. The relative amounts of BDNF (B), GDNF (C), NT-3 (D) and NGF (E) were obtained by semi-quantitative analysis in different groups (*n* = 4). *, *P* < 0.05 compared to the SCI group; **, *P* < 0.01 compared to the SCI group.

Beside the pathophysiological changes of the spinal cord, we also examined the alterations at the molecular level by quantitating the expression of neurotrophic factors, including brain-derived neurotrophic factor (BDNF), glial cell-derived neurotrophic factor (GDNF), neurotrophin-3 (NT-3) and neuron growth factor (NGF) in the injured region of the spinal cord. Fig. 6 displays the expression levels of BDNF, GDNF, NT-3 and NGF in the spinal cord on day 28 post-SCI by Western blotting. BDNF, NT-3 and NGF were significantly downregulated in the SCI group versus sham animals, whereas FMT treatment significantly enhanced BDNF, NT-3 and NGF expression, compared with the SCI group (Fig. 6B to E). However, GDNF expression was not statistically different among different groups. These results indicated that the protective effects of FMT might involve the upregulation of neurotrophic and growth factors, especially BDNF, NT-3 and NGF.

### FMT treatment alters the microbiota’s metabolic profile in SCI mice.

**FIG 7:**
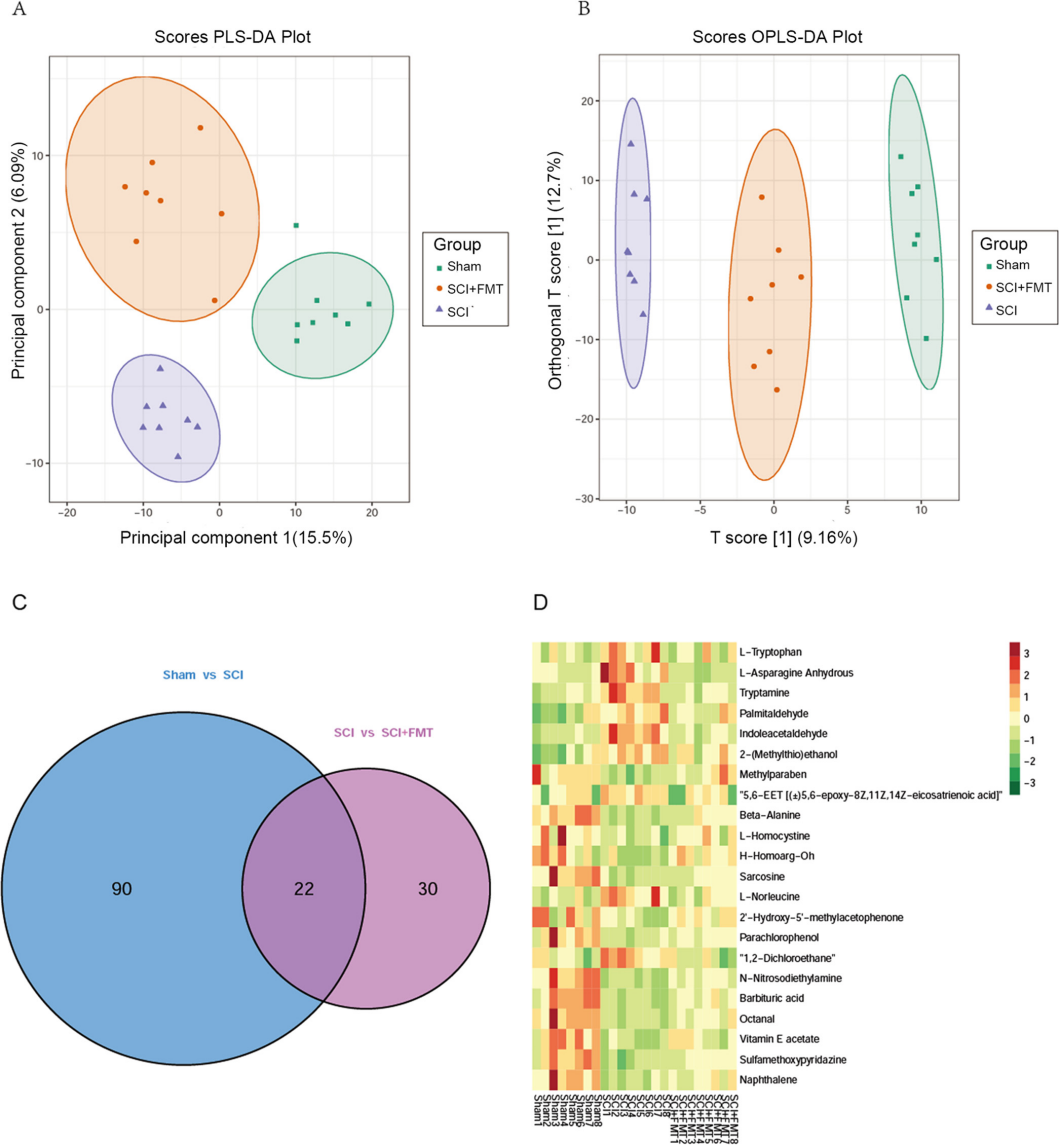
Differential amounts of metabolites among the sham, SCI and SCI+FMT groups. (A) PLS-DA score plot based on metabolic profiles in fecal samples from the sham, SCI and SCI+FMT groups. (B) Score scatterplot of OPLS-DA revealing the separation among groups according to metabolic differences. (C) Metabolites significantly altered in sham animals versus SCI group and SCI group versus SCI+FMT group. Those with VIP >1.0 and *P* < 0.05 (*t* test) were identified. Venn diagrams demonstrating the amounts of altered metabolites shared among the three groups by the overlap (*n* = 8). (D) Relative amounts of 22 differentially produced metabolites in the three groups, presented as a heat map.

The “microbiota-gut-brain axis” refers to the bidirectional communication between the CNS and the gut microbiome. The metabolites produced by gut microbes may exert an indirect impact on the CNS. Therefore, we next investigated whether FMT could alter the microbiota’s metabolic profile in the gut. To distinguish the metabolic profiles among the sham, SCI and SCI+FMT groups, we performed cluster analysis based on partial least-squares discriminant analysis (PLS-DA) and orthogonal partial least-squares discriminant analysis (OPLS-DA). There are all 705 metabolites identified/annotated (Table S2 in the supplemental material). Fecal samples from different groups were separated according to PLS-DA plots (Fig. 7A). Scatterplots revealed that SCI+FMT group versus SCI group clustered closer to the sham group, suggesting an improved recovery from injury after FMT treatment (Fig. 7B). To gain more insights into the metabolic differences between sham and SCI groups, and SCI and SCI+FMT groups, respectively, differential metabolite screening was performed for all 705 metabolites identified/annotated according to *P* values and Variable Importance in Projection (VIP) scores. A *P* value below 0.05 for metabolites with a VIP value above 1 was used as an identification criterion. The screening results were illustrated by Venn diagrams (Fig. 7C). There were 112 differential metabolites detected between the sham and SCI groups, and 52 between the SCI and SCI+FMT groups. A total of 22 differential metabolites were found among the three groups, which are shown in Fig. 7D. There were 10 upregulated metabolites following SCI, including L-Tryptophan, L-Asparagine Anhydrous, Tryptamine, Palmitaldehyde, Indoleacetaldehyde, Indoleacetaldehyde, 2-(Methylthio)ethanol, 5,6-EET [(±)5,6-epoxy-8Z,11Z,14Z-eicosatrienoic acid], H-Homoarg-Oh, L-Norleucine and 1,2-Dichloroethane, which were reduced by FMT administration. There were 12 downregulated metabolites following SCI, including Methylparaben, β-alanine L-Homocystine, Sarcosine, 2′-Hydroxy-5′-methylacetophenone, Parachlorophenol, N-Nitrosodiethylamine, Barbituric acid, Octanal, Vitamin E acetate, Sulfamethoxypyridazine, Sulfamethoxypyridazine, which were increased by FMT administration.

### β-alanine protects against BSCB disruption.

We performed one-way ANOVA and *post hoc* multivariable comparison on the 22 differential metabolites (Table S1 in the supplemental material). According to the statistical results, nine metabolites were upregulated, and 13 metabolites were downregulated following SCI. We first focused on the downregulated metabolites, in that, from a practical point of view, it is easier to supplement the reduced metabolites in food or water than to neutralize the increased ones in a living animal. Among the 13 metabolites, β-alanine was chosen for subsequent validation experiments for its relatively high abundance and the big differences between the groups (mainly SCI Vs. SCI + FMT).

**FIG 8:**
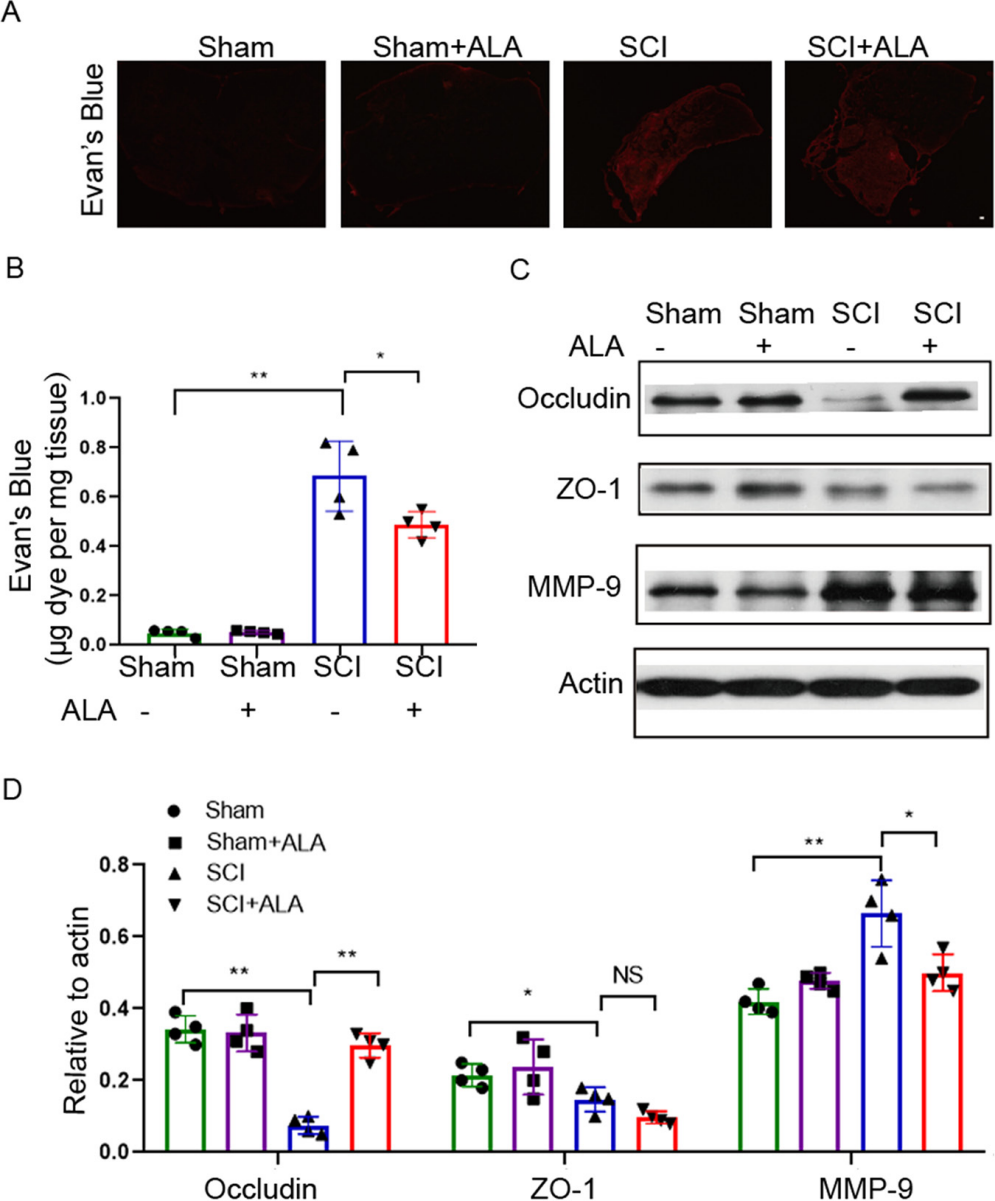
ALA administration enhances BSCB integrity following SCI. (A) Representative fluorescent images showing Evan’s blue extravasation at the spinal parenchyma. Scale bar = 50 μm. (B) Quantification of extravasated Evan’s blue (*n* = 4). *, *P* < 0.05 versus SCI group; **, *P* < 0.01 versus SCI group. (C to D) Expression of occludin, ZO-1 and MMP-9 4 weeks after SCI examined by Western blotting, and semi-quantitative analysis (*n* = 4). ALA, β-alanine.

β-alanine is the precursor of carnosine. It was reported that carnosine may serve as a neuroprotective agent and a powerful inhibitor of MMP-9 (33). However, whether β-alanine supplementation modulates BSCB permeability and MMP-9 production is unknown. The effect of β-alanine on BSCB permeability was examined by the Evan’s Blue assay. As shown in Fig. 8A, β-alanine supplementation significantly reduced the fluorescence intensity of extravasated Evan’s Blue dye in the spinal cord following SCI. In addition, Evan’s Blue dye amounts in the injured spinal cord tissue were higher in the SCI group compared with the sham group but reduced by β-alanine treatment (Fig. 8B). Next, the expression levels of TJ proteins, including ZO-1 and occludin, were examined by Western blotting. The results showed occludin and ZO-1 amounts were reduced after SCI, while β-alanine treatment rescued the expression of occludin but had no obvious effect on ZO-1 (Fig. 8C and D). Further, the expression of MMP-9 was examined by Western blotting. β-alanine treatment reversed the upregulation of MMP-9 following SCI (Fig. 8D). These results suggested that FMT could play a protective role in maintaining BSCB integrity in part through β-alanine.

### β-alanine supplementation promotes weight gain and neuron survival.

**FIG 9:**
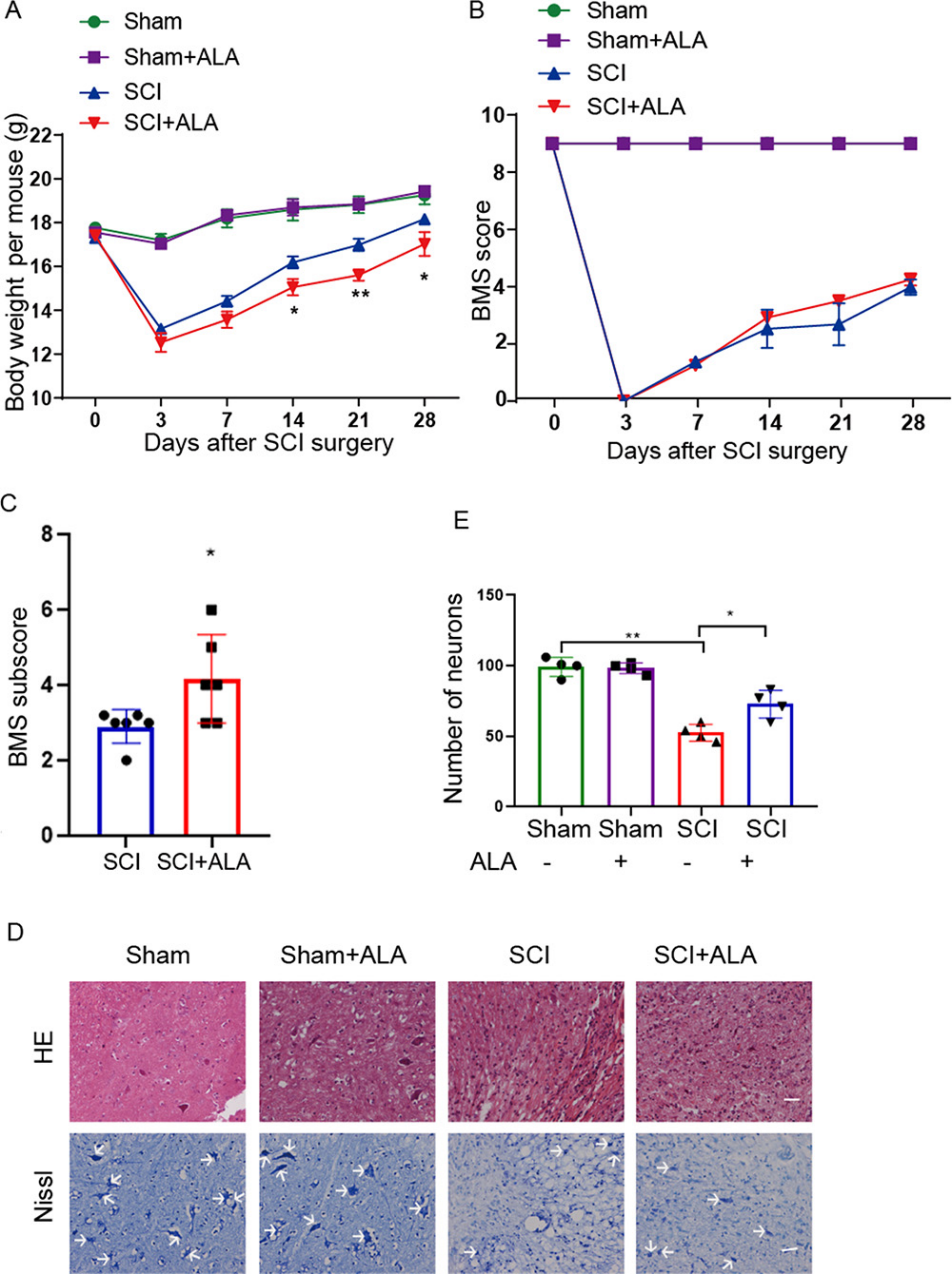
ALA treatment impacts animal behavior and pathological features in SCI mice. (A) Body weight changes during the 4 weeks in different groups. (B) Time course of locomotor function recovery as assessed by the BMS. (C) BMS subscores were assessed on day 28 following SCI (*n* = 8). (D) Representative images of spinal cord transverse sections after H&E staining and Nissl staining. Scale bar = 50 μm (*n* = 4). (E) Amounts of neurons in the ventral horn of the spinal cord. *, *P* < 0.05 compared to the SCI group; **, *P* < 0.01 compared to the SCI group.

Body weights were monitored and compared among groups at indicated time points. Compared with the SCI group, β-alanine treatment led to increased body weight gain, with statistically significant differences at 14-, 21-, and 28-days post-injury (Fig. 9A). The locomotor function was examined during the 4 weeks following injury. BMS scores in the SCI+ALA group were higher than those of the SCI group starting from day 14 after injury until study end, but the differences failed to reach statistical significance (Fig. 9B). However, at peak recovery (4 weeks dpi), the locomotor subscore related to body coordination function, was significantly higher in the SCI+ALA group compared with the SCI group (Fig. 9C). Spinal cord pathology was analyzed by H&E staining 4 weeks after injury (Fig. 9D). Compared with the SCI group, more neurons in FMT-treated SCI mice showed an intact morphology. The amounts of surviving neurons at the ventral horn were quantified on Nissl-stained sections (Fig. 9D). Compared with the SCI group, more neurons were observed in β-alanine-treated SCI animals (Fig. 9E). These results suggested that β-alanine might be a potential mediator of FMT-induced beneficial effects.

## DISCUSSION

In the present study, FMT administration not only enhanced vascular repair, but also suppressed inflammatory responses following SCI. Moreover, FMT promoted the production and secretion of neurotrophic factors and ameliorated the pathological changes of the spinal cord. Improvement in the microenvironment, partly by inflammation suppression and enhanced vascular repair at the injury site following SCI, may generate a permissive niche for a protective and reparative process, which might have accounted for the observed improvement in tissue sparing and functional recovery following SCI.

Vasculature damage/loss after SCI induces a severe ischemic condition within and adjacent to the injury site (34, 35), which may contribute to permanent functional deficits in SCI patients. The present study showed that FMT treatment restored vascular perfusion and stabilized vascular structure with increased pericyte coverage 4 weeks following SCI. The expression of TJ proteins and BSCB permeability were also partially restored by FMT treatment in SCI mice. Restoration of vasculature coverage and function may be a key mechanism underlying the beneficial effect of FMT in SCI mice, and functional vasculature promotes oxygen and nutrient supply to the injured area, thus reducing the extent of ischemic damage and providing necessary trophic factors to facilitate the repair process. The link between the observed vascular recovery and FMT treatment may involve oxidative stress and inflammatory responses after SCI, as reflected by changes in MMP-9 expression after FMT treatment. MMP-9 is associated with BBB disruption in several diseases, including ischemic stroke, intracerebral hemorrhage and brain edema (36, 37), and activation of MMPs could be provoked by oxidative stress and inflammatory cytokines, leading to tight junction disruption and reduced vasculature integrity (38). FMT treatment suppressed MMP-9 activation after SCI, possibly through modulation of inflammatory responses in SCI mice.

Upon SCI, the mechanical force causes blood vessel damage and the infiltration of immune cells into the lesion site; resident glial cells can also sense the injury and become activated. Immune cell activation and related biochemical cascades turn the local niche into an inflammatory condition; if this condition transitions into chronic and persistent inflammation, the hostile microenvironment may inhibit regenerative and reparative processes that otherwise might have taken place at the injury site (39, 40). In the current study, FMT treatment suppressed microglial/macrophage activation after SCI, and changed the morphology of microglia/macrophages from enlarged, round cell bodies with shortened processes, into smaller cell bodies with enlarged processes. Furthermore, astrocyte activation was obviously inhibited at the ventral horn of the lesioned spinal cord by FMT treatment.

To explore the mechanisms of FMT treatment, we next determined the expression of several neurotrophic and growth factors with known associations with reparative and regenerative processes after SCI (41, 42). Within the region of spared spinal cord we detected higher amounts of BDNF, NT-3 and NGF in the FMT+SCI group compared with the SCI group. It is known that BDNF-dependent synaptic plasticity is suppressed by pro-inflammatory cytokines, especially IL-1β (43, 44). FMT treatment suppressed inflammation and downregulated IL-1β/NF-κB signaling in the spinal cord, which might be related to BDNF upregulation. BDNF plays an important role in shaping synaptic plasticity and predicting locomotor performance after SCI (45); in addition, BDNF upregulation significantly ameliorates dendritic atrophy and synaptic degeneration in lumbar motoneurons (46). NT-3 is important for axonal growth and is involved in the neuronal circuits that control normal movement patterns (47, 48). NGF has neuroprotective effects and influences neural responses to injury on α-motor neurons, Schwann cells and sensory neurons that express NGF receptors (49, 50). Furthermore, NT-3 and NGF have been demonstrated to favor axon regeneration and synapse formation after injury (51). These trophic factors have the potential to generate a permissive environment to facilitate meaningful recovery after SCI. Our results suggested that increased tissue sparing adjacent to the injury site may be associated with improved microenvironment with higher levels of trophic factors. Vascular repair and inflammation suppression co-occurred with increased expression of neurotrophic factors, which, together, may transform the hostile microenvironment after SCI into a new homeostasis that favors tissue sparing and the recovery of neural functions.

Concomitant with an alteration of gut microbial composition, we observed a change in microbiota metabolic profile. A total of 22 differential metabolites were detected among the different groups. Of these differential metabolites, β-alanine was selected for further experiments. β-alanine is involved in the rate-limiting step of carnosine synthesis due to its relatively low concentration (52). β-alanine supplementation can augment muscle carnosine content (53). Numerous studies have demonstrated, both at the tissue and organelle levels, that carnosine exhibits pleiotropic biological activities such as reactive oxygen species and reactive nitrogen species amount reduction (54, 55), antagonizing excitotoxicity (56), and MMP-9 inhibition (33). Carnosine is a strong candidate as a neuroprotective agent for stroke treatment, which has been evaluated in rat models of permanent or transient middle cerebral artery occlusion (57). Carnosine also shows a neuroprotective effect against ischemic stroke in mice (58, 59), by reducing reactive oxygen species levels in the ischemic brain, preserving normal glutathione levels and decreasing matrix metalloproteinase protein levels and activities (58). The current study showed that β-alanine supplementation significantly reduced neuronal loss at the ventral horn and improved the coordination function of hind limbs after SCI induction. β-alanine also decreased BSCB permeability and matrix metalloproteinase protein levels. Taken together, β-alanine seems to be neuroprotective in SCI and may improve the damaged microcirculation at the injury site after SCI.

Not only have we examined the effect of β-alanine on BSCS permeability and neuronal survival at spinal cord, but we also measured the extent of white matter sparing, changes in blood perfusion, the levels of vascular damage, activation of astrocytes and microglia, and expression of neurotrophic factors following β-alanine treatment. The data revealed no statistically significant difference in SCI group versus SCI+ALA group (Fig. S1 in the supplemental material). The results hinted that the effects of FMT intervention might have resulted from multiple contributors, and β-alanine alone may not be sufficient to recapitulate all the phenotypes associated with FMT. It is very possible that, other metabolites, alone or in combination, may also play an important role in SCI, which warrants further research. Unfortunately, we didn’t examine β-alanine concentration, or carnosine content after β-alanine treatment. In our further studies, we will investigate the level of β-alanine in plasma and feces in a targeted manner and explore the underlying mechanisms.

In this study, the animals were treated with a mixture of antibiotics before injury, which had eliminated most of the native microbiome in the gut. Pretreatment with antibiotics aimed to mimic a germ-free environment which may further facilitate colonization after the microbiome transplantation (60). We started antibiotics treatment 2 weeks prior to the injury and stopped treatment immediately after injury in order to minimize the impact on spontaneous recovery. However, it is noteworthy that an antibiotics-treated injury group may differ from a normal injury group in some key respects. For instance, in Kigerl’s study, antibiotic cocktails were added 7 days before SCI until 14 days post-SCI, and antibiotics-induced dysbiosis exacerbated lesion pathology and intraspinal inflammation, impairing locomotor recovery. Although, in our study, antibiotics pretreated animals showed no obvious abnormality in locomotor and other functional tests when compared to previous data, the animals did exhibit weight loss, alteration of stool characteristics and so on, which may indicate a slowed recovery.

Although the use of the antibiotics was not ideal, our research focused on the improvement of microenvironment and provided evidence that metabolites, especially amino acids, may be potential mediators of the neural protective effect of microbiome. This strengthened the idea that there is a bilateral communication via small molecules between the CNS and gut. Moreover, our study demonstrated that β-alanine mediated microbiome-elicited neural reparative effect, which may be an intriguing topic worth further investigation. Using small molecules to manipulate or reshape gut microbiome to influence the CNS via the brain-gut-axis may be a viable strategy for intervention of various CNS disorders.

Our data demonstrated that gut microbiota transplantation provides a practical and effective strategy for microbiome-based therapy in the mouse SCI model. The mechanisms of such therapeutic benefits must be multifaceted or at least involve microenvironment improvement at the lesion site, which is associated with enhanced expression of neurotrophic and growth factors that might promote tissue sparing/regeneration and functional recovery after SCI. Future work using germ-free mice or using normal mice without antibiotics pretreatment may be needed to further consolidate the findings of the current study. Meta-genomics combined with multi-omics analysis may also be a useful tool to profile the microbiome with higher resolution, and to gain more insight into the interactions between microbiome and host.

## MATERIALS AND METHODS

### Experimental animals.

Specific pathogen-free adult female C57BL/6N (20 ± 2 g) mice were obtained from the Center of Experimental Animals, Capital Medical University (Beijing, China). Mice were maintained in an air-conditioned room with a 12:12 light/dark cycle, where the temperature was 22 ± 2°C and relative humidity was 55% ± 10%. Food and water were *ad libitum*. Animal protocols were approved by the Animal Care and Use Committee of Capital Medical University.

### SCI.

Mice were anesthetized with 2% isoflurane in a gas mixture of 30% oxygen and 70% nitrogen and placed in a prone position on a heating pad to maintain a constant body temperature. A laminectomy was performed at the T10 level. Mice received a 70-kilodyne spinal cord contusion impact using the Infinite Horizons Impactor (Precision Systems & Instrumentation, Lexington, KY, USA). Afterwards, the incision opening was sutured. During the surgical procedure and recovery period from anesthesia, mice were placed in a warming chamber until they were completely awake. Following injury, animals were hydrated with 0.5 mL Ringer’s solution (s.c.) for 5 days. Bladders were voided manually at least twice daily for the duration of the study. Surgical interventions and postoperative animal care were performed in accordance with the guidelines and policies for rodent survival surgery provided by the Experimental Animal Committee of Capital Medical University.

### Experimental design.

FMT intervention experiment mice were randomly divided into four groups: (1) Sham group; (2) Sham + FMT group; (3) SCI group; (4) SCI + FMT group. Antibiotics were given to adult mice (6 weeks of age) in all four groups through drinking water containing 0.2 g/L ampicillin, neomycin, and metronidazole, and 0.1 g/L vancomycin daily for 2 weeks prior to surgery. After 2-week antibiotics treatment, a total of 100 μL of the resuspended fecal transplant material or vehicle (0.1 mL saline) was given by oral gavage to FMT mice (Sham+FMT, SCI+FMT) daily over a period of 4 weeks. The number of mice in each group for various experiments is shown in Table S3 in the supplemental material.

### β-alanine (ALA) verification tests.

Mice were randomly divided into four groups: (i) Sham group; (ii) Sham + ALA group; (iii) SCI group; (iv) SCI + ALA group. ALA was given to ALA groups (Sham + ALA and SCI + ALA) through drinking water containing 1.2% β-alanine daily for 4 weeks after surgery. The number of mice in each group for various experiments is shown in Table S4 in the supplemental material.

### Preparation of donor fecal transplant material.

The fecal material was collected and isolated as previously reported (9). Antibiotics-untreated, age-matched healthy female mice were kept in the same housing and environmental conditions, which was used as donors to collect gut microbiota. The donor’s fecal pellets were collected under SPF conditions. Stools from donor mice were pooled and 100 mg was re-suspended in 1 mL of sterile saline. The solution was vigorously mixed for 10 s using a benchtop vortex (Vortex-Genie 2, Scientific Industries, USA; speed 9), followed by centrifugation at 800 g for 3 min. The supernatant was collected and used as transplant material. Within 30 min following SCI, fecal transplant material was given by oral gavage to FMT mice.

### Luxol fast blue staining and Nissl staining.

Paraffin-embedded sections were dewaxed with dimethyl benzene, dehydrated with graded ethanol, and washed with distilled water. Luxol fast blue (LFB) staining and Nissl staining was performed as previously described (61, 62). For LFB staining, serial transverse sections (5 μm of thickness) were incubated in 0.1% LFB in acidified 95% ethanol overnight at 60°C. The slides were then counterstained with 0.05% lithium carbonate and cresyl violet solution. LFB-stained tissue sections located the lesion epicenter, 1 and 2 mm rostral and caudal to the lesion epicenter were analyzed. The spared myelin in white matter was quantified by using Image pro-plus (Media Cybernetics, Silver Spring, USA). Nissl-stained images were scanned at high resolution with HistoFAXS 3.0 (Tissue Gnostics, Vienna, Austria), and the images were viewed using the associated professional viewing software (FAXS viewer). The Nissl-positive neurons at the ventral horn of the spinal cord were counted (“positive” definition: intact cells, nuclei and axons) in a double-blind manner.

### Assessment of spinal cord microvascular perfusion.

Spinal cord microvascular perfusion was assessed by using dual channel Laser Doppler monitor (Moor - VMS - LDF2) instrument (Moor Instrument, Ltd., Axminster, UK) and Moor software for Windows version (Moor VMS - PCV3.1, Moor Instrument) as previously described (63, 64). Briefly, mice were examined after a 10 min acclimatization. After anesthetization with 2% isoflurane, the animals were placed on a stereotactic frame to fix the dura and to minimize movement-induced artifacts due to respiration. The body temperature was maintained at 37 ± 0.5°C by using a heating blanket. The Laser Doppler probe was affixed to a micromanipulator and placed perpendicular to the spinal cord, barely touching the dorsal surface of the dura mater. The Laser Doppler probe was positioned 2 mm rostrally to the lesion point at the level just above the surface of the spinal cord and to the right side of the central vein. Changes in perfusion and velocity on spinal cord microvascular vasomotion were evaluated.

### Western blot analysis.

A 1 cm length of spinal cord (epicenter ± 5 mm) was collected. Total protein was prepared in a lysis buffer (Beyotime, China) by lysing tissue homogenates for 1 h, and then centrifugation at 14,000 g for 8 min at 4°C. The protein content of the supernatant was determined by using a protein assay kit (BCA, Pierce, Rockford, IN, USA). Equal amounts of total protein (50 μg) were separated by using 12% sodium dodecyl sulfate-polyacrylamide gel electrophoresis and transferred to polyvinylidene difluoride membranes. The membranes were blocked with 5% nonfat skim milk in Tris-buffered saline solution with 0.05% Tween 20 (TBST) for 1 h, and then incubated with antibodies against BDNF (1:500, Abcam, ab108319), GDNF (1:500, Abcam, ab18956), NT-3 (1:500, Abcam, ab16640), NGF (1:500, Abcam, ab52918), Iba-1 (1:500, GeneTex,GTX100042), GFAP (1:1,000, Sigma, HPA056030), Occludin (1:500, Abcam,ab216327), ZO-1 (1:500, Abcam, ab216880) at 4°C overnight. After 3 washes with TBST, appropriate horseradish peroxidase-conjugated secondary antibodies were added, and β-actin (1:1,000, Cell Signaling Technology) was used as an internal control. The bands were visualized by using enhanced chemiluminescence, and images were acquired with ChemiDoc MP System (Bio-Rad, Hercules, CA, USA). The relative band intensities were quantified by using Quantity One (Bio-Rad, Hercules, CA, USA).

### Immunohistochemistry.

At 4 w after SCI, animals were anesthetized with pentobarbital sodium (35 mg/kg, i.p.), and then perfused with 0.1 M PBS (pH 7.4, 37°C) followed by 4% (wt/vol) paraformaldehyde in 0.1 M PBS. Frozen sections of the spinal cord tissues were prepared at 20 μm thickness by using a cryostat microtome (Leica CM 3500, Wetzlar, Germany) and mounted on gelatin-coated glass slides. Sections were equilibrated in 0.1 M Tris-buffered saline for 10 min. After blocking with 10% normal goat serum in PBS for 1 h, sections of spinal cord were incubated for 1 h with primary antibodies, including rabbit polyclonal anti-Iba-1 (1:100, GeneTex,GTX100042), rabbit polyclonal anti-GFAP (1:500, Sigma, HPA056030), rabbit monoclonal anti-PDGFRβ (1:100, Abcam, ab32570), mouse monoclonal anti-CD31 (1:100, Abcam, ab64543), rabbit polyclonal anti-ZO-1 (1:100, Abcam, ab216880), or rabbit monoclonal anti-occludin (1:100, Abcam,ab216327). After primary antibody incubation, slides were rinsed in PBS, followed by incubation with secondary antibodies and counterstained with DAPI. The slides were coverslipped with glycerinum-mounting media and examined by using fluorescence microscopy. The total area of Iba-1- and GFAP-labeled positive area in each image was automatically calculated by using IPP 7.0, and each individual Iba-1- and GFAP-labeled positive area was then measured by dividing that area by the total area. The total area of CD31-labeled blood vessels in each image was automatically calculated by using IPP 7.0, and each individual CD31-labeled blood vessels was then measured by dividing that area by the total area. For pericyte coverage analysis, the coverage of PDGFRβ-positive area on vessels was determined by using Image Pro Plus 7.0, and this area was divided by the CD31-labeled vessel surface area. Scar area in each image was automatically calculated by using IPP 7.0, and each individual scar area was then measured by dividing that area by the total area.

### Measurement of BSCB permeability.

Evan’s Blue leakage was assessed as previously described (29). EB dye (2% wt/vol in saline) was injected by an intraperitoneal route. After 3 h, mice were anesthetized and perfused transcardially with saline until no more blue dye flew out of the right atrium. The T10 spinal cord segment was removed and sectioned at 20 μm interval. The fluorescence of Evan’s Blue in spinal tissue was examined by using fluorescence microscope. For quantitative measurement of Evan’s Blue extravasation, the T10 spinal cord segment was removed, dried, and weighed. After being homogenized in a 50% trichloroacetic acid solution for 3 d at room temperature, the samples were centrifuged at 10 000 × *g* for 10 min. The supernatants were collected, and the fluorescence signal was quantified at an excitation wavelength of 620 nm and an emission wavelength of 680 nm. All measurements were performed within the range of detection as established by the standard curve. The dye concentration was calculated as the ratio of absorbance relative to the amount of tissue.

### Extraction and separation of metabolites.

Fecal samples were thawed on ice. 50 mg of each sample was homogenized in 1,000 μL ice-cold methanol/water (70%, vol/vol). Cold steel balls were added to the mixture and homogenized at 30 Hz for 3 min. The mixture was vortexed for 1 min, and then centrifuged at 12,000 rpm at 4°C for 10 min. The collected supernatant was used for LC-MS/MS analysis.

The sample extracts were analyzed by using an LC-ESI-MS/MS system (UPLC, Shim-pack UFLC SHIMADZU CBM A system, https://www.shimadzu.com/; MS, QTRAP 6500+ System, https://sciex.com/). The analytical conditions were as follows, UPLC: column, Waters ACQUITY UPLC HSS T3 C18 (1.8 μm, 2.1 mm * 100 mm); column temperature, 40°C; flow rate, 0.4 mL/min; injection volume, 2 μL; solvent system, water (0.04% acetic acid): acetonitrile (0.04% acetic acid); gradient program, 95:5 V/V at 0 min, 5:95 V/V at 11.0 min, 5:95 V/V at 12.0 min, 95:5 V/V at 12.1 min, 95:5 V/V at 14.0 min.

LIT and triple quadrupole (QQQ) scans were acquired on a triple quadrupole-linear ion trap mass spectrometer (QTRAP), QTRAP 6500+ LC-MS/MS System, equipped with an ESI Turbo Ion-Spray interface, operating in positive and negative ion mode and controlled by Analyst 1.6.3 software (Sciex). The ESI source operation parameters were as follows: source temperature, 500 ∘C; ion spray voltage (IS), 5500 V (positive), -4500 V (negative); ion source gas I (GSI), gas II (GSII), curtain gas (CUR) were set at 55, 60, and 25.0 lb/in^2^, respectively; the collision gas (CAD) was high. Instrument tuning and mass calibration were performed with 10 and 100 μmol/L polypropylene glycol solutions in QQQ and LIT modes, respectively. A specific set of MRM transitions was monitored for each period according to the metabolites eluted within this period.

### Statistical analysis.

Significantly regulated metabolites between groups were determined by a variable influence on projection (VIP) >= 1 and *P* < 0.05. VIP values were extracted from OPLS-DA results, which also contained score plots and per mutation plots. PLS-DA and OPLS-DA were generated using R package Ropls and MetaboAnalyst R. The data was log transformed (log_2_) and mean centered before plotting PLS-DA and OPLS-DA. In order to avoid overfitting, a permutation test (200 permutations) was performed. For heatmap analysis, the data were normalized (unit variance scaling), and the heatmap was generated by using the R software complex heatmap package. Venn diagram was generated using the R software Venn Diagram package.

Results were presented as the mean with the standard error of mean (SEM). The data were analyzed by using SPSS, version 17.0 statistic software package (SPSS Inc., Chicago, IL, USA). Student’s t-tests were used to determine significance between two groups. One-way analysis of variance followed by *post hoc* Tukey’s analysis was performed to compare groups of three or more. Values of *P* less than 0.05 were considered statistically significant.
