# Supplementary material for: Fecal Microbiota Transplantation Exerts Neuroprotective Effects in a Mouse Spinal Cord Injury Model by Modulating the Microenvironment at the Lesion Site
Source: Microbiol Spectr. 2022 Apr 25;10(3):e00177-22. doi: 10.1128/spectrum.00177-22 (PMC9241636; doi:10.1128/spectrum.00177-22)
Supplement: SUPPLEMENTAL FILE 1 — Fig.S1, Table S1, Table S2, Table S3, Table S4. Download spectrum.00177-22-s001.pdf, PDF file, 1.4 MB [file spectrum.00177-22-s001.pdf]

Fig. S1

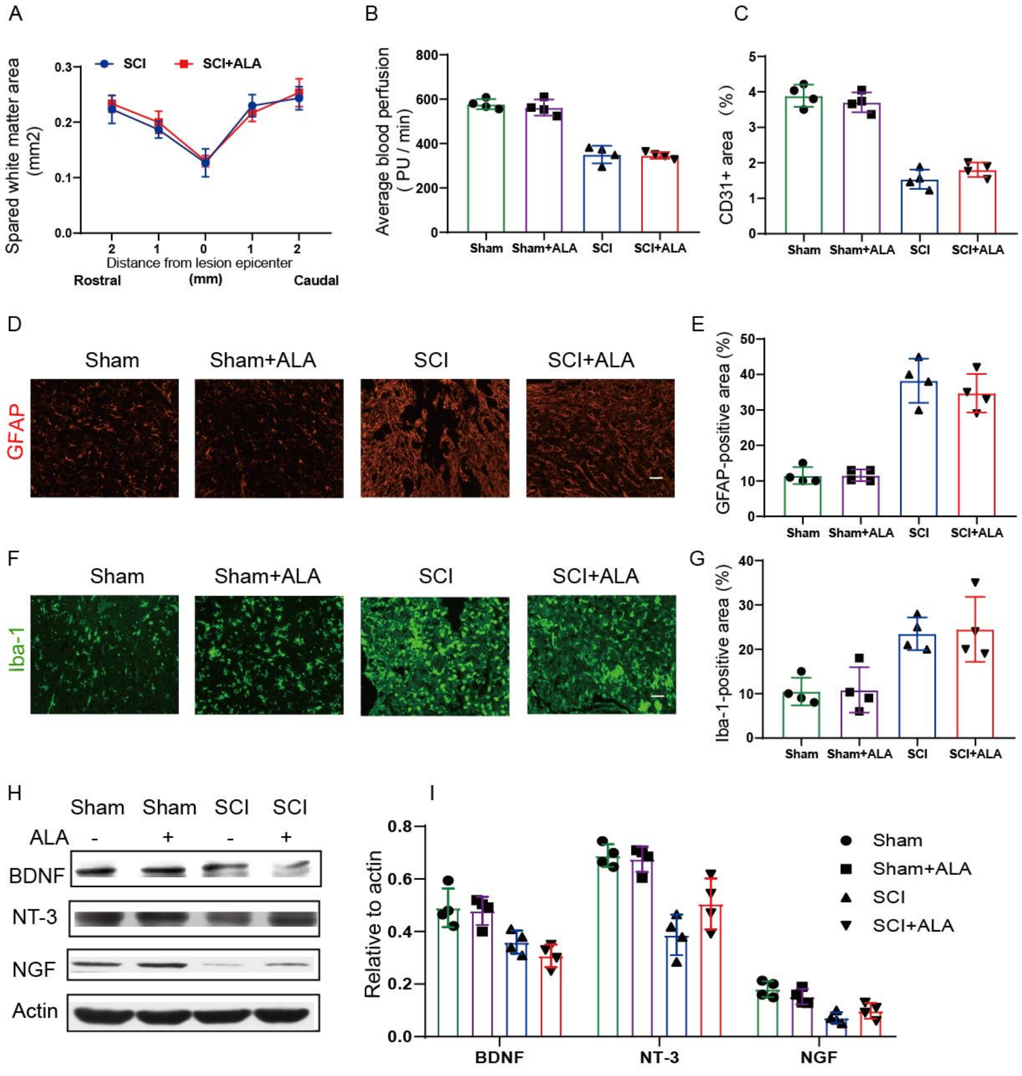

FIG S1

ALA treatment shows no significant impact on white matter sparing, vascular damage, activation of inflammatory cells, or expression of neurotrophic factors. (A) White matter sparing. (B) Average blood perfusion. (C) The proportion of CD31-stained area. (D) Representative images of immunofluorescent staining for GFAP detection. (E) Quantification of the GFAP-positive area. (F) Representative images of immunofluorescent staining for Iba-1 detection. (G) Quantification of the Iba-1-positive area. (H) Expression of BDNF, NT-3 and NGF analyzed by Western blot. (I) The relative amounts of BDNF, NT-3 and NGF by semi-quantitative analysis in different groups.

Table S1. Relative fecal content in different groups.

Metabolites	Relative abundance			P-value	
	Sham	SCI	SCI+FMT	Sham vs. SCI	SCI vs. SCI+FMT
L-Tryptophan	379272.5±39808.77	576532.5±72250.08	381541.25±49060.29	.020	.021
L-Asparagine Anhydrous	48008.875±2253.44	91071.25±11918.38	36736.25±2347.77	.000	.000
Tryptamine	62855.125±5465	110285.75±10095.86	64186.125±4452.22	.000	.000
Palmitaldehyde	2234400±152741.63	3020037.5±102682.86	2675837.5±120957.53	.000	.069
Indoleacetaldehyde	519528.75±36148.69	1070073.75±74924.27	560978.75±36842.97	.000	.000
2-(Methylthio)ethanol	148515±11819.19	205610±6695.66	178937.5±7731.04	.000	.049
Methylparaben	**795801.25±53876.36**	**526127.5±27535.98**	**714402.5±54314.96**	.001	.010
5,6-EET [(±)5,6-epoxy-8Z,11Z,14Z-eicosatrienoic acid]	2053.6±473.37	3540.825±196.24	1795.1±550.17	.025	.010
Beta-Alanine	**2757312.5±108305.65**	**1746475±42970.3**	**2164062.5±78320.37**	.000	.002
L-Homocystine	**684106.25±95255.55**	**412305±43593.37**	**569635±45865.44**	.008	.107
H-Homoarg-Oh	**202585.5±39571.86**	**63031.875±27644.04**	**162401.75±18658.05**	.003	.029
Sarcosine	**4644800±356898.86**	**2849375±114612.84**	**3280700±89839.91**	.000	.185
L-Norleucine	1109170±69210.99	1599512.5±105123.37	1237678.75±67179.8	.000	.005
2'-Hydroxy-5'-methylacetophenone	**163629.25±22425.09**	**95597.25±10384.05**	**146994±8969.3**	.005	.026
Parachlorophenol	**2146250±236696.04**	**1253612.5±56673.47**	**1569962.5±56017.05**	.000	.136
1,2-Dichloroethane	3134937.5±128712.12	3714837.5±141025.18	3036625±131416.88	.006	.002
N-Nitrosodiethylamine	**1663066.25±192619.71**	**629105±56163.83**	**908545±85696.86**	.000	.132
Barbituric acid	**19194125±1399422.59**	**8997550±511284.17**	**12778262.5±835117.8**	.000	.013
Octanal	**1061945±78807.16**	**558101.25±18595.6**	**732085±32300.68**	.000	.023

Vitamin E acetate	**2008000±97848.55**	**1501625±44413.22**	**1744950±76179.97**	.000	.034
Sulfamethoxypyridazine	**2271737.5±107994.49**	**1623775±53837.03**	**1901487.5±36562.34**	.000	.013
Naphthalene	**2272712.5±183777.94**	**1271937.5±48996.18**	**1672662.5±85598.1**	.000	.028

Statistical comparison was performance by using one-way ANOVA and post hoc multivariable comparison. Data were represented as means ± SEM; Sham (n = 8 mice), SCI (n = 8 mice), SCI+FMT (n = 8 mice).

Table S2. The metabolic profiles of microbiota.

Index	Compound	Class	SCI_mean	SCI_FMT	Sham_m	SCI1	SCI2	SCI3	SCI4	SCI5	SCI6	SCI7	SCI8	SCI_FMT	SCI_FMT	SCI_FMT	SCI_FMT4	SCI_FMT5	SCI_FMT6	SCI_FMT7	SCI_FMT8	Sham1	Sham2	Sham3	Sham4	Sham5	Sham6	Sham7	Sham8
MEDN007	L-Arginine	Amino Acid m	1078488	1E+06	1E+06	799410	1943700	858520	719710	1231100	663420	1927600	484440	948660	2E+06	2E+06	534650	1774200	1066100	1084000	2517600	553770	2476100	1112400	1437900	877800	593330	1210900	662960
MEDN009	L-Aspartic Aci	Amino Acid m	4303425	4E+06	4E+06	3E+06	3149300	5847800	3804400	3851300	3943900	4404300	6E+06	3E+06	3E+06	4E+06	5110600	3660500	2150700	5627000	5214000	6687200	2516700	6654600	2826300	3595400	3E+06	3549800	4002800
MEDN010	L-Citrulline	Amino Acid m	270678.8	559114	1E+06	322090	232710	157920	373580	316760	203420	222470	336480	360980	521890	445660	345250	759980	1312200	543380	183570	3178700	1691500	993380	2468000	1491400	630100	324760	332490
MEDN011	L-Glutamic Ac	Amino Acid m	2.05E+08	2E+08	2E+08	2E+08	2.34E+08	189690000	1.8E+08	2E+08	2.2E+08	2.4E+08	2E+08	2E+08	2E+08	2E+08	2E+08	2.3E+08	184980000	1.85E+08	229450000	2.21E+08	211420000	211480000	2.16E+08	222230000	2E+08	210150000	2.08E+08
MEDN015	L-Phenylalanii	Amino Acid m	2715088	3E+06	2E+06	2E+06	2475600	3313700	3198900	2831200	1772500	1903400	4E+06	3E+06	2E+06	3E+06	2683400	2342700	4046300	7057200	1770300	1629700	1330000	1870300	1431100	3102900	3E+06	1998600	4131500
MEDN017	L-Pyroglutami	Amino Acid m	1007425	1E+06	951169	1E+06	1189200	921410	946300	945380	1067200	844310	1E+06	971910	1E+06	1E+06	901650	1121000	1139300	920810	1127600	1123500	803680	970390	784460	1031700	858450	879970	1157200
MEDN018	L-Serine	Amino Acid m	706515	625408	605880	793420	528110	664120	1078700	647120	451030	591370	898250	601110	475420	655770	695510	501310	714110	742810	617220	625140	376390	414460	438990	691480	777200	516080	1007300
MEDN019	L-Tryptophan	Amino Acid m	576532.5	381541	379273	528250	834200	628790	431690	369780	570300	900740	348510	267400	404750	406840	221200	633350	329820	275210	513760	471650	262990	570710	290200	451610	347140	253600	386280
MEDN022	(5-L-Glutamyl	Amino Acid m	349615	418756	320066	315380	327920	243720	271110	524950	374230	466050	273560	421820	394750	381350	283490	558860	589030	281760	438990	284920	350690	297520	396860	281690	296020	391240	261590
MEDN028	4-Hydroxy-L-	Amino Acid m	67683.88	66100	67637	80481	94495	57577	50521	57424	66769	82897	51307	43749	64198	87268	76631	81445	43115	29815	102580	87044	72519	47128	79496	77905	58000	80300	38701
MEDN030	5-Hydroxy-L-	Amino Acid m	50870.25	55940	48310	44242	55243	51059	47419	58373	48936	60393	41297	45471	54176	62218	41229	52421	71964	72201	47840	27851	47803	56067	51161	54139	50633	46195	52632
MEDN032	Allantoin	Organic Acid A	493063.8	406444	167234	392260	425760	1406000	369400	543730	212560	326320	268480	183430	184820	287280	115920	656490	99022	1336500	388090	75138	144750	121490	129530	370680	198790	112330	185160
MEDN036	D-Alanyl-D-A	Amino Acid m	895421.3	1E+06	1E+06	817960	1103000	705490	678140	1509800	668040	1193800	487140	793430	2E+06	2E+06	689660	1164200	1334100	470040	1717200	914890	2018000	1260200	2387200	901330	683190	1666000	823270
MEDN037	Dopamine	Polyamine	14098.78	13496	15966	13486	9178.6	21747	21652	6956.6	11875	12627	15268	13889	21097	16672	10983	12633	15606	7408.1	9682.6	15913	15657	14248	21139	17034	10150	10131	23456
MEDN041	Hexanoyl Glyc	Amino Acid m	1011488	2E+06	60204	424130	264640	3960300	279970	1536600	80442	397920	1E+06	285020	407940	1E+06	129180	6031300	74239	5323600	245860	128500	29813	90886	37822	78776	33809	25061	56964
MEDN042	L-Asparagine	Amino Acid m	91071.25	36736	48009	148550	109360	92564	112050	61131	47079	59593	98243	33484	41408	47254	29002	28243	41962	34544	37993	51965	57364	52115	42991	43548	42397	40324	53367
MEDN043	L-Carnosine	Amino Acid m	23919.63	28429	26309	23871	36209	18995	18773	33128	17986	21419	20976	26791	29427	41639	18643	32851	34544	12196	31343	14870	13704	23739	38134	35273	18182	43777	22790
MEDN044	L-Cystathionir	Amino Acid m	58386.38	74684	55513	56126	116820	31966	35927	60409	46388	74042	45413	84555	165410	94381	42547	86478	51600	20635	51862	60174	101890	39191	86544	29132	33166	64951	29052
MEDN045	L-Cysteine	Amino Acid m	14470.21	19774	16313	12686	9	14712	17383	12291	8798.7	9	49873	8597.9	2591.2	9	40094	9	25440	81445	9	4796.1	8559	19829	10279	26938	22922	5809	31370
MEDN047	L-Homocitrull	Amino Acid m	202642.5	245574	191959	222000	221720	226650	126770	221640	247820	242880	111660	307530	236310	327070	248510	233680	200380	81332	329780	205320	201260	214220	228990	224700	171150	101110	188920
MEDN049	L-Saccharopir	Amino Acid m	10523388	1E+07	1E+07	1E+07	14320000	7930100	8070000	9868300	1.1E+07	1.4E+07	8E+06	9E+06	1E+07	1E+07	8728500	1.3E+07	6082200	2636200	15828000	12550000	14725000	9231800	14545000	9746600	7E+06	11298000	7552600
MEDN052	N6-Acetyl-L-l	Amino Acid m	581047.5	900496	580289	436440	577390	412870	741040	849750	524330	580290	526270	1E+06	764500	780020	554830	1211400	1055300	1035500	737120	479140	460720	433530	603220	809350	638140	530450	687760
MEDN054	N-Acetylcyste	Amino Acid m	6138.038	4264.3	2973.4	10374	9	9669.5	7175.7	5331.9	4648.2	11887	9	9	21276	9	9	9	9	12784	9	5180.6	6565.8	9	6274.1	9	9	5730.4	9
MEDN056	N-Acetyl-L-Le	Amino Acid m	6232438	6E+06	6E+06	7E+06	8111900	6493500	5260900	4530600	6123100	7954700	4E+06	5E+06	7E+06	7E+06	5213200	5019200	2505300	3486900	10275000	9228700	5535500	5643200	6263000	6024700	5E+06	5196500	5001200
MEDN057	N-Acetyl-L-Ty	Amino Acid m	135489.4	289506	134962	200840	134610	99657	124170	110600	57008	250010	107020	522070	97546	189810	101650	356740	309520	573850	164860	100110	78048	113150	81858	242260	184570	76696	203000
MEDN058	N-Acetylneura	Amino Acid m	5762.588	4727.1	6854.1	4770.3	4782.4	5122.4	8145.7	5321.6	5940.9	6438	5579.4	6003.3	4210.3	5172.7	6205.9	4916.3	5761	5538.2	9	6532	6359.9	6747.9	4785.2	9711.4	5383.4	5741.1	9571.7
MEDN059	N-Glycyl-L-Le	Amino Acid m	60666.5	74136	60843	57882	60235	56598	65276	74940	42549	44285	83567	76294	53337	69091	61564	68956	114460	106630	42757	57237	53820	33084	53498	86692	65707	50148	86558
MEDN060	N-Isovaleroylg	Amino Acid m	801531.3	1E+06	91187	253100	469700	3465200	162840	1151100	120360	300380	489570	980280	1E+06	1E+06	219120	2409100	133040	3093300	233140	152270	61347	102870	69446	118030	74546	70182	80803
MEDN062	N-Propionylgl	Amino Acid m	107324.5	99671	75408	97849	101050	275400	69959	115860	68489	73487	56502	85749	126440	95401	72403	146760	109370	94521	66726	150330	28486	68853	29436	92426	83764	74117	75852
MEDN063	Nα-Acetyl-L-	Amino Acid m	166149.6	392431	67526	9	515130	251600	9	243780	9	205390	113270	709590	865970	338330	199610	468320	144160	281110	132360	36870	140330	98383	46782	9	81069	67378	69389
MEDN065	O-Phospho-L	Amino Acid m	151261.3	174398	181958	169110	170690	129970	146980	179280	151070	151300	111690	165910	231060	228010	132080	175900	188630	147510	126080	125350	197920	151710	164780	143710	206590	264820	200780
MEDN066	Phenylacetyl-l	Amino Acid m	220740	329451	102857	247160	556030	159790	134780	180270	149090	211600	127200	509500	635260	439670	217170	440690	138460	121120	133740	118730	86787	115900	123400	116160	84628	92861	84391
MEDN081	P-Coumaric A	Benzene and su	4887213	4E+06	4E+06	4E+06	3079700	4033800	8576600	4607100	2940500	3349000	8E+06	4E+06	3E+06	4E+06	2971900	2577100	4221600	6658000	1312200	7538100	3367800	1362100	3624600	5840400	5E+06	1585000	5695900
MEDN084	Benzoic Acid	Benzene and su	515020	527818	473544	444140	524150	442150	682260	606820	465220	445400	510020	433600	638070	625240	328620	506460	412790	714490	563270	396450	439940	453440	431290	582510	488310	400020	596390
MEDN085	Benzoylformic	Benzene and su	20938.75	26439	27103	31370	28495	25437	19815	9	29950	16362	16072	30523	37513	21286	20299	35030	16724	18335	31805	62669	12778	25413	15440	31945	26911	21516	20150
MEDN086	Terephthalic A	Benzene and su	1425005	1E+06	1E+06	1E+06	1511500	1822600	1364800	633140	1872100	1702500	1E+06	881830	2E+06	2E+06	950580	1824100	746810	973150	2038600	2283500	891890	1189800	848320	1404200	886580	1147700	1197200
MEDN089	2,5-Dihydroxy	Benzoic Acid A	1553483	846339	881083	837950	803910	1043200	1262900	1793900	3572000	1223300	2E+06	2E+06	513510	472970	1098400	1331100	482550	388030	921450	1029100	993170	653250	1237200	939400	822250	462820	911470
MEDN090	2-Methoxyber	Benzoic Acid A	55253.25	68152	70632	63564	56279	59489	55568	39639	63532	64224	39731	61136	83757	64936	67380	66583	63264	71642	66516	102040	55682	88087	58294	88791	60379	70915	40870
MEDN092	3,4-Dihydroxy	Benzene and su	98912.88	225131	23276	158250	302950	9	64117	40564	86152	96689	42572	392070	779880	158130	124610	183130	37946	125270	9	28876	9	9	52844	23611	9	44662	36191
MEDN093	3-Hydroxyant	Benzoic Acid A	67654.88	82464	92921	51385	99176	84412	45951	51895	67281	113230	27909	37370	109500	95462	55490	114320	79087	91740	76740	85691	85678	284140	79954	72858	36235	63210	35603
MEDN097	P–Hydroxyphe	Benzene and su	377391.3	479950	573251	360360	632800	349890	147650	270810	358610	762110	136900	114350	672170	587160	218590	938330	520100	429870	359030	383110	585640	2022400	542530	432390	158820	227210	233910
MEDN098	2-Picolinic Aci	Pyridine And Py	18850138	2E+07	2E+07	3E+07	25034000	13338000	9468100	1.6E+07	1.9E+07	2.5E+07	2E+07	1E+07	2E+07	2E+07	1.1E+07	1.9E+07	17507000	7887100	19981000	15499000	16242000	12298000	21056000	16236000	1E+07	15894000	12730000
MEDN105	Taurocholic Ac	Bile Acids	1E+08	1E+08	1E+08	9E+07	43915000	100270000	1.1E+08	8.3E+07	3.6E+07	3.7E+07	3E+08	1E+08	6E+07	7E+07	2E+08	5.9E+07	191950000	3.5E+08	25581000	15900000	42457000	268980000	37994000	141450000	9E+07	34801000	1.5E+08
MEDN109	Hyodeoxychol	Bile Acids	11188775	2E+07	1E+07	9E+06	14186000	9393800	9514400	2E+07	7534100	1E+07	1E+07	3E+07	2E+07	1E+07	1.3E+07	1.7E+07	50351000	22431000	8583300	5921700	12385000	30644000	17780000	15882000	1E+07	7732000	4288900
MEDN111	Glycodeoxych	Bile Acids	207399.4	253913	216642	197780	186900	99465	244190	107200	467260	200890	155510	422300	611840	85791	309790	74802	214940	142950	168890	9	252290	318140	639810	148740	193090	146290	34768
MEDN112	Glycocholic Ac	Bile Acids	1749384	3E+06	2E+06	990960	1294800	1657600	1978600	3314700	450170	938240	3E+06	5E+06	3E+06	2E+06	3474600	1233900	3173000	6564000	1289600	1120800	1525900	871740	2462600	7266700	2E+06	1086100	760860
MEDN113	Glycochenode	Bile Acids	203139.4	239977	213044	210500	225520	56465	348040	124620	317800	197220	144950	362250	559980	64335	315450	85970	231160	114810	185860	84890	294670	292890	594830	107040	183140	146880	9
MEDN114	Deoxycholic A	Bile Acids	42864500	6E+07	4E+07	4E+07	41086000	36221000	4.8E+07	6.5E+07	2.7E+07	2.9E+07	6E+07	1E+08	6E+07	4E+07	3E+07	2.2E+07	182820000	54113000	17390000	21325000	37303000	33524000	63899000	46818000	6E+07	26941000	18127000
MEDN115	Chenodeoxycl	Bile Acids	43524250	6E+07	4E+07	4E+07	42337000	36688000	4.9E+07	6.7E+07	2.7E+07	3E+07	6E+07	1E+08	6E+07	4E+07	3.1E+07	2.3E+07	153180000	55910000	16854000	21380000	38446000	32124000	66041000	48020000	5E+07	28351000	18155000
MEDN117	4-Methylcatec	Phenols And Its	9337.688	9155.3	10582	7756.6	9543.1	9856.2	11626	6023.1	11968	8086.6	9841.9	10992	14014	5253.4	4301.9	7237.5	9828.3	8206.4	13409	19989	9445.7	9180.7	5325.1	11772	11866	4283.5	12790
MEDN121	Enterodiol	Phenols And Its	6443.2	167773	174958	9	9	38833	9	9	5141	4828.1	2707.5	9914.6	6974.4	5867.1	3276.1	305850	293350	716940	9	9	9	1399600	9	9	9	9	9
MEDN124	Protocatechui	Phenols And Its	35775.88	202770	19163	66410	77792	9	9	37843	56501	47634	9	82118	132050	89746	35263	659580	556580	9	66811	18211	39606	20094	54527	9	9	20837	9
MEDN128	Vanillin	Phenols And Its	149883	162534	181044	90539	84775	116790	139640	304310	111200	110620	241190	201430	158060	187350	136360	140160	190260	178130	108520	91260	180420	265350	143960	176850	177270	174910	238330
MEDN132	Biotin	Co-Enzyme Fac	261691.3	290708	180468	266120	344890	154830	144460	315110	320410	401970	145740	269470	428940	299090	224730	353320	300870	147930	301310	135230	125980	322450	134700	137290	231980	167180	188930
MEDN139	1-Methylxantl	Nucleotide met	1935425	2E+06	2E+06	2E+06	4272100	1740400	1065100	933010	1458100	2682600	941690	2E+06	4E+06	4E+06	1272600	2538300	1620700	878070	2253300	2150100	2238300	1867000	2624000	1473000	1E+06	2277500	1192700
MEDN140	2,6-Dihydroxy	Nucleotide met	17084888	2E+07	2E+07	2E+07	25725000	20022000	1.6E+07	7770100	1.2E+07	1.9E+07	2E+07	1E+07	2E+07	2E+07	1.5E+07	1.9E+07	16589000	15141000	16676000	22670000	15349000	8095300	15688000	25559000	1E+07	15842000	15149000
MEDN141	2'-Deoxyuridi	Nucleotide met	492430	452463	323110	689850	1251300	204400	259450	258450	289900	827370	158720	242680	738220	822900	191310	577900	699690	142070	204930	417520	403910	83689	258120	553870	266600	256690	344480
MEDN144	3-Methylxantl	Nucleotide met	24771.25	36334	13158	41946	28670	26087	14197	19609	30267	18110	19284	47863	78703	29855	36142	33060	29105	19920	16024	6646.7	8102.1	26798	9	13649	15053	10722	24285
MEDN146	5-Hydroxyme	Nucleotide met	159630.4	123371	80458	137900	131640	483830	131720	92639	86610	82264	130440	131950	58852	113550	57929	115400	101290	295170	112830	65737	61989	114980	47961	118670	110320	55692	68311
MEDN147	5-Methylcytos	Nucleotide met	67751.75	161476	50481	52846	28356	163890	41846	80942	58250	34341	81543	40087	22056	70605	30602	26006	39292	1032400	30756	39195	24790	192910	33303	27616	42530	28858	14643
MEDN153	Adenosine 5'-	Nucleotide met	115667.3	97733	144283	150030	79612	44215	38369	135300	172390	246100	59322	26640	87811	120120	218800	102020	19605	8388.5	198480	136820	130440	673570	70660	44491	19003	51764	27518
MEDN156	Cytidine-5-Mo	Nucleotide met	121013	131956	110256	159980	75596	116790	60443	64619	279940	156370	54366	53955	112160	79986	384690	152650	37695	51452	183060	193650	42942	229780	111950	94983	51181	77697	79862
MEDN164	Inosine	Nucleotide met	85500875	9E+07	3E+07	1E+08	1.19E+08	62330000	5.3E+07	8.6E+07	9.1E+07	1.4E+08	4E+07	8E+07	1E+08	1E+08	6.7E+07	1.1E+08	66955000	28994000	124160000	3890600	20094000	80946000	4888800	19894000	3E+07	78381000	35426000
MEDN165	Inosine 5'-Mo	Nucleotide met	39322.75	36105	31946	27212	43556	31026	18529	48733	43232	78061	24233	22806	42588	24516	70188	29893	18986	11534	68328	35374	45135	73725	26385	17901	9	24836	32204
MEDN166	Nicotinic Acid	Nucleotide met	30069	24832	63077	21729	19815	9	16905	26319	40495	71183	44097	12315	17656	35650	31546	34020	4906.1	5234	57329	28869	24892	350530	16953	14995	16020	16826	35530
MEDN169	Uracil	Nucleotide met	1037781	821501	803590	706620	776060	1597700	1229100	1694000	814100	808580	676090	1E+06	621060	573370	1178800	406300	852010	711060	856610	433890	888270	1029300	1081500	726530	680570	878920	709740
MEDN170	Uridine 5'-Dip	Nucleotide met	23946.63	36900	26326	24179	43976	12111	22980	24223	24479	26087	13538	29729	57136	57322	18869	39936	28446	20272	43491	28014	36520	18665	41960	30208	12979	24841	17422
MEDN171	Uridine	Nucleotide met	3866300	5E+06	2E+06	4E+06	5929100	3220000	2442600	3137200	4316100	5727500	2E+06	4E+06	6E+06	5E+06	3302700	5739500	3573500	3779100	4895300	932730	957430	4382300	737530	1170000	2E+06	3147400	1318300
MEDN198	Citric Acid	Amino Acid m	547127.5	537908	416844	635260	380470	905160	657570	480980	595950	424820	296810	547650	721890	927800	320280	712340	303280	282600	487420	913830	346280	395450	331000	425610	359890	270110	292580
MEDN199	D-Malic Acid	Amino Acid m	1821438	2E+06	2E+06	2E+06	2479900	1288600	1248500	2004300	2009700	2503200	1E+06	2E+06	3E+06	2E+06	1525900	2316200	1490600	1160700	2876300	1643000	1998400	1709100	2046500	1609800	2E+06	1764700	1523000
MEDN200	L-Malic Acid	Amino Acid m	1728150	2E+06	2E+06	2E+06	2407400	1556900	1283900	1743800	1683100	2240300	1E+06	2E+06	3E+06	2E+06	1417200	2048100	1662300	1077700	2604700	1607400	1780000	1628100	1959400	1590600	1E+06	1491200	1411000

MEDN201	Succinic Acid	Amino Acid m	6220063	7E+06	4E+06	4E+06	3025500	2765800	1.4E+07	2762500	7398100	4641000	1E+07	4E+06	2E+06	3E+06	3453500	3939100	3096600	28602000	3691800	5762900	1912600	3123100	1557400	5217200	5E+06	2271900	3953000
MEDN202	A-Ketoglutari	Amino Acid m	507566.3	568930	392316	420970	518920	491210	472250	695840	537930	526880	396530	504400	578530	745050	329830	710210	417270	704330	561820	309360	385540	645240	200860	556320	434120	211490	395600
MEDN203	Cis-Aconitic A	Amino Acid m	98479.13	89698	68283	113880	214160	58649	16272	82683	92350	177120	32719	73356	144880	118490	58097	96690	67683	17641	140750	45410	104580	71939	134210	33072	48815	59844	48390
MEDN206	Citramalic Aci	Amino Acid m	3162650	2E+06	2E+06	1E+06	1226800	1929400	5280800	2988300	6646200	2291400	4E+06	1E+06	1E+06	2E+06	1160600	6119200	943230	3260600	2480100	2725000	2060400	851410	608890	2184300	2E+06	412710	1880300
MEDN209	Tryptamine	Tryptamines Ar	110285.8	64186	62855	92217	160320	133700	101950	90479	110160	124120	69340	53289	58259	75318	42039	79347	71270	71783	62184	38981	55871	65690	53051	73711	85634	52621	77282
MEDN210	1,5-Anhydro-	Carbohydrate r	374725	386168	312983	396420	449170	320340	306390	360570	454710	492030	218170	401150	517300	508010	281510	417510	310810	219600	433450	390090	353800	327850	413930	273440	188380	307440	248930
MEDN212	Dl-Threitol	Carbohydrate r	13403.15	11437	11136	13582	13087	10515	6868.2	14941	17155	13125	17952	7194.4	17028	12639	12172	18177	7550.8	4220.7	12511	12322	15109	15861	7471.3	5662.2	9948.7	7206.9	15504
MEDN213	D-Sorbitol	Carbohydrate r	127548.1	103383	101524	74909	71886	106690	153260	214190	146380	106060	147010	124550	85933	139710	80089	104470	119220	114900	58195	67504	76836	164420	76936	64643	102800	141780	117270
MEDN214	L-Arabitol	Carbohydrate r	116534.1	120819	116138	56561	69905	136730	128000	197320	107890	92477	143390	110760	130000	116990	102480	133950	154460	134840	83074	102830	114300	171260	62851	119130	134660	116470	107600
MEDN216	Ribitol	Carbohydrate r	59392.75	68881	49839	41591	43087	48351	74859	77744	56452	76197	56861	53618	44819	109190	43394	63524	55946	111030	69530	77856	66635	40238	51529	52341	48906	30913	30295
MEDN217	Xylitol	Carbohydrate r	124321.4	129542	135874	81350	96191	141570	125790	139740	145220	143950	120760	122060	132080	140930	137650	117880	160420	154570	70749	153290	133160	150630	123890	108910	188720	124220	104170
MEDN222	D-Melezitose	Carbohydrate r	59860.75	52925	64782	63392	76360	40888	71611	69213	26036	70523	60863	35188	71930	94485	42188	40518	40234	28711	70142	54279	49260	61596	54092	49676	68029	107390	73934
MEDN223	D-Sucrose	Carbohydrate r	159036.1	162721	298181	70459	149150	131430	158310	331180	110680	176060	145020	175280	168060	298440	119010	134900	137450	121680	146950	145030	157240	108840	127180	185530	113160	1416100	132370
MEDN224	D-Trehalose	Carbohydrate r	180906.3	179829	351663	148690	151810	112980	164380	352000	151220	191610	174560	148660	148290	391100	151320	141600	156930	150480	150250	160130	167410	123980	167290	211560	174320	1635000	173610
MEDN227	D-Glucose 6-	Carbohydrate r	4187575	4E+06	3E+06	5E+06	8323700	3013900	1888900	2326000	3672000	7606500	2E+06	3E+06	4E+06	5E+06	1799800	3974100	1685000	1232000	9913600	4191700	3168900	3588400	2002500	3162500	2E+06	3220300	1657100
MEDN228	D-Arabinose	Carbohydrate r	6287063	6E+06	5E+06	8E+06	8135100	6047200	4433800	5449600	6316900	7519400	4E+06	6E+06	1E+07	7E+06	6490800	8418100	4140200	2956200	2986500	6917700	4543800	4563700	4556000	4470400	4E+06	4081900	4263700
MEDN230	Lactulose	Carbohydrate r	120022.5	121008	103165	140480	165930	81737	108140	145950	121520	124130	72293	131150	128130	140960	103580	130050	127520	49784	156890	97458	119260	67534	138160	96786	92322	118980	94819
MEDN231	L-Fucose	Carbohydrate r	194622.5	205449	179188	185340	208290	181020	151570	205760	266900	228630	129470	202440	285910	245270	164500	222330	207060	143460	172620	164290	160120	210010	261360	126380	143250	212020	156070
MEDN232	L-Rhamnose	Carbohydrate r	169875	184718	145819	155160	176550	177720	132180	186820	205690	193120	131760	174400	235410	215920	171200	230630	162020	134610	153550	141910	132130	156530	226890	117770	131020	154150	106150
MEDN234	Maltotriose	Carbohydrate r	110453.3	95377	86038	111700	117420	110100	83926	108290	110560	133640	107990	115100	121960	90081	84329	120840	92195	32091	106420	105970	81530	65847	127640	85342	76406	84850	60719
MEDN235	N-Acetyl-D-C	Carbohydrate r	18288	14334	19292	9	60563	9	9	9	42174	43522	9	9	46872	9	9	9	9	9	67747	49937	43251	9	61104	9	9	9	9
MEDN236	Raffinose	Carbohydrate r	279906.3	269806	321550	230560	370600	239550	245420	351600	234680	309070	257770	297300	283080	453890	175660	261770	188440	194910	303400	242840	208730	274310	277990	236950	152220	914140	265220
MEDN237	D-Glucoronic	Carbohydrate r	6618750	6E+06	6E+06	9E+06	8607300	7746200	6143000	3515300	5258800	7100600	6E+06	4E+06	6E+06	6E+06	6544900	7789500	4310700	4636500	6963200	8536600	4809300	2285500	5178400	10431000	6E+06	5612600	8063000
MEDN239	Gluconic Acid	Carbohydrate r	23544.04	15301	14869	29443	6854.7	53312	23461	12809	24498	6325.6	31649	21971	9838.5	4469.9	39474	7763.8	12255	14157	12479	14266	5720.7	12759	9718.3	15840	11487	19310	29854
MEDN240	L-Gulonic-Γ-L	Carbohydrate r	274682.5	225934	216759	363820	384920	170310	215610	232340	344580	344280	141600	254210	292100	300150	207700	294240	159800	114190	185080	348360	225110	193640	236900	165990	134810	199700	229560
MEDN241	Vitamin D3	Co-Enzyme Fa	31893.88	33238	33955	37176	28431	34105	39736	37083	17947	28319	32354	36536	24224	62770	36537	35457	11666	30700	28015	26505	44142	13196	53111	29522	35776	36849	32536
MEDN242	L-Ascorbate	Co-Enzyme Fa	7609.488	6366.9	9	39971	9	15951	4908.9	9	9	9	9	9	9	9	15878	9	18236	16776	9	9	9	9	9	9	9	9	9
MEDN245	Pantothenate	Co-Enzyme Fa	43387625	6E+07	3E+07	4E+07	52307000	44265000	3.1E+07	4.6E+07	5E+07	5.2E+07	3E+07	6E+07	1E+08	6E+07	3.2E+07	5.4E+07	51636000	47047000	29937000	29838000	26261000	32973000	31568000	31975000	5E+07	25279000	40473000
MEDN246	Nicotinamide-	Co-Enzyme Fa	199270	196428	187186	211480	171060	199630	307640	183090	148590	203760	168910	239800	198110	266120	118410	174230	189600	210720	174430	251930	139880	166350	158490	256890	202520	121970	199460
MEDN247	Nicotinic Acid	Co-Enzyme Fa	19293750	2E+07	2E+07	2E+07	24901000	14377000	1.1E+07	1.7E+07	2E+07	2.5E+07	2E+07	1E+07	2E+07	2E+07	1.1E+07	1.8E+07	17033000	9632400	20834000	18367000	15744000	12331000	22971000	16610000	1E+07	16199000	12996000
MEDN260	3-Indolebutyr	Indole And Its I	157734	163394	161063	176620	95793	210410	245030	75032	244200	77547	137240	166830	188590	154320	142300	176100	205890	177840	95285	273260	35332	417080	39624	182430	132890	69308	138580
MEDN261	3-Indolepropi	Indole And Its I	78636.63	78140	64736	45801	61994	69844	156150	79378	79269	46908	89749	86351	100280	86933	45644	67072	54627	144170	40045	78374	48272	87844	29689	93875	91901	21056	66876
MEDN262	5,6-Dihydroxy	Indole And Its I	95251.38	104566	91523	112080	82913	87799	67178	96915	79546	128330	107250	135200	74211	101260	134260	119630	117410	47950	106610	62220	109390	101080	120590	71589	82246	82729	102340
MEDN263	5-Hydroxyind	Indole And Its I	1075778	1E+06	1E+06	1E+06	1511100	1072500	944290	873590	1138100	1073600	895540	1E+06	2E+06	1E+06	915390	1306900	1271200	843010	1377800	1449500	864460	1315600	811000	1277500	940990	855050	1065800
MEDN267	Indole-5-Cart	Indole And Its I	183330	295836	129372	206570	184520	204600	237650	190050	136240	124730	182280	386310	332280	290620	121880	257350	134090	710340	133820	127910	126800	146090	98046	161190	154600	71478	148860
MEDN271	(S)-2-Hydroxy	Organic Acid A	258443.6	123710	96219	66395	312370	55085	55190	607990	782260	188250	9	36972	159040	203250	45046	305770	9	9	239580	168760	139400	71908	62156	101430	40845	147070	38181
MEDN275	1-Caffeoylqui	Organic Acid A	103200.1	99699	92606	93924	123290	85470	88504	141450	108360	102430	82173	82537	105110	106960	88648	101120	131490	75598	106130	77277	105100	104350	97722	93335	87838	88009	87217
MEDN276	1-Naphthylac	Organic Acid A	23154.63	23728	27602	27756	18961	24716	16733	22912	24636	23040	26483	22569	24079	22231	24287	28091	20663	25066	22835	22625	28116	35931	26550	25345	22613	29861	29772
MEDN277	2,3-Dimethyls	Organic Acid A	493433.8	506595	349598	422350	468860	432990	451250	625160	678880	521270	346710	440170	466490	605260	280970	891210	351750	547330	469580	297410	329130	584570	193570	485730	366260	211620	328490
MEDN280	2-Aminoethar	Organic Acid A	18224500	3E+07	1E+07	1E+07	11859000	50385000	8143100	1.9E+07	8851900	1.2E+07	2E+07	9E+06	9E+06	2E+07	1.4E+07	1.2E+07	10153000	1.8E+08	6374300	5180500	4519400	59893000	8787500	10512000	9E+06	7627300	9032900
MEDN281	2-Aminoisobu	Organic Acid A	38583.5	40035	41824	40119	52496	33238	28999	35549	45450	43253	29564	36188	40799	35096	45962	44456	43208	26659	47909	31985	33950	51013	53743	39626	45714	39724	38836
MEDN283	2-Hydroxybut	Organic Acid A	171648.5	170399	133311	145220	90788	206530	300290	111210	108570	107650	302930	217060	102080	148570	160570	109700	110950	409440	104820	195080	46284	174730	39089	140780	164700	40517	265310
MEDN284	2-Hydroxyisoc	Organic Acid A	500423.8	588026	673505	565490	379780	564900	749510	223490	422040	219590	878590	607510	481900	406480	458160	430670	488480	1451800	379210	1989500	161370	558860	289430	758030	605770	209180	815900
MEDN285	2-Methylsucci	Organic Acid A	1990700	2E+06	1E+06	1E+06	1175900	1321100	2818000	2726100	2971900	2018600	2E+06	1E+06	1E+06	3E+06	657850	4649200	1007600	3507300	2506600	1355100	1277900	1109900	563720	1399200	2E+06	715730	1089900
MEDN290	3-Hydroxy-3-	Organic Acid A	456811.3	726854	601624	566290	457360	572870	530750	270270	601580	274010	381360	802100	1E+06	667580	551260	851690	576290	623350	723060	1024200	203020	560630	250160	849140	804730	403710	717400
MEDN292	3-Hydroxybut	Organic Acid A	11190088	9E+06	8E+06	1E+07	13233000	13204000	1.2E+07	5699600	1.6E+07	8331800	8E+06	1E+07	1E+07	9E+06	1.1E+07	1.3E+07	5946900	2819700	9655200	13362000	4687000	4711800	4379600	9653500	1E+07	5315700	9115300
MEDN293	3-Hydroxypro	Organic Acid A	12022.46	16674	11826	9525.2	11118	7809.5	9	19315	10122	16257	22024	7800.1	14778	24230	9	17820	18468	35708	14582	8293.9	11759	16187	4952	27673	12980	12755	9
MEDN294	3-Methylcrot	Organic Acid A	61639.51	79017	4038.8	16609	3554.1	291940	13131	96352	9	21788	49733	5495.4	8522.4	79413	8739.6	207240	4825.4	303990	13908	12087	9	6328.6	9	5113.4	3046.5	9	5708
MEDN297	4-Hydroxy-2-	Organic Acid A	181282.5	175695	166075	131780	255220	197560	175240	164650	233260	174080	118470	233860	197510	135380	137510	266250	94109	157970	182970	161400	113280	166740	244730	127280	164610	141040	209520
MEDN300	Azelaic Acid	Organic Acid A	66598250	7E+07	6E+07	8E+07	63286000	75777000	7.3E+07	4.8E+07	8.6E+07	5.2E+07	5E+07	7E+07	8E+07	7E+07	4.4E+07	8.6E+07	66061000	62117000	55903000	93841000	28330000	67125000	27266000	82626000	6E+07	41430000	64406000
MEDN301	Caffeic Acid	Organic Acid A	7332788	9E+06	9E+06	8E+06	7604400	8533800	8408000	4936000	8278100	5267700	8E+06	1E+07	1E+07	8E+06	8325300	7098700	5837800	10992000	9693000	14524000	4884900	6879200	5436900	10890000	1E+07	7724100	9578800
MEDN304	Cinnamic Acid	Organic Acid A	26228.13	32719	24987	22089	21934	28711	27131	28055	16480	25523	39902	26933	16217	29754	30596	25741	44622	68737	19155	17289	13934	16329	15606	31360	35011	22481	47888
MEDN305	Creatine	Organic Acid A	2916850	4E+06	2E+06	4E+06	2426800	3944100	2879700	3626300	2574400	1422100	3E+06	6E+06	5E+06	3E+06	4368500	2986700	2389400	2840500	2465000	1382600	1143000	1154700	941280	2742100	3E+06	2883900	2294700
MEDN309	D-Lactic Acid	Organic Acid A	42712.89	34873	22138	25731	10994	21597	48859	176750	9909.1	16169	31694	16701	9796.7	37976	8304.4	18392	32497	133830	21490	61629	5335.4	38255	4424.7	31308	7019.6	10324	18810
MEDN310	Dl-P-Hydroxy	Organic Acid A	701400	707231	338671	830880	1020600	696130	427250	578380	852840	898220	306900	630370	1E+06	737670	391390	1291400	516340	436960	600620	703030	221200	319440	359410	319310	258630	260490	267860
MEDN311	Dodecanedioi	Organic Acid A	4866.088	3728.4	3523.9	6351.5	4124.6	5774.5	10780	9	7113	9	4767.1	5195.2	4295.8	4509.7	6644.7	9	3695.9	5467.5	9	7213	2110.6	9	9	6440.4	5410.7	9	6989.4
MEDN312	Ethyl 3,4-Dihy	Benzene and si	27361.5	46434	53143	9	74889	9	9	56381	9	87577	9	9	54861	86161	53159	57486	28992	9	90791	45877	92730	55187	67150	55154	55740	53294	9
MEDN314	Glutaric Acid	Organic Acid A	2044838	2E+06	1E+06	1E+06	1139100	1291500	2897400	2848600	3132600	1950200	2E+06	1E+06	1E+06	3E+06	677050	4743900	1081700	3739300	2559400	1453400	1278800	1240700	587880	1540400	2E+06	749780	1244100
MEDN316	Guanidinoeth	Organic Acid A	54827.63	96203	58530	41000	21317	184370	23969	50749	33118	29623	54475	28547	29088	89566	34972	143230	48241	352620	43359	22087	5459.5	232090	13711	46036	84757	28578	35519
MEDN317	Hippuric Acid	Amino Acid m	1303892	290267	9	9	9	7340400	9	1622100	9	648050	820550	9	9	744240	9	687960	9	889890	9	9	9	9	9	9	9	9	9
MEDN319	Homovanillic A	Organic Acid A	4227638	4E+06	4E+06	5E+06	5320400	4971300	3966800	2402400	4844000	3396400	4E+06	5E+06	7E+06	4E+06	3496100	4302500	2417300	3397200	5635200	10733000	1960000	3306200	2192600	5167400	4E+06	2937600	3979100
MEDN321	Kinic Acid	Organic Acid A	982176.3	828848	827156	1E+06	1336500	1091700	836450	750350	728240	1177700	541270	869990	1E+06	1E+06	664310	695920	411220	375240	1331300	1264800	920120	802950	924200	811990	617170	621660	654360
MEDN324	L-3-Phenyllac	Organic Acid A	201956.6	237807	194106	233460	147230	212910	301310	92429	318540	57484	252290	308580	340620	180450	196370	146990	314750	321900	92793	347690	61662	152430	61570	267480	293070	64597	304350
MEDN325	L-Lactic Acid	Organic Acid A	36650.93	31223	21911	23140	9742.4	23297	41386	137540	13713	19601	24788	26126	7404.5	30948	16000	12185	27033	112850	17234	54806	7425.7	26695	8182.1	31129	12893	20664	13492
MEDN326	L-Tartaric Aci	Organic Acid A	194011.3	158306	154558	230190	289690	195280	118060	150400	238570	223670	106230	176650	265570	190530	149000	96328	92034	75957	220380	192990	132210	157660	179370	182630	136990	111580	143030
MEDN327	L-Dihydrooro	Organic Acid A	183066.3	221224	168453	176020	275560	123910	185050	214030	146560	209790	133610	191720	304570	225310	149170	241350	228360	182090	247220	159390	218540	141780	213100	183210	147870	183890	99841
MEDN331	L-Pipecolic Ac	Amino Acid m	1046325	1E+06	889275	1E+06	1212600	890430	1141100	1014500	1061200	965160	961610	1E+06	1E+06	1E+06	869750	1049600	988650	987050	1273300	1091400	825330	959420	695550	966760	783600	806840	985300
MEDN333	Malonicacid	Organic Acid A	10972200	1E+07	8E+06	1E+07	12675000	11303000	1.3E+07	5735400	1.5E+07	8241200	9E+06	1E+07	1E+07	1E+07	1.1E+07	1.2E+07	8105800	3221000	8300100	14974000	3976400	4697300	4967700	10394000	1E+07	4505900	9497700
MEDN334	Mandelic Acid	Organic Acid A	721756.3	890539	1E+06	732110	1137400	748550	293580	474030	644310	1451500	292570	249880	1E+06	1E+06	447920	1540100	952690	856650	665870	663130	1052900	3620600	927680	772380	346670	579960	336340
MEDN335	Methylmaloni	Organic Acid A	81647250	8E+07	5E+07	5E+07	43674000	43095000	1.7E+08	4E+07	9.5E+07	7.1E+07	1E+08	6E+07	3E+07	4E+07	4.3E+07	5.9E+07	42702000	3.03E+08	53012000	64990000	25769000	42568000	21627000	69331000	7E+07	29120000	52329000
MEDN338	Phenyllactate	Organic Acid A	125061.3	191075	160197	136290	106450	148080	152080	96890	134430	102500	123770	168440	161430	146620	122310	322780	146750	334720	125550	340590	49522	367040	83888	121900	133690	56198	128750
MEDN339	Phenylpyruvic	Organic Acid A	419576.4	331589	344596	527600	161750	939050	869770	111520	196880	62061	487980	332650	217470	150280	392790	369850	613900	575760	9	405060	9	9	9	708520	620180	97210	925770
MEDN340	Pyrrole-2-Car	Organic Acid A	15945.13	18433	13911	16979	11231	15986	29310	10470	12404	14384	16797	15358	20733	18973	26020	13210	13806	27356	12010	21609	9	15810	9	18547	20434	7555.1	27316
MEDN342	Sebacate	Organic Acid A	15842.96	15976	18921	16995	12838	27016	18555	10994	19353	8910.7	12082	14268	21330	12232	11304	24080	22363	14321	7909.1	20894	4430.4	64680	5100.7	17832	14310	8921.6	15202
MEDN344	Subericacid	Organic Acid A	1744413	2E+06	1E+06	2E+06	2091500	1675200	1526300	1513400	2071600	1872800	1E+06	2E+06	2E+06	2E+06	1229500	2450600	1799200	1324900	1770000	1789400	988800	1964500	1119500	1715600	1E+06	1177800	1489600

MEDN347	A-Hydroxyiso	Organic Acid A	80471.13	45358	33189	14126	92028	18362	9	182550	258650	61848	16196	21684	33181	50400	7717.7	123610	14656	21391	90221	77685	45472	28193	16143	39606	9900.6	38482	10032
MEDN350	(±)15-HETE [(	Oxidized lipid	24516.63	22990	22710	21699	23208	30846	23017	24466	20683	18343	33871	34199	17638	18007	15587	30514	19120	29205	19653	24841	13517	27425	15816	25751	26130	23931	24269
MEDN351	12-Hete	Lipids	14395.13	16162	14264	11823	12540	16319	10649	15841	16089	17213	14687	24124	14165	13138	13988	15010	22205	17482	9181	7157.8	8509.2	25516	15428	13201	17205	17142	9953.2
MEDN352	O-Phosphoryl	Lipids-Phosphc	531863.8	604339	599573	604870	757420	421170	315440	563730	542270	692370	357640	433280	820440	839830	350900	779060	358080	165620	1087500	555550	1056700	704880	722590	506240	320800	594880	334940
MEDN362	Lysope 18:1	Lipids-Phosphc	47088500	4E+07	4E+07	5E+07	36155000	48962000	4.6E+07	4.3E+07	6.5E+07	3.4E+07	5E+07	4E+07	4E+07	3E+07	4.5E+07	4.9E+07	42621000	39700000	26692000	34560000	32581000	44009000	29306000	54692000	4E+07	30759000	43741000
MEDN364	Lysope 18:0	Lipids-Phosphc	10891288	1E+07	1E+07	9E+06	4551800	13318000	1.2E+07	9751800	8993100	5335500	2E+07	1E+07	6E+06	7E+06	1.1E+07	1.3E+07	9210500	20764000	5711600	6935800	4468400	12601000	5429000	20186000	2E+07	11930000	7744800
MEDN366	Lysope 16:0	Lipids-Phosphc	91592250	9E+07	9E+07	1E+08	64585000	108360000	1.3E+08	6E+07	9.6E+07	7.5E+07	1E+08	8E+07	9E+07	1E+08	6.5E+07	1.1E+08	80156000	1.02E+08	66197000	1.07E+08	79079000	90823000	58831000	115640000	9E+07	86152000	1.02E+08
MEDN368	Lysope 14:0	Lipids-Phosphc	30442375	3E+07	3E+07	4E+07	24903000	34645000	4.5E+07	1.8E+07	3E+07	2.5E+07	3E+07	2E+07	4E+07	3E+07	1.9E+07	3.8E+07	26352000	31504000	23953000	44941000	29052000	37782000	17786000	39550000	3E+07	26553000	33443000
MEDN370	Lysopa 18:0	Lipids-Phosphc	44588.38	53317	57785	33306	9	44874	46217	66476	41156	9	124660	90718	33101	34219	56579	42153	56456	76887	36424	26028	20674	74220	17801	114140	88748	64651	56016
MEDN372	Lysopa 16:0	Lipids-Phosphc	128339.3	178149	138771	92764	47760	133510	138030	167110	114040	49270	284230	224880	90151	87794	138910	141170	206270	492970	43043	42035	55239	172930	52965	238840	286940	111730	149490
MEDN373	LipoxinA4 [5S,	Oxidized lipid	21475.38	26571	20265	24733	24442	25082	20109	18015	25391	15503	18528	30102	30334	33286	18305	23072	38273	22547	16647	16899	11376	32561	10656	28874	22099	17477	22179
MEDN375	13-HOTrE [13	Oxidized lipid	115894.1	138313	97114	89150	58861	128130	165880	128640	118530	62352	175610	142050	71221	100840	104380	135900	251080	242530	58499	115350	57951	122270	29252	100700	134710	76900	139780
MEDN376	9,10-DiHOME	Oxidized lipid	2066650	3E+06	2E+06	2E+06	2310900	1924900	1841100	2935500	1652500	2155500	2E+06	2E+06	3E+06	5E+06	1230100	1917700	3155500	2261700	1900800	1769400	1956700	2735400	1579500	2023700	2E+06	1598200	1803700
MEDN378	γ-Linolenic Ac	Lipids_Fatty Aci	163379.4	174207	165869	126100	77806	198940	233080	126460	147340	75569	321740	194960	115940	119680	169060	169960	279300	288030	56728	175810	86177	182530	49802	178700	263370	147910	242650
MEDN379	Tridecanoic A	Lipids_Fatty Aci	18799.63	12559	14992	16057	11967	34180	32885	15175	13811	15365	10957	11902	14263	13203	8518.9	14759	17876	11647	8304	22907	14345	18610	12529	14383	12389	9945.4	14826
MEDN380	Palmitoleic Ac	Lipids_Fatty Aci	2483988	2E+06	2E+06	2E+06	1390500	2700000	2637500	2552900	3269900	2097500	3E+06	3E+06	2E+06	2E+06	2024000	4431300	2340400	2879700	1374100	3326200	1212300	2647200	1695200	2159900	4E+06	2000900	2789000
MEDN381	Hexadecanoic	Lipids_Fatty Aci	105211.5	69668	120517	64035	43379	135030	67818	119430	152830	150150	109020	70037	71275	66477	60115	90663	74182	56093	68498	228000	97770	80751	71024	80961	131310	116770	157550
MEDN383	Linoleic Acid (	Lipids_Fatty Aci	12236425	1E+07	1E+07	1E+07	8322100	10978000	9788300	1.3E+07	1.4E+07	1E+07	2E+07	1E+07	1E+07	7E+06	1E+07	1.4E+07	11523000	13015000	8468000	15579000	6711600	11157000	5628600	8872500	1E+07	15769000	11322000
MEDN385	Dodecanoic A	Lipids_Fatty Aci	170692.5	164088	203401	194940	102000	186540	213520	181050	164910	128060	194520	151960	149580	167800	157660	162200	121580	247660	154260	366840	121280	162210	138060	219460	225780	162320	231260
MEDN388	Elaidic Acid (C	Lipids_Fatty Aci	33642	27468	29453	25212	18501	34286	23726	27427	48070	27923	63991	39889	25627	23009	29165	26960	32568	25383	17146	27712	18057	23356	16751	24902	38837	41139	44868
MEDN390	EPA [5Z,8Z,11	Oxidized lipid	13123475	1E+07	1E+07	1E+07	11606000	18728000	1.2E+07	9788800	1.3E+07	1.1E+07	2E+07	1E+07	1E+07	1E+07	1.2E+07	1.6E+07	14106000	15401000	11404000	10638000	9050600	13061000	7442300	16603000	2E+07	17525000	14806000
MEDN391	DHA [4Z,7Z,1(	Oxidized lipid	517868.8	505180	564761	472460	408620	647520	446460	451290	638030	396140	682430	578330	438990	359590	493030	670310	639130	560520	301540	437100	300250	604960	324800	662300	859640	713000	616040
MEDN394	Cis-11,14-Eicc	Lipids_Fatty Aci	4426.025	4202.7	3596.6	5009.8	3146.1	6206.9	5095.2	2623.3	4496	4532.9	4298	3646.2	3848.1	3598.5	4694.6	5199.5	4887.7	4339.4	3407.9	3688.1	2610.5	4725.6	3058.9	4196.3	3611.7	2491.7	4390
MEDN395	Cis-11,14,17-I	Lipids_Fatty Aci	315696.3	350139	355683	305460	305200	264000	358550	323670	304450	354710	309530	304110	363070	351030	294780	409610	419580	323420	335510	277130	452450	299670	361520	400290	330460	330440	393500
MEDN399	Arachidic Acid	Lipids_Fatty Aci	925500	1E+06	871144	940450	431750	1724700	1196700	717140	994030	405530	993700	942730	662160	464890	1111700	1042700	1268700	2402800	391930	1214000	351450	1141800	415940	826120	1E+06	403440	1137600
MEDN400	A-Linolenic A	Lipids_Fatty Aci	212545.6	220246	211964	144720	93579	260550	298690	162800	186220	93126	460680	241790	142980	151190	189240	213560	340810	394030	88369	227660	108750	228910	64180	219430	348520	195290	302970
MEDN404	(S)-(-)-2-Hyd	Organic Acid A	2871038	3E+06	4E+06	3E+06	2152100	3880800	4228400	1371100	2443800	1179500	5E+06	3E+06	3E+06	2E+06	2474300	2435800	2663900	9366900	2197000	13948000	1092400	3847500	1660000	4243000	3E+06	1140300	5591200
MEDN405	D-Xylonic Aci	Organic Acid A	9990663	7E+06	8E+06	1E+07	11020000	10826000	9630200	5004900	1.1E+07	1.2E+07	9E+06	7E+06	1E+07	8E+06	8077800	9158100	2583800	2973000	10554000	21022000	6359800	6124700	5312000	7271700	4E+06	5097700	6117100
MEDN406	3-(3-Hydroxy	Organic Acid A	159682.3	6E+06	2E+06	54720	14363	646430	44982	136280	149770	62633	168280	1E+06	290690	136110	115510	5760300	25217000	13006000	80441	40542	5343.6	18933000	5501.3	20972	16113	6349	19609
MEDN407	3-Aminosalicy	Organic Acid A	137363.3	224286	128621	138110	220090	106380	102390	91409	172550	191470	76507	220380	308760	257180	119050	292280	299900	143430	153310	99782	83592	371090	109220	103620	82389	80013	99263
MEDN410	4-Hydroxyber	Benzene and su	435093.8	502880	504929	455880	408440	531430	394660	484480	314090	397430	494340	413510	404250	634320	371560	500490	665690	721280	311940	415150	341330	795480	370760	558230	583410	393850	581220
MEDN413	Ethylmalonate	Organic Acid A	47333.38	61896	37523	27903	29570	29080	51047	100030	69468	35989	35580	44495	27668	64660	24673	105760	40111	150270	37531	52489	43388	60366	34362	33277	39136	14162	23001
MEDN414	4-(Aminometl	Others	69423.75	88870	71182	61370	112840	69118	81114	53141	52451	98108	27248	80717	162880	135460	30237	70304	90336	49111	91913	51466	109120	45491	85164	55260	73912	77573	71468
MEDN416	Ureidoisobuty	Organic Acid A	336828.8	422560	244974	280710	377060	410790	159050	349390	558860	371770	187000	663340	397310	340410	494920	601320	481820	132370	268990	276190	160710	323530	312630	289260	186550	222730	188190
MEDN417	(Rs)-Mevaloni	Organic Acid A	242304.4	299853	125576	178400	396850	213710	136150	189100	369790	357390	97045	416580	481450	321730	172900	480810	167750	157220	200380	150690	110030	142530	149520	141350	63084	114600	132800
MEDN419	Uridine 5-Mo	Nucleotide met	168442.9	187493	174530	169290	147570	46898	25275	131230	440570	343510	43200	41868	131140	119130	635400	133690	26243	10665	401810	130970	84437	902480	140880	39902	20861	56578	20134
MEDN422	Deoxyadenosi	Nucleotide met	78965.63	82991	84313	121160	153600	38783	46212	37795	76996	125140	32039	30304	206180	168290	56824	77160	64992	23236	36939	144470	144770	30610	98300	93761	53137	34328	75130
MEDN425	Trans,Trans-M	Organic Acid A	33764375	3E+07	3E+07	4E+07	22797000	40481000	4.2E+07	2.1E+07	3.5E+07	2.7E+07	4E+07	3E+07	2E+07	2E+07	4E+07	2.3E+07	28402000	42571000	19037000	39579000	15377000	32286000	24021000	37088000	4E+07	29943000	40090000
MEDN426	2-Methylgluta	Organic Acid A	517255	546360	354759	373290	497070	477100	495650	657620	732000	552420	352890	493120	561410	685570	276540	845860	378310	632570	497500	267480	315680	541400	221620	527890	389000	233090	341910
MEDN428	N6-Succinyl A	Nucleotide met	564078.1	324008	285370	251970	69755	511730	1190200	725110	672110	198840	892910	226360	66000	141940	824360	169220	159420	884710	120050	585810	108480	110940	167240	292130	337420	73433	607510
MEDN430	Thiamine Tripl	Co-Enzyme Fa	7774.15	8641.7	10519	7498.8	8952.6	7797.1	6028.6	6882.8	9306.6	8401	7325.7	8187.5	8850.3	8840.1	7035.2	9078.5	9825.2	8397.3	8919.5	9515.5	9958.6	14228	9653.5	10215	11559	10456	8566.5
MEDN432	5-Hydroxyhex	Organic Acid A	310395	345766	299176	343750	268880	407460	459110	179240	391740	141730	291250	382690	394180	267030	321110	345690	358460	486940	210030	484540	58540	507380	72236	373490	326590	186960	383670
MEDN434	B-Pseudouridi	Nucleotide met	1153481	1E+06	861421	850490	1181300	1614100	776370	1611100	998800	1612800	582890	882670	818740	1E+06	538990	1525900	1045300	1184200	1088200	1277500	866710	721420	992460	852460	668030	817090	695700
MEDN438	Methyl 3-Hyd	Benzene and su	180421.6	6E+06	3E+06	54516	17750	746150	44797	146500	183330	65880	184450	622910	301320	140560	126590	6469400	29242000	14662000	78749	48073	8004.5	21732000	5033.2	20250	19394	6910.4	25063
MEDN443	(S)-β-Aminois	Organic Acid A	41959.38	35097	32704	39563	29648	45685	47917	36965	72143	33079	30675	30206	39487	25343	38124	40899	58793	16278	31643	28259	29364	24929	15972	42958	46905	41204	32043
MEDN444	Hypoxanthine	Nucleotide met	86250750	9E+07	3E+07	9E+07	1.22E+08	60600000	5E+07	8.6E+07	9.3E+07	1.5E+08	4E+07	8E+07	1E+08	1E+08	6.6E+07	1.1E+08	71201000	30198000	121130000	3769300	19296000	80059000	4957300	19195000	3E+07	77556000	35694000
MEDN461	D-Sedoheptu	Carbohydrate r	36869.53	24589	29703	42359	17116	50948	60920	7768.2	44039	21414	50392	33316	17337	7861.9	77985	11869	26257	10999	11084	43599	5195.8	28081	3482.2	30909	41602	24489	60262
MEDN462	D-Glucopyran	Carbohydrate r	1979463	2E+06	2E+06	2E+06	2216200	1749000	1422200	1586600	2333700	2389700	2E+06	1E+06	2E+06	2E+06	1596200	2088900	1380800	904230	1998000	2226400	1447900	1325700	1556300	1572300	1E+06	1482200	1640200
MEDN463	D-Fructose 6-	Carbohydrate r	5289363	6E+06	4E+06	6E+06	9327800	3564900	3135500	4583300	4820900	8181800	3E+06	6E+06	9E+06	8E+06	2628700	6038300	3044700	2152400	11260000	4714900	3539300	3811000	4005300	4200700	3E+06	4457600	3088500
MEDN466	Deoxyribose 5	Nucleotide met	80063	91419	62281	95792	154900	54159	53207	70538	61896	114860	35152	151580	182050	118890	43404	78333	79236	29356	48503	92660	53789	83663	47147	58872	58530	55915	47671
MEDN469	Citraconic Aci	Organic Acid A	1006515	793693	643280	871340	741970	955460	1203800	800240	1662100	1000500	816710	542330	805430	881820	627740	1383900	323160	873260	911900	1153400	567160	523160	611280	718030	568430	359000	645780
MEDN470	Oxoadipic Aci	Organic Acid A	72280.38	90466	115063	67459	55154	73473	131760	51352	63703	43523	91819	78438	71991	74799	92865	81692	103040	160920	59985	96789	74890	170490	75871	130580	143970	90976	136940
MEDN472	1-Methyluric /	Organic Acid A	241028.8	245905	176023	293430	170270	245180	238990	234610	344350	229760	171640	323010	339920	204430	241560	374920	210800	113410	159190	314810	79845	194450	116710	199960	146920	99852	255640
MEDN478	Aminomalonic	Organic Acid A	2893225	3E+06	1E+06	2E+06	1398000	1345200	6734100	1220000	3488500	2315900	5E+06	2E+06	1E+06	2E+06	1369400	1780300	1522500	12388000	1819200	2441100	821140	1377500	620170	2205000	2E+06	835260	1539200
MEDN481	2,4-Dihydroxy	Benzene and su	87620.5	51491	53963	56753	33665	52615	76258	115120	187500	65643	113410	104280	22162	32455	57544	80549	25635	19724	69575	68326	56966	42513	78822	55273	48858	20371	60573
MEDN484	B-Hydroxypyr	Organic Acid A	12136000	1E+07	8E+06	1E+07	15776000	12633000	1.4E+07	6539900	1.6E+07	9175500	1E+07	1E+07	1E+07	1E+07	1.2E+07	1.1E+07	7711400	2840300	9858300	14892000	3861400	4490000	4846500	10851000	8E+06	5269500	10183000
MEDN485	D-Fructose-1,	Carbohydrate r	143934.5	130470	144511	166790	145550	175820	157410	144980	116250	151310	93366	92383	105050	183250	112520	176140	113330	97416	163670	193980	112730	124150	177210	161510	117330	149070	120110
MEDN486	Palmitaldehyd	Lipids_Fatty Aci	3020038	3E+06	2E+06	3E+06	2734900	2986800	3347200	2775300	2900100	3086500	4E+06	2E+06	3E+06	3E+06	2373200	2454000	2303100	3258600	3035000	1795200	2137800	2183100	1711300	2298200	2E+06	2827200	2891100
MEDN487	Allysine	Organic Acid A	154107.9	125353	132884	179930	246840	225230	102740	90459	113020	201930	72714	110070	153150	105710	67107	199270	66587	87002	213930	276970	127480	141290	102510	109910	96097	90483	118330
MEDN491	9-HOTrE [9S-	Oxidized lipid	183415.5	194406	109287	87504	105940	103660	253430	347000	134220	102500	333070	254390	92920	250380	104200	198630	357200	206120	91407	98596	99756	126110	30186	103210	191260	99549	125630
MEDN493	4-Methoxycin	Organic Acid A	120139.6	175028	155941	124670	95437	125560	90601	134920	81899	111040	196990	115980	94155	197110	64162	102860	154350	336300	335310	154080	76434	641460	69069	106620	76181	58755	64926
MEDN494	8,15-Dihete	Lipids_Fatty Aci	757833.8	737100	780531	614650	492630	627230	720750	795590	1136300	764540	910980	776160	635360	588500	564290	1445900	624190	744850	517550	1058300	440080	806940	571400	641720	1E+06	754890	743220
MEDN496	N-Acetylmeth	Amino Acid m	109660.9	262090	106069	92926	138810	93006	164690	86004	50818	91343	159690	257240	102570	210270	103410	226480	189250	765830	241670	106490	63367	121100	72048	171100	116330	58348	139770
MEDN498	Ribulose-5-Pl	Carbohydrate r	2918800	3E+06	2E+06	3E+06	5001100	2438700	1813700	2382700	2818900	5012400	1E+06	4E+06	7E+06	5E+06	1366800	4593500	2033100	1158900	1629200	1953200	1467400	1915500	2360400	1523300	931150	1655400	1397600
MEDN499	Argininosuccir	Organic Acid A	418753.8	325022	249919	218270	627930	25050	9	67434	231810	2084300	95227	61967	220530	577210	232650	206280	9	8532.2	1293000	642920	142630	620910	92495	161240	30732	268220	40201
MEDN501	Acetyl Tryptop	Amino Acid m	184505.5	187313	169098	233850	248930	195480	115790	189030	202680	193980	96304	162640	228000	247230	146160	211190	153430	145440	204410	197400	149430	175200	193820	189720	150440	161770	135000
MEDN502	2-Deoxyribose	Carbohydrate r	132891.6	133430	95859	115250	268200	74425	92244	61255	61385	345000	45374	135090	184320	183780	104280	100990	54442	50579	253960	184620	94162	96625	82302	116360	64151	69279	59373
MEDN506	N-Acetylgluco	Carbohydrate r	341610	478852	508348	334050	504560	372740	216730	246720	525290	261610	271180	473990	184370	235050	234990	1736400	79428	537680	348910	948960	953830	531930	724970	139990	220690	251310	295100
MEDN507	2-Isopropylma	Organic Acid A	6534.888	5136.3	8464.2	9	9799.8	8245.5	5240.7	5407.3	8138.9	6562.8	8875.1	14654	6680.3	9	3358.6	9	9	4589.7	11781	12170	4889.5	7517.1	11084	8814.1	7398.7	3978.8	11861
MEDN523	Indolelactic ac	Indole And Its I	170202.5	194255	123146	191300	238730	122960	142930	147200	164880	220840	132780	197380	222600	261260	137800	164100	199730	150870	220300	185230	101760	144140	100310	115380	112770	77125	148450
MEDN524	Estrone	Hormones	9838.913	7325.5	7359.5	11649	14425	7890.3	8091.8	8612.3	10821	8541.4	8680.5	9	12247	10781	9393.6	8653.1	4381.3	3049.9	10089	8497.1	4696.2	7543.8	6125.9	6774.5	8264.7	10976	5997.4
MEDN530	Pimelic acid	Organic Acid A	1461798	1E+06	1E+06	1E+06	1740000	1493900	1146700	1450000	1634800	1827800	947580	995190	1E+06	2E+06	901550	2225200	1389600	1153100	1647000	1207300	1019300	1418800	971820	1139800	1E+06	889690	1067400
MEDN531	D-Glucarate	Carbohydrate r	522170	381468	397183	479430	574550	528150	472960	411670	667480	641270	401850	275820	396320	488570	330090	493870	298510	187530	581030	707570	341500	356060	315570	456450	361410	331520	307380
MEDN533	Xanthosine	Nucleotide met	13977200	1E+07	5E+06	2E+07	28749000	1.20E+07	1.3E+07	4938400	7888500	1.8E+07	7E+06	4E+06	2E+07	2E+07	7404700	1.5E+07	5966200	4470000	14745000	2754100	1832500	2678600	994390	11098000	7E+06	7742100	8619300
MEDN537	ADP-ribose	Nucleotide met	39905.5	36202	155847	33692	26619	28083	14730	35265	65314	91872	23669	12703	36616	47609	52196	41190	9	9	99286	48391	29890	1085300	19157	20943	9721.6	11607	21769

MEDN538	UDP-glucose	Nucleotide met	11614.08	12668	42253	8938	8634.8	9	5899.8	9	24637	31292	13493	4448.4	9	18588	11855	16523	9	3616.5	46293	45492	10872	276660	9	4963.5	9	9	9
MEDN540	1-Methylguan	Nucleotide met	345218.8	438570	308519	376600	467780	328230	202060	422130	339250	395570	230130	557850	638670	423510	346780	393860	509390	358530	279970	272720	390130	261750	422140	255340	279970	317750	268350
MEDN541	DL-3,4-Dihyd	Benzene and su	513802.5	525643	441213	643670	487880	568570	581740	376820	671580	399940	380220	491160	648950	542150	357240	690710	531030	502700	441200	679670	224280	504470	202300	654110	443330	313730	507810
MEDN542	3-Hydroxy-4-	Benzene and su	4215425	4E+06	4E+06	5E+06	5199800	5117100	3995600	2261700	5091400	3325500	4E+06	5E+06	7E+06	4E+06	3543600	4335700	2507100	3362300	5553700	11351000	1957200	3144600	2232500	5397500	4E+06	2986800	3936600
MEDN543	3-Methyladipi	Lipids_Fatty Aci	1476618	1E+06	1E+06	1E+06	1743600	1467800	1146100	1535600	1605700	1877700	954740	1E+06	1E+06	2E+06	891490	2168600	1332900	1202000	1698200	1210700	986370	1379200	986080	1116200	973360	845150	1038700
MEDN544	3-Methylgluta	Lipids_Fatty Aci	487870	522614	333905	390700	490770	410110	419210	632730	710870	504300	344270	428150	496640	654760	276660	853960	384190	598330	488220	269060	310950	447430	192780	515880	377450	207850	349840
MEDN548	Galactaric acic	Carbohydrate r	496017.5	367781	374313	454720	573440	596620	364710	397970	562260	623620	394800	254730	312680	490760	337310	482880	290770	203840	569280	703850	236420	273980	353830	405290	320520	391180	309430
MEDN550	2-Phenylbutyr	Benzene and su	4682625	4E+06	4E+06	4E+06	3163000	3951600	8151000	4291600	3158200	2904500	8E+06	4E+06	3E+06	4E+06	2879800	2420300	4299900	6486700	1218600	7128900	3251300	1306100	3355900	5645800	5E+06	1506000	5410100
MEDN551	Indoleacrylic a	Organic Acid A	129225.5	108234	583979	159450	216700	76546	72428	119350	106970	136790	145570	106140	119080	148610	109130	109680	111300	42069	119860	1290400	1050800	838830	1233500	41500	111020	44728	61050
MEDN553	2-Methylbenz	Benzene and su	1849838	2E+06	3E+06	1E+06	1312200	2365100	2285300	1790900	1725700	1729000	2E+06	2E+06	977710	1E+06	2121100	1457700	1972600	2305900	1278300	3018500	3063600	993390	4070700	2920400	2E+06	1854200	2717700
MEDN554	2-(4-Hydroxy	Benzene and su	17231288	9E+06	2E+07	2E+07	11437000	18140000	3.1E+07	7425400	2.2E+07	6658900	2E+07	2E+07	8E+06	9E+06	1E+07	6409400	8138400	6196800	8913300	23057000	4951100	8766100	4141600	22245000	3E+07	6389900	25955000
MEDN555	Hydroxypheny	Carbohydrate r	661376.3	677055	330043	790950	995310	680270	418910	555230	741250	774880	334210	612430	1E+06	742820	365870	1198400	487590	433360	522170	677570	210910	346550	342150	315840	255560	227280	264480
MEDN556	Cinnamoylglyc	Organic Acid A	23086.73	49524	7446.6	27658	47330	12808	8875.8	14041	35126	25981	12874	73562	145840	52749	30029	46745	16537	23832	6896.1	6481.4	8008	9129.1	8254.6	9565	4310.9	8667.5	5155.9
MEDN557	cis-5-dodecer	Lipids_Fatty Aci	937936.3	887200	797104	1E+06	1126800	897780	767230	811420	936640	1285300	606620	743770	935120	1E+06	667480	1069200	830650	543480	1186800	880630	712300	698610	886860	898610	748750	806020	745050
MEDN560	MARMESIN	Carbohydrate r	2069313	3E+06	3E+06	2E+06	3112300	1695300	1306700	1771600	1984200	3455900	1E+06	2E+06	3E+06	4E+06	1456600	4124000	3967000	1210000	3916000	2188800	3153600	7369400	3007400	1753500	1E+06	1961100	1223400
MEDN561	N-Acetylaspai	Organic Acid A	137460.9	116724	124412	195400	157790	140130	87597	146720	125420	135780	110850	90084	111690	126520	107910	91013	98975	98700	208900	184350	156390	111200	148080	154400	9	139360	101510
MEDN562	INDOLE-3-CA	Indole And Its I	86502.5	84265	79764	78696	110750	78397	78984	79962	102290	106260	56681	46063	98898	104030	59967	123200	86602	58530	96826	85520	61613	64489	71264	113810	95201	53208	93006
MEDN563	DL-Benzylsucc	Lipids_Fatty Aci	89152.38	98660	55153	71958	83021	61522	160260	90128	154230	40582	51518	276230	23648	48332	53319	31948	202530	57076	96193	138680	56518	21693	63317	30166	61176	37938	31737
MEDN565	cis-7-Hexadec	Lipids_Fatty Aci	20101500	2E+07	2E+07	2E+07	11187000	21913000	2.2E+07	2E+07	2.7E+07	1.6E+07	3E+07	2E+07	2E+07	1E+07	1.7E+07	3.5E+07	19896000	22960000	11032000	26675000	9736300	22307000	13810000	16901000	3E+07	16773000	22653000
MEDN566	2-Acetyl-5-te	Organic Acid A	144714.5	144040	136696	196780	211130	138320	130850	81127	146680	157000	95829	127900	156170	151710	117410	234250	127250	84608	153020	258860	104930	190340	161580	114150	88704	102940	72063
MEDN568	2-Methylguan	Nucleotide met	215505	211509	77979	224190	191820	331740	246940	175210	135260	217630	201250	195080	175390	220510	189650	231820	179630	358540	141450	57540	41409	81864	41412	82686	133380	54249	131290
MEDN569	DL-o-Tyrosine	Organic Acid A	455220	508949	425434	322830	500940	616680	367300	438810	359640	579300	456260	457170	394340	440100	436840	483710	619820	959860	279750	319620	305760	564560	415560	393370	455740	393670	555190
MEDN570	L-Erythrulose	Carbohydrate r	419450	372491	324239	558690	565160	549140	388160	230320	447750	326900	289480	380000	524910	349290	340870	369230	344060	250270	421300	677440	144090	265720	165690	336540	412200	236680	355550
MEDN572	cis-4-Hydroxy	Organic Acid A	72036.88	96727	74858	60567	68529	83849	76551	99984	42044	57178	87593	87492	59442	76557	78123	90333	131790	224530	25546	59979	57026	46775	52080	122040	87453	70097	103410
MEDN574	7-Methylguan	Nucleotide met	254677.5	249896	84758	275870	208880	409620	257700	190190	197720	243330	254110	216640	206030	253290	222610	292580	207900	450870	149250	64596	61914	82954	39848	91585	134480	60468	142220
MEDN579	N-lactoyl-phe	Organic Acid A	41980.75	59364	37283	49208	47912	20351	27670	40491	29528	90571	30115	49495	46560	62222	27347	78570	68812	53534	88372	29119	29730	35059	37882	45832	41855	30457	48330
MEDN580	D-Mannose 6	Carbohydrate r	4025575	4E+06	2E+06	5E+06	8377400	2415900	2087800	2669400	2727200	6782500	2E+06	3E+06	4E+06	5E+06	2007700	3780500	1818600	1139200	9873500	4502700	1789500	2446900	2224400	2496700	2E+06	3142600	515060
MEDN587	N-Acetylvaline	Organic Acid A	252927.5	316414	237603	288610	311350	279640	158310	280330	256220	275910	173050	174180	268220	339080	205150	290320	259390	710210	284760	244940	261470	252230	286710	239920	171570	224720	219260
MEDN588	Glycerol 3-ph	Lipids_Fatty Aci	502325	523725	687218	528520	805900	264220	218870	361670	649090	974350	215980	240430	552640	740100	629070	496450	220670	146640	1163800	1479100	936290	995140	564000	361540	152470	701180	308020
MEDN589	Cyclamic acid	Organic Acid A	83100	234164	96188	76690	102870	76602	76785	92988	108070	90112	40683	403830	187450	151650	66748	389610	191930	99853	382240	60411	39744	244450	31080	132920	69836	112890	78170
MEDN593	D-(+)-Malic a	Amino Acid m	163577.1	173413	187258	188580	155220	152270	225790	160740	90157	156010	179850	85483	153180	197450	227270	190210	171910	175590	186210	132450	126210	95600	137830	350760	178970	184120	292120
MEDN594	m-Coumaric a	Benzene and su	4724913	4E+06	4E+06	4E+06	2820400	3912400	8121000	4410400	3246400	3058300	8E+06	4E+06	3E+06	4E+06	3074700	2340600	4517900	6303400	1276800	7099200	3366400	1437500	3246100	5640900	5E+06	1523300	5400300
MEDN596	D-Fructose 6-	Carbohydrate r	3793538	5E+06	4E+06	5E+06	8283500	2583400	2078700	2596900	3331100	5440700	1E+06	3E+06	5E+06	5E+06	2172400	3755400	1917300	1192100	14880000	6117200	4101200	3223800	2620900	3531400	2E+06	4007200	2276500
MEDN602	deoxyguanosi	Nucleotide met	340354.1	287822	558056	323710	260710	118790	79393	432770	652830	680290	174340	67562	272500	333280	694750	283610	30644	41716	578510	434480	355610	3076500	218030	106130	40938	161670	71087
MEDN603	2,3-Pyridinedi	Pyridine And Py	52573.75	45132	45387	65589	9	76649	65522	43880	49049	78069	41823	49077	9	50606	66223	58717	49779	29463	57184	68055	36326	56751	43939	45972	38338	31276	42441
MEDN604	Tetradecanedi	Lipids_Fatty Aci	118118.4	112325	92638	185570	119920	125260	116310	90854	105200	108810	93023	104470	89577	93314	127180	147060	116660	117600	102740	127580	52958	102310	83023	116420	93922	64884	100010
MEDN605	Tiglic acid	Lipids_Fatty Aci	465704.9	429289	437412	723260	301200	1078600	416060	9	679330	276160	251020	451160	670570	224140	942680	387590	9	508420	249740	1011400	9	358300	258520	438990	408100	331240	692740
MEDN606	5-Methoxytryp	Indole And Its I	23170.13	15507	1374.3	21348	26929	23222	21652	27356	9	39918	24927	20997	9	24811	23591	9	17234	9	37392	9	9	9	10931	9	9	9	9
MEDN607	Salicylic acid	Organic Acid A	17735063	9E+06	2E+07	2E+07	11780000	18707000	3.2E+07	7696700	2.2E+07	7040800	2E+07	2E+07	1E+07	9E+06	1E+07	6786900	8385300	6391700	9102700	23920000	5103500	8955600	4311600	23348000	3E+07	6533100	27125000
MEDN611	6β-hydroxytes	Hormones	5627.738	6430.5	8116.2	4051	10122	3606	5733.8	6708.7	3572.7	7450.2	3777.5	8647.8	3823	5132.8	7375.6	8295	3994.2	6573.6	7602.2	11922	15335	5499.3	9	5540.8	4753.8	6226.3	15643
MEDN613	2,2-Dimethyl !	Lipids_Fatty Aci	503483.8	517453	355503	408790	455480	468270	468110	656310	703010	536890	331010	453200	528270	629060	301630	807390	386910	544580	488580	279950	327880	585250	205840	482080	405590	235530	321900
MEDN614	Dimethylmalo	Organic Acid A	1876500	2E+06	1E+06	1E+06	1107900	1331200	2662200	2507000	2733900	1880400	2E+06	1E+06	1E+06	2E+06	631870	4260900	969250	3278800	2417500	1282500	1247600	1082000	509910	1377400	1E+06	677980	1092900
MEDN615	Carbamoyl ph	Organic Acid A	2058950	2E+06	2E+06	2E+06	2722200	1660600	1310200	2019100	2008200	3157500	1E+06	1E+06	3E+06	3E+06	1554200	3029800	1327500	482180	4222600	2229600	3680700	2772600	2637600	1960300	1E+06	2324800	1212700
MEDN617	D-Ribose	Carbohydrate r	5917650	6E+06	5E+06	7E+06	8229400	5785600	4600800	3855500	6837700	7617900	4E+06	6E+06	9E+06	6E+06	5741700	6395000	3826500	2886800	6340300	6884500	4794000	4638200	4708300	4906000	4E+06	4350500	4528500
MEDN618	Dipicolinic acid	Pyridine And Py	46781.5	39775	41544	69150	59526	58076	33951	39509	30292	61165	22583	45432	9	84262	33960	34308	37463	17232	65534	49246	36048	37357	42629	49392	33764	39006	44912
MEDN623	Salicyluric acic	Benzene and su	1943405	3E+06	661669	2E+06	3894000	1169600	972840	1307400	2603300	2590100	810000	5E+06	8E+06	3E+06	2197600	1531100	718550	220060	1029500	668740	427120	480710	813470	823190	740290	748770	591060
MEDN632	N-Oleoyl Glyc	Organic Acid A	11639.83	10544	10992	8784.1	9868.9	9879.6	12725	12380	12637	13082	13762	10284	9236.7	13322	9827.4	10892	8239.8	12140	10407	8767.3	13191	9136.9	11586	11135	10484	13965	9670.8
MEDN636	Ginkgoic acid	Organic Acid A	65936.63	50065	59462	62101	87566	34101	61800	69024	50274	98164	64463	52256	70176	9	59102	41928	41415	49169	86461	78820	55680	45752	82557	84471	9	80701	47709
MEDN641	Ribose 1-phos	Carbohydrate r	499680	535350	12058	757950	2116500	53598	83190	117870	421350	354000	92982	842910	2E+06	1E+06	322550	9	9	258560	91259	9	9	9	96401	9	9	9	9
MEDN647	Hydroquinone	Phenols And Its	2174813	2E+06	2E+06	3E+06	2421400	2509000	1823200	1788600	2507800	1787300	2E+06	2E+06	3E+06	2E+06	1511200	2491900	1626600	1989300	2461400	4339300	1255300	1788000	1485200	2490900	2E+06	1463700	1882800
MEDN649	Anthranilic aci	Benzene and su	732732.5	807106	802615	783390	704510	806680	802010	590630	840120	564210	770310	884270	987230	750920	771540	686090	567710	913530	895560	1161600	481630	679260	540400	961880	911740	783660	900750
MEDN651	Succinic anhyd	Organic Acid A	595463.8	468152	515961	676610	191680	1448700	747530	249950	753420	255090	440730	619650	740650	242750	848070	590440	9	442840	260810	1501900	146960	364290	177570	540700	428650	259570	708050
MEDN655	Hydantoin-5-	Organic Acid A	20092.38	32554	35121	30831	14092	34133	22090	10761	16261	18683	13888	40243	48527	38832	11276	29619	22781	32228	36926	149340	9406.1	23621	16769	17709	22959	17392	23771
MEDN656	Indoleacetalde	Indole And Its I	1070074	560979	519529	954770	1380800	1126600	1051800	965340	1020800	1333900	726580	477070	499580	644410	392320	606030	690840	657190	520390	313870	482750	591360	469050	553730	658060	531640	555770
MEDN658	Hexadecanedi	Lipids_Fatty Aci	1012394	927914	799596	2E+06	791390	1535900	795700	684770	1294600	653700	750990	833600	744780	596550	1205100	1192700	998450	1261300	590830	1017900	290230	1002600	659660	980060	983030	478950	984340
MEDN661	Vanillylmande	Benzene and su	19583.88	18477	17157	18493	18221	18114	26512	17718	19844	15700	22069	20543	20683	18492	18778	19718	16378	18903	14317	13890	17779	15547	19545	17224	15147	19047	19079
MEDN662	Gamma-Glu-l	Organic Acid A	1329401	2E+06	2E+06	1E+06	1538900	1047200	1274400	1465800	1267200	1948800	706910	1E+06	1E+06	1E+06	1395100	2629900	1501200	1553000	1628600	3440000	1202100	1336800	2746200	2383200	1E+06	1248000	962840
MEDN666	Sulfoacetic aci	Organic Acid A	2014438	2E+06	2E+06	3E+06	2555000	2195200	1922600	1278300	2081200	1911400	1E+06	3E+06	3E+06	2E+06	1768500	2244700	2169900	1690800	1746800	2055500	695530	2371300	1106000	1902700	2E+06	1539100	1605200
MEDN667	PE(18:1(9Z)/0:	Lipids_Fatty Aci	20698750	2E+07	1E+07	3E+07	35596000	14480000	1.3E+07	1.1E+07	1.5E+07	3.2E+07	1E+07	1E+07	3E+07	3E+07	2.5E+07	1.9E+07	5004300	4674700	50815000	26932000	5148900	14205000	11457000	11608000	1E+07	7718800	13120000
MEDN670	O-Desmethylr	Benzene and su	42744.63	41368	35276	39784	51575	46913	24641	40075	51017	66119	21833	36402	45684	50989	48958	44098	25971	15969	62876	34953	42680	32308	52421	25487	31303	40873	22179
MEDN672	Adrenochrom	Indole And Its I	1395388	337207	9	610120	9	7336900	9	1650700	9	652320	913040	9	9	682250	9	770950	9	932470	311950	9	9	9	9	9	9	9	9
MEDN679	Maltol	Heterocyclic co	25675.13	31659	28384	19484	36927	24698	22705	25608	23290	34204	18485	37017	52594	30534	18002	31055	10765	41856	31446	37428	34766	30689	35929	27132	20075	24062	16989
MEDN681	1-Nonanal	Aldehyde	1.48E+08	1E+08	1E+08	2E+08	1.07E+08	177070000	1.8E+08	1E+08	1.6E+08	1.3E+08	2E+08	1E+08	8E+07	9E+07	1.7E+08	1.1E+08	131420000	1.76E+08	92045000	1.56E+08	76272000	142820000	1.08E+08	162600000	2E+08	130720000	1.71E+08
MEDN682	Octadecanam	Fatty acyls	16074.5	11427	15935	11660	13065	19586	18658	10865	26620	12787	15355	11445	12912	10129	13592	11523	9234.2	7331	15246	31190	24070	18198	14117	12621	9	7997.1	19281
MEDN689	2,5-Furandica	Organic Acid A	1252043	1E+06	1E+06	1E+06	1414900	1213800	996140	1506800	1272000	1373500	1E+06	1E+06	2E+06	1E+06	1551700	1300200	1340200	721240	1082300	1073500	1180500	1732300	1367200	997250	1E+06	1367400	1387000
MEDN691	Maltitol	Carbohydrate r	62085.25	62008	49004	68288	70872	82925	26503	55572	75659	66877	49986	60132	68760	58013	54964	104150	52353	26818	70872	63881	36586	45765	42855	76929	41639	44849	39526
MEDN693	2,5-Dimethylf	Heterocyclic co	43894.63	45744	44865	46919	55914	43957	25229	46205	50604	47564	34765	57584	56438	36594	40832	47230	49545	28316	49416	44600	43078	47169	50733	35481	49590	36457	51812
MEDN694	2-nonanol	Alcohol	434975	365538	412083	468710	294800	508660	522360	301730	476030	385790	521720	383560	261420	258230	539010	342870	391630	494250	253330	442440	202660	418250	337850	504430	481320	401770	507940
MEDN704	Oxaloacetic ac	Organic Acid A	69798.25	52900	56842	102500	77043	74630	62948	31530	54330	58215	97190	56013	53459	39086	56790	45486	45868	97382	29115	77748	24198	20928	26485	45226	91519	41152	127480
MEDN706	ButylAcetate	Fatty acyls	127302.3	114246	146387	209820	153400	117340	245290	139830	9	9	152720	113450	9	123540	147860	100240	128560	300300	9	136440	132290	166550	140770	205440	147020	91139	151450
MEDN710	LactobionicAc	Carbohydrate r	315508.8	229170	259006	376270	345970	218760	375090	281910	364990	305000	256080	206430	198410	300260	193630	212610	168780	156890	396350	373510	285750	157490	190990	411630	206430	191520	254730
MEDN714	MethylLinolen	Fatty acyls	43224.38	44764	41082	41888	46060	37388	41872	43827	42572	46970	45218	43364	46320	47049	44901	44576	46100	42860	42942	38159	48006	33989	46802	44063	40112	39193	38330
MEDN720	N-(2-Methylb	Amino Acid m	24939.75	30085	28395	29363	35993	9	20131	24693	23947	47745	17637	28422	30126	33237	22229	27336	35617	29675	34039	28837	20404	40051	24878	33223	25365	18363	36037
MEDN722	Orcinol	Phenols And Its	43082.13	93139	34141	52708	92725	36138	39119	18158	48022	32983	24804	136170	224310	104160	55345	84021	34778	72089	34242	34682	20693	35803	23148	49602	31448	35491	42257
MEDN725	Sorbic acid	Organic Acid A	876407.5	775986	773346	702890	872350	1417100	891860	857980	925800	880470	462810	1E+06	868780	620400	822450	649210	660300	577100	952450	674450	764840	1098500	866630	679490	607050	753520	742290

MEDN726	Decanal	Aldehyde	1251449	1E+06	1E+06	1E+06	1352500	1172000	926090	1450400	1315900	1351600	1E+06	2E+06	1E+06	1E+06	1237300	1366900	1374300	789720	994150	1039400	1070900	1706200	1562700	939640	1E+06	1483900	1329600
MEDN727	Methyl hexad	Fatty acyls	98191.38	102980	98915	103790	97022	90179	93118	95072	103000	103020	100330	104400	102070	89130	102160	120800	97713	95014	112550	86178	104330	84865	109370	92871	108360	111770	93572
MEDN731	4-tert-Octylpl	Phenols And Its	244150	251074	393930	206560	300820	188730	283760	253440	216360	269450	234080	221710	250090	240630	209800	249040	218110	232780	386430	519050	654810	157250	219800	357840	263570	250730	728390
MEDN732	RICINOLEIC A	Fatty acyls	259348.8	303494	272859	159630	148960	314810	413390	271550	150020	130950	485480	293820	201120	273850	221220	235640	509310	559400	133590	404870	183640	351040	126380	264610	330910	202580	318840
MEDN733	PHENYL-BETA	Carbohydrate r	120393.8	103680	146555	91472	57928	121160	103410	130530	152420	97660	208570	114190	75281	78671	114260	144450	99599	128590	74400	171490	96232	124670	95193	94377	201440	160860	228180
MEDN734	Cis-3-Hexeny	Fatty acyls	1.51E+08	1E+08	1E+08	2E+08	1.16E+08	171930000	1.8E+08	1.1E+08	1.6E+08	1.3E+08	2E+08	1E+08	9E+07	9E+07	1.8E+08	1.1E+08	136600000	1.78E+08	94249000	1.64E+08	80184000	151340000	1.15E+08	164240000	2E+08	138110000	1.7E+08
MEDN735	Pyridoxaminel	Amines	1933988	2E+06	1E+06	2E+06	1697300	1886000	2288200	1762300	1845200	1958500	2E+06	2E+06	2E+06	2E+06	1549900	1619100	1475800	2160800	1914400	1171300	1402100	1474100	1068800	1419800	1E+06	1778000	1774900
MEDN736	2-(Methylthio	Alcohol	205610	178938	148515	203140	175820	226110	192120	215480	187580	218180	226450	164960	190580	191690	139220	186830	174810	212570	170840	104550	139270	135460	111700	173750	143520	180300	199570
MEDN737	DocusateSodi	Organic Acid A	30917.63	24715	47154	53217	17509	38054	34052	25195	27329	20458	31527	15618	9	20368	58601	14226	30957	42509	15431	31402	20483	75643	24395	48055	61491	60850	54916
MEDN738	2,6-Dimethyln	Benzene and su	1119410	1E+06	1E+06	1E+06	1010200	1176900	1136000	1149900	1125600	1175300	976680	2E+06	1E+06	1E+06	1321500	1244000	1280200	656790	965160	872470	824340	1342200	1207200	931470	1E+06	1152700	1164900
MEDN739	4-Hydroxyber	Benzene and su	67428	90831	56882	67003	84885	56030	68509	58613	76309	81138	46937	89044	131460	93540	69627	97729	89574	98221	57450	81847	43653	51969	57910	54112	52584	51771	61210
MEDN741	Methanesulfo	Others	896090	853195	780798	1E+06	869390	1220600	1165100	670940	882930	666720	594740	1E+06	657100	459060	637510	548080	463430	1921200	834080	761020	430110	1314600	439740	776240	1E+06	488330	943740
MEDN743	2-Nonanone	Ketones	1.68E+08	1E+08	2E+08	#####	1.25E+08	191560000	2E+08	1.2E+08	1.8E+08	1.4E+08	2E+08	2E+08	1E+08	1E+08	2E+08	1.2E+08	151250000	2.05E+08	103950000	1.78E+08	90071000	159440000	1.32E+08	186500000	2E+08	153760000	1.97E+08
MEDN744	delta-Hexalac	Lactone	246787.5	241380	228440	221120	259820	247600	230240	242900	238780	280790	253050	284040	239890	246540	253430	239960	282060	193300	191820	235750	215510	284070	218920	216370	230250	190890	235760
MEDN747	2,4-Di-tert-bu	Phenols And Its	21890.63	22968	34312	14074	27879	20976	27609	23455	19398	22192	19542	19862	26509	24841	20928	21364	19701	16733	33806	43469	59964	14971	20644	28348	26983	20025	60090
MEDN748	Methylparabe	Benzoic Acid A	526127.5	714403	795801	612680	491130	610320	372780	514570	512310	578380	516850	585820	633250	574590	569170	723820	802020	998660	827890	1098700	565480	676420	831630	780600	802140	835300	776140
MEDN750	(±)12-HEPE [(	Oxidized lipid	9739.813	11242	9288.2	7630	8407.2	9723.1	11868	14480	4820	7739.2	13251	12784	7334.7	11330	10830	9706.3	16273	13843	7833.9	5967.7	7497.1	13637	6185.4	11299	12652	7621.4	9445.9
MEDN751	(±)12-HETE [(	Oxidized lipid	11860.65	10284	8613.4	7054.5	7619	13904	7084.3	24551	11825	13838	9009.4	13980	9508.5	11422	6862.8	10056	12520	11710	6214.9	8211.9	9	19095	6736.1	6797.5	8716.8	8870.2	10471
MEDN754	(±)17-HDHA [	Oxidized lipid	8177.838	9998.3	5771.8	7339.2	6501.8	7374.1	6790.7	14405	5193.8	6099.1	11719	11111	6824.1	10661	9340	6814.8	18580	12526	4129.8	2857.8	2830.9	8170.4	5011.4	5517.2	9680.7	5369.2	6736.4
MEDN756	(±)18-HEPE [(	Oxidized lipid	23061.13	50569	38199	27960	34325	38667	19179	35386	9	10045	18918	48066	56484	51470	49974	33040	89156	58539	17823	23759	24136	40886	72304	31439	76971	16762	19332
MEDN757	(±)18-HETE [(	Oxidized lipid	4334.75	4593.7	3475	4451.5	7146	3648.6	2466.8	4814.3	3636.7	5771.7	2742.6	2162.6	5843.4	7157.9	2700.5	5460.8	3588.5	2587.6	7248.1	3770.2	3666.3	4044.8	3221.2	3519.5	2715.5	3750.6	3111.7
MEDN758	(±)4-HDHA [(	Oxidized lipid	30571.75	37177	31040	23917	23434	40814	28001	40537	26736	20697	40438	55493	28189	27257	21925	27971	70321	51060	15197	20572	17192	35764	18827	39062	55582	28035	33287
MEDN759	(±)5-HEPE [(±	Oxidized lipid	14070.59	16822	11844	11215	8423.7	15718	12524	23009	10799	10728	20148	27763	13152	15719	12000	11754	27910	18657	7618.5	10051	7359.3	18873	4928.6	12000	16886	12094	12562
MEDN760	(±)5-HETE [(±	Oxidized lipid	18292.25	20613	17570	15082	12549	25947	16276	21557	21367	12206	21354	30144	16675	15903	13215	18168	36003	23011	11787	13091	9076.1	31726	13810	15458	22686	18562	16147
MEDN761	(±)7-HDHA [(	Oxidized lipid	12146.48	17281	13088	7619.3	8968.6	12880	14541	14657	10866	8934.9	18705	21906	9551.5	13064	7385.4	12065	43221	25399	5659.8	8337.8	6838.8	22691	6809.9	12962	19685	9578.8	17801
MEDN764	11(S)-HETE [1	Oxidized lipid	11493.65	12082	9127.2	8107.7	7081.1	11494	10087	17339	14888	8550.4	14402	17099	8330.4	6423.3	10808	9576.9	18468	20306	5641	5612.6	9	13740	5829.5	11508	16607	11929	7782.6
MEDN765	11,12-EET [(±	Oxidized lipid	13522.43	14954	12230	10036	9134.9	12903	13660	17310	12169	9641.5	23325	16264	9245.5	7952.3	9705.1	17317	26287	25891	6973.7	8420.6	3918	19503	8135.8	16528	17449	10064	13819
MEDN767	12,13-EpOME	Oxidized lipid	4735213	5E+06	5E+06	3E+06	2752200	5083900	7146600	4242700	3235600	2704600	1E+07	7E+06	3E+06	5E+06	3732900	4208100	7825700	8384000	2527500	9768000	3922100	6663900	2249500	5243800	7E+06	3247500	5222000
MEDN768	13-oxoODE [1	Oxidized lipid	418252.5	413431	425523	320330	161660	418930	534010	838940	242540	209680	619930	425370	227940	327340	356510	325550	649220	827310	168210	364100	199460	957660	136080	383980	411740	358040	593120
MEDN769	14(S)-HDHA [	Oxidized lipid	14948.25	18524	13372	11916	11030	16440	14197	26251	12038	10546	17168	25023	12573	22003	12317	15406	36196	17445	7227.7	6858.9	8483.8	17109	8114.8	16332	17170	13847	19059
MEDN770	14,15-EET [(±	Oxidized lipid	23105.5	23896	24218	16394	16706	30810	24390	26081	21809	15785	32869	37148	19920	16461	16754	35040	16493	30035	19313	23620	12161	25792	17184	35065	30200	23092	26633
MEDN771	15-oxoETE [1!	Oxidized lipid	10371.08	9707.2	10720	9803.7	6931.7	11323	6563.1	20901	11757	6303.4	9385.7	10432	5610.3	9274.8	7926.4	5618.2	16177	16717	5901.6	7326.3	7563.5	21221	8439.3	12831	10695	8648.3	9038.6
MEDN772	16(17)-EpDPE	Oxidized lipid	33211.13	36945	29534	27206	34927	24330	39670	34650	19366	26543	58997	40087	32042	46881	20386	39823	64411	32496	19430	24817	32686	37897	21240	27630	39244	25505	27254
MEDN773	17(18)-EpETE	Oxidized lipid	57052.75	58492	52461	39022	59614	66155	66294	56062	45270	37362	86643	84041	44672	60692	40359	70819	51003	63896	52456	40375	33605	49952	30835	78930	69607	53778	62606
MEDN777	5,6-DiHETrE [	Oxidized lipid	6059.938	7631.6	6135.9	3691.2	6723.6	7695.5	4525.1	5772	6722.5	6592.1	6757.5	8755.8	7289.6	7952.5	5341.2	7732.3	12288	7635.1	4058.2	3787.7	5368.9	10532	8912.9	5774.7	6572.7	4537.7	3600.4
MEDN778	5,6-EET [(±)5,(	Oxidized lipid	3540.825	1795.1	2053.6	4061.2	2922.7	3720.4	2635.7	4327.8	3489.7	3699.4	3469.7	9	9	2961.2	2013.5	3421.1	2433.9	3504.1	9	2143.8	9	2219.3	2424.5	3301.6	3109.5	3212.1	9
MEDN783	9,10-EpOME	Oxidized lipid	2096205	2E+06	2E+06	1E+06	1057300	2815600	2773900	2034300	1459500	976840	4E+06	2E+06	2E+06	2E+06	1680200	1982300	4064500	4668200	948940	3460000	1756700	2745900	711150	1974100	3E+06	1525900	2251800
MEDN784	9-oxoODE [9-	Oxidized lipid	579820	519248	399378	279050	295310	376490	983520	956180	395760	277550	1E+06	725270	244740	375070	338100	576010	1002200	715450	177140	307850	230210	770050	109730	336620	605790	301120	533650
MEDN793	PGE2 [9-oxo-	Oxidized lipid	75584.13	130263	18574	26650	76879	249430	112530	50002	10046	26687	52449	440730	50724	13145	116360	20262	67976	332900	9	21498	15941	16573	14097	18666	18011	13216	30591
MEDN798	PGK1 [9,11-di	Oxidized lipid	43660.13	44778	42938	29548	29579	42279	74768	37051	46110	33909	56037	44394	41632	50764	38959	16062	48944	79159	38307	49205	33271	24234	30730	48605	62800	34194	60465
MEDP006	Glycine	Amino Acid m	152685.5	164144	254753	92635	162270	53010	208160	245200	83629	185340	191240	142610	152540	204470	131850	200130	158470	144070	179010	130780	525310	221000	501120	136680	166030	162610	194490
MEDP007	L-Cystine	Amino Acid m	197115	140515	177738	261310	232520	234570	143680	160960	183480	210440	149960	175910	105160	91508	157470	176050	167960	166980	83085	198820	153140	141460	163130	142980	178050	140820	303500
MEDP008	L-Threonine	Amino Acid m	3579400	4E+06	5E+06	3E+06	3606800	2738800	5009900	4372400	2552400	3571900	4E+06	4E+06	3E+06	5E+06	3494600	2848000	4656700	5397300	4809000	3691800	7889500	4044000	5513000	4153600	4E+06	4606400	4412100
MEDP009	L-Tyrosine	Amino Acid m	28760000	3E+07	3E+07	3E+07	32726000	38517000	2.7E+07	2.7E+07	1.8E+07	3.1E+07	3E+07	2E+07	3E+07	3E+07	2.9E+07	3.9E+07	39509000	56583000	28856000	25699000	22554000	30987000	21646000	33681000	3E+07	26398000	31672000
MEDP011	L-Lysine	Amino Acid m	13717013	2E+07	2E+07	1E+07	22625000	11108000	9764000	1.5E+07	1.2E+07	2.3E+07	6E+06	6E+06	2E+07	2E+07	6795500	2.5E+07	13332000	9331700	21001000	16004000	36768000	12831000	25358000	13672000	9E+06	12470000	7919100
MEDP012	L-Ornithine	Amino Acid m	40037.85	45597	199101	35451	35217	26795	7999.8	40751	36621	120170	17298	30655	48506	46721	29041	86192	16159	23852	83652	387860	297090	77441	636170	120110	26492	36359	11287
MEDP013	L-Alanine	Amino Acid m	11136175	1E+07	1E+07	1E+07	11083000	13042000	1.2E+07	1.2E+07	8008000	9302400	1E+07	1E+07	1E+07	1E+07	1.2E+07	1E+07	11384000	15969000	12302000	6775500	16334000	9700800	12360000	10383000	9E+06	11577000	8703700
MEDP017	L-Histidine	Amino Acid m	1264734	1E+06	2E+06	940780	1256800	1518000	1363200	1349800	656590	1911500	1E+06	924610	794010	2E+06	929730	1012500	819880	1594700	2042800	1929800	3283700	2694200	1649600	1410400	1E+06	1466200	981920
MEDP018	L-Isoleucine	Amino Acid m	76435250	9E+07	7E+07	6E+07	71272000	99732000	7.7E+07	7.9E+07	5.6E+07	7.3E+07	9E+07	8E+07	7E+07	1E+08	6.4E+07	8.4E+07	115450000	1.8E+08	44881000	72528000	40065000	61121000	49698000	101270000	9E+07	48783000	1.01E+08
MEDP019	L-Leucine	Amino Acid m	78236250	9E+07	7E+07	8E+07	70131000	100160000	8.2E+07	7.9E+07	5.6E+07	7.2E+07	9E+07	8E+07	7E+07	1E+08	6.4E+07	8.5E+07	130050000	1.77E+08	44294000	68767000	44394000	63616000	49300000	94715000	9E+07	49645000	1.03E+08
MEDP020	L-Methionine	Amino Acid m	11886263	1E+07	9E+06	1E+07	12107000	8876200	8366500	1.4E+07	6724400	1.2E+07	2E+07	7E+06	1E+07	2E+07	7947000	1.5E+07	10136000	11724000	12697000	10396000	8110500	9326400	6890900	10783000	7E+06	8757700	8289800
MEDP022	L-Proline	Amino Acid m	1.11E+08	1E+08	1E+08	1E+08	1.29E+08	113740000	1.1E+08	1.1E+08	9.4E+07	1.3E+08	1E+08	1E+08	1E+08	1E+08	8.9E+07	1.3E+08	150160000	96308000	123330000	1.46E+08	136360000	102670000	1.31E+08	127540000	1E+08	122210000	99007000
MEDP026	L-Valine	Amino Acid m	24046750	3E+07	2E+07	3E+07	28627000	25089000	1.7E+07	2.4E+07	2.7E+07	2.3E+07	2E+07	3E+07	3E+07	3E+07	2.4E+07	3.1E+07	27362000	19858000	26234000	24542000	22365000	20358000	21730000	23745000	2E+07	32454000	23149000
MEDP031	3-Chloro-L-Ty	Amino Acid m	65665.75	49358	53018	95237	70237	50009	66766	76619	62766	51154	52538	39054	34710	47595	52830	32145	59820	49400	79312	54631	44876	28800	53536	49697	46839	74466	71296
MEDP032	3-Hydroxy-3-	Amino Acid m	3757175	4E+06	4E+06	3E+06	4322400	3421800	2669400	4355300	3788000	4918700	3E+06	5E+06	5E+06	4E+06	3085100	3824300	4610700	3541900	4276700	3853100	3654600	4091900	4364000	3599000	3E+06	3749200	2987300
MEDP033	3-Iodo-L-Tyro	Amino Acid m	42356.88	115092	195879	9	9	177280	94804	9	9	9	66726	133050	89805	109730	117030	9	82991	117890	270230	299430	224360	246420	235090	164060	158740	142890	96042
MEDP034	3-N-Methyl-L	Amino Acid m	242856.3	325496	304921	191790	276190	131650	200500	293250	209720	438320	201430	226260	554350	404060	195850	379810	251210	233720	358710	339620	514930	261040	426730	210160	194440	248800	243650
MEDP036	5-Oxoproline	Amino Acid m	12577625	1E+07	1E+07	1E+07	13446000	10661000	1.1E+07	1.6E+07	1.2E+07	1.5E+07	1E+07	1E+07	1E+07	1E+07	1.2E+07	1.4E+07	10718000	10532000	15312000	9784000	16629000	15115000	14633000	11853000	1E+07	14329000	10760000
MEDP037	Asp-Phe	Amino Acid m	463223.8	398366	439298	637260	562710	330050	322290	451030	444990	574090	383370	301690	474290	543780	287020	444580	339950	392590	403190	660810	413710	556410	361780	380290	359540	367030	454810
MEDP038	Beta-Alanine	Amino Acid m	1746475	2E+06	3E+06	2E+06	1520000	1821100	1647500	1786200	1832200	1668500	2E+06	2E+06	2E+06	2E+06	2417200	2282700	2092600	2361000	2353100	2624600	2174000	2854000	2659300	2612300	3E+06	3057400	2964600
MEDP039	Betaine	Alkaloid	1.27E+08	1E+08	1E+08	1E+08	1.37E+08	131120000	1E+08	1.3E+08	1.5E+08	1.3E+08	1E+08	1E+08	2E+08	#####	1.2E+08	1.4E+08	132500000	89522000	139460000	1.22E+08	108660000	112780000	1.15E+08	132700000	1E+08	138960000	1.29E+08
MEDP043	Glutathione O	Amino Acid m	28430.25	21076	19711	30103	32400	18530	17804	33669	41181	38373	15382	8031.8	33630	33040	23364	32107	16588	11620	10227	25539	11801	33905	11493	23728	18818	22087	10316
MEDP044	Glutathione R	Amino Acid m	76229.75	87324	68091	100420	76908	67202	90641	77215	67517	85975	43960	92127	104880	80310	57141	112250	78738	33399	139750	80644	95304	99238	99675	49462	65487	13934	40981
MEDP045	Glycyl-L-Proli	Amino Acid m	137476.8	220573	208768	128510	190020	75568	90476	169500	160230	173410	112100	222660	203300	235440	138520	203050	323520	162350	275740	375250	284130	157680	207040	200190	181590	79776	184490
MEDP047	Histamine	Polyamine	3738225	4E+06	2E+06	3E+06	3442300	4627000	3872900	5136900	2923900	3134300	4E+06	4E+06	3E+06	4E+06	2468600	2425900	3248400	7751400	3007900	1868100	1218900	2175200	1644000	2148800	3E+06	2231000	4202700
MEDP053	L-Dopa	Amino Acid m	624823.8	739000	653575	637760	727000	820330	613400	577980	496190	640430	485500	643270	877020	671590	646570	706940	659570	614240	1092800	646990	700930	650050	534010	786310	548980	767390	593940
MEDP054	L-Glutamine	Amino Acid m	13419938	2E+07	2E+07	1E+07	18944000	10232000	9095100	1.5E+07	1.2E+07	2.5E+07	6E+06	7E+06	2E+07	2E+07	6729100	2.2E+07	13415000	9180500	22904000	15673000	36154000	13588000	24441000	13594000	1E+07	12977000	8318500
MEDP056	L-Homocystin	Amino Acid m	412305	569635	684106	435120	397720	616740	329230	433250	375760	511390	199230	481880	584360	611400	569270	789760	559480	332680	628250	613450	895520	439360	1259900	541060	604340	627330	491890
MEDP059	L-Theanine	Amino Acid m	6768200	7E+06	7E+06	6E+06	10418000	5961200	4776000	6174100	5758900	1E+07	4E+06	6E+06	9E+06	8E+06	4664700	7558300	6640200	5921200	10261000	5465200	11309000	6829600	8632600	5781300	5E+06	7546000	4879700
MEDP060	Methionine Su	Amino Acid m	3981738	5E+06	4E+06	4E+06	6275200	2281500	3910900	4077300	3751600	5268600	2E+06	5E+06	7E+06	6E+06	2198400	5121300	5418600	4264400	5430500	4308200	5007400	4352400	4764200	4418800	3E+06	3892000	2939300
MEDP061	N,N-Dimethyl	Amino Acid m	2112588	3E+06	2E+06	2E+06	1807200	2276300	1892200	3216700	1741800	2201800	2E+06	2E+06	3E+06	4E+06	2218400	3112600	2572900	1504000	2055400	1980400	1894900	2924900	2495100	2788700	2E+06	3067600	1925500
MEDP063	N-Acetylaspa	Amino Acid m	900796.3	1E+06	1E+06	741010	885030	694850	1070500	989350	665560	1294300	865770	681250	490910	1E+06	882150	1106800	1008000	375390	2763000	1003700	1703600	1324500	854290	1292400	608710	807160	1180900
MEDP065	N-Acetyl-L-G	Amino Acid m	8390063	8E+06	8E+06	9E+06	11275000	6929400	6330400	8435400	7416300	1.2E+07	6E+06	8E+06	9E+06	1E+07	5768400	9476600	6733900	4249500	10973000	7883300	8879500	7891700	9690300	7651200	7E+06	6894200	6040800
MEDP067	N-Acetylmanr	Amino Acid m	435215	450045	460415	386110	493850	376630	287610	608700	225420	538470	564930	360520	398550	580940	493890	418590	437330	432220	478320	344660	538540	505380	550980	583650	366760	357940	435410
MEDP069	N-Acetylputre	Organic Acid A	226168.8	215278	232800	197850	178700	221840	255750	253100	235840	194160	272110	270910	256990	228090	182790	207280	194300	185420	196440	220060	230160	323410	194300	187470	163180	267370	276450

MEDP070	Ng,Ng-Dimet	Amino Acid m	6369725	8E+06	7E+06	6E+06	12205000	4705200	4732500	6168400	4574600	8768600	4E+06	6E+06	1E+07	1E+07	4185700	8449400	6576200	4105700	10173000	8132700	12333000	5534600	9531300	5928700	4E+06	6701400	4014000
MEDP073	N-Phenylacet	Amino Acid m	2817020	2E+06	251226	681070	342940	14396000	479650	3734100	334390	905010	2E+06	523950	487820	3E+06	255980	5123700	395760	7263300	553630	450300	92466	436860	113280	267380	217590	169700	262230
MEDP078	Phe-Phe	Amino Acid m	152555.3	154435	131203	170230	241530	56021	43245	256540	131840	275240	45796	121070	189650	320740	103150	157020	157300	56999	129550	100280	262060	147260	169460	69934	79822	134920	85886
MEDP079	S-(5-Adenosy	Amino Acid m	19447.75	20493	22479	10137	24762	22169	14335	17267	25616	22811	18485	28458	19686	30274	15839	26929	15566	4829.9	22360	29315	20943	28799	27342	20472	15731	16999	20233
MEDP080	S-Adenosyl-L	Amino Acid m	79772.38	97613	84451	76309	154590	56610	68675	67326	27273	139980	47416	72351	118140	174370	52432	53579	53056	77343	179630	141260	134050	62570	142330	72154	36183	70883	16180
MEDP082	S-Sulfo-L-Cys	Amino Acid m	315930	310305	308560	362780	332820	289740	239830	329070	273540	457200	242460	372990	302110	335940	255000	300140	421450	135010	359800	249990	363410	347230	443860	286910	232210	320870	224000
MEDP083	Trans-4-Hydr	Amino Acid m	14261875	1E+07	1E+07	1E+07	13888000	14161000	1.3E+07	1.7E+07	1.2E+07	1.5E+07	1E+07	2E+07	2E+07	1E+07	1.3E+07	1.9E+07	14253000	9569800	18265000	8967800	16470000	11557000	14023000	12939000	1E+07	15593000	12049000
MEDP084	Trimethylamin	Amino Acid m	2055519	2E+06	62351	101320	25642	10156000	266810	3716400	37427	821050	1E+06	31686	14731	2E+06	92213	4190400	134910	10802000	88316	152820	77657	82177	26683	89181	12552	13085	44656
MEDP085	Tyramine	Benzene and su	474440.1	558261	170194	778840	1286800	144700	152000	258070	406220	678930	89961	540960	2E+06	604770	148560	450560	323250	172490	151400	173520	92515	318530	322040	109290	74188	148740	122730
MEDP086	Urea	Amino Acid m	4615763	5E+06	5E+06	4E+06	4738200	4896900	4473800	4388100	4682600	4574100	5E+06	5E+06	5E+06	5E+06	4692800	4484800	4905200	4793700	4343000	5030500	4789800	3883100	4735700	4686300	4E+06	4426000	4519100
MEDP087	L-Alanyl-L-Ly	Amino Acid m	269855	246314	269543	144660	379400	348780	217380	220060	329810	269800	248950	222690	229650	233650	226170	293070	239130	190760	335390	370900	216000	274900	193420	193580	261600	369710	276230
MEDP089	N-Acetylhistai	Organic Acid A	3250825	3E+06	3E+06	6E+06	5109900	3486800	3555600	1188100	1880100	3049800	2E+06	1E+06	4E+06	4E+06	2555300	3358300	2954200	2151000	2930200	4278800	2301300	1154100	2460800	5050600	2E+06	2319900	2430900
MEDP102	Syringic Acid	Benzene and su	466075	457118	431068	554840	533480	424240	392370	448410	466630	579170	329460	442310	515720	553770	311510	524260	547530	271940	489900	439630	383700	536470	479880	410390	350220	425490	422760
MEDP107	Methyl Benzo	Benzene and su	2497750	3E+06	3E+06	3E+06	2440600	3178500	2840900	1530000	2511500	1636200	3E+06	4E+06	4E+06	3E+06	2907300	2142700	1965400	3577400	3435400	4759000	1714400	2132400	1758700	3802400	3E+06	2568000	3247800
MEDP111	3-(4-Hydroxy	Benzene and su	578516.3	794156	362510	650300	601470	879310	505100	520780	611510	434610	425050	1E+06	1E+06	884770	549540	681720	431440	992520	358560	438790	215550	313490	272920	445900	436340	327730	449360
MEDP113	4-Hydroxyber	Benzene and su	2601921	2E+06	3E+06	2E+06	2656600	3524000	3808400	1852100	1790800	4224900	608170	2E+06	4E+06	3E+06	1411100	2208500	2846800	2185200	1187800	4301600	1696400	1840400	2627200	3115200	3E+06	2694700	2785800
MEDP114	Phthalic Acid	Benzene and su	1868275	2E+06	2E+06	2E+06	1904700	2175500	2012300	1803100	1229900	1691300	2E+06	2E+06	2E+06	2E+06	1721500	1945000	2704800	3976900	1262000	1417000	851170	1197200	1012000	2267900	2E+06	1353400	2246200
MEDP115	1,4-Dihydro-1	Pyridine And Py	68014125	6E+07	6E+07	1E+08	1.01E+08	76072000	7.3E+07	2.6E+07	4.2E+07	7.2E+07	5E+07	3E+07	8E+07	7E+07	5E+07	6.9E+07	49864000	41855000	63227000	84254000	50353000	27250000	54268000	101210000	5E+07	46234000	48252000
MEDP117	3-Carbamyl-1	Pyridine And Py	1402101	2E+06	172904	1E+06	4802400	487600	434000	661400	1725200	1229900	426710	4E+06	7E+06	2E+06	689410	1680800	381020	1356500	238120	114190	200220	97210	401490	158840	104020	201590	105670
MEDP118	4-Pyridoxic Ac	Pyridine And Py	18337375	3E+07	9E+06	2E+07	26519000	16324000	1.5E+07	1.1E+07	2.8E+07	1.3E+07	1E+07	4E+07	7E+07	3E+07	1.8E+07	2.9E+07	15079000	17492000	12867000	15860000	3606500	11625000	4884000	12615000	9E+06	7004300	11037000
MEDP119	6-Hydroxynic	Pyridine And Py	446160	428306	410303	428690	396990	602010	534580	367220	339870	601900	298020	445250	698130	618020	187210	474630	428050	330210	244950	536240	259270	315480	503100	480070	389440	424820	374000
MEDP122	Picolinamide	Pyridine And Py	512631.3	459479	2E+06	173020	554800	550630	559740	1056700	252750	314000	639410	193200	893580	436440	378790	250380	371040	373240	779160	209270	236130	11696000	138540	164370	306940	245240	253540
MEDP123	Acetylcholine	Cholines	3669075	4E+06	2E+06	4E+06	4792000	4036900	2255100	3660700	2840000	4158400	3E+06	5E+06	5E+06	3E+06	4437200	3897900	2781500	3237200	6661000	2192500	2846700	1740400	3210100	2188500	2E+06	3153900	2167600
MEDP125	Choline	Cholines	672488.8	816928	771634	545970	563350	931800	681350	936210	481630	666410	573190	710920	834970	1E+06	725740	1059400	724740	607040	607810	625640	698980	747960	885860	918210	726230	923300	646890
MEDP126	1,5-Diaminop	Polyamine	1579093	2E+06	1E+06	2E+06	2341500	479040	1259900	2322900	1649300	1865500	645000	953570	3E+06	2E+06	658900	2612200	1636700	2087100	2091300	1584000	1136900	971140	940200	1199400	1E+06	1125600	1547000
MEDP127	Putrescine	Polyamine	360387.5	347758	361586	329000	274670	369980	393470	360440	379270	371270	405000	355350	326280	339030	309120	377680	342480	332330	399790	362810	368830	421660	329240	308730	424210	254140	423070
MEDP128	Diethanolamir	Polyamine	147423.9	147082	157550	61027	152500	111900	145700	168410	99671	91593	348590	161860	137720	133910	187600	92356	121820	230830	110560	82557	199660	85085	137360	128650	313310	124510	189270
MEDP129	Phenethylamir	Polyamine	95021.5	112562	73151	132200	87998	122050	109900	79649	74965	59654	93756	107130	84321	89703	85115	101040	149460	214010	69719	87408	33644	73111	43561	83949	91035	67501	105000
MEDP130	4-Nitropheno	Phenols And Its	15976500	2E+07	1E+07	2E+07	16253000	17779000	1.2E+07	1.5E+07	1.7E+07	1.8E+07	2E+07	2E+07	2E+07	2E+07	1.3E+07	1.6E+07	15901000	13774000	17482000	16042000	16143000	14823000	17242000	14239000	1E+07	14879000	11473000
MEDP132	Dulcitol	Carbohydrate r	108244.5	115233	91647	75385	96266	80602	101460	159400	95543	148270	109030	101970	124750	121060	81067	159310	122230	91868	119610	42304	125250	120620	93173	86682	86352	111550	67248
MEDP134	Inositol	Carbohydrate r	45988750	4E+07	4E+07	5E+07	46440000	51223000	3.5E+07	4.5E+07	4.5E+07	5.4E+07	5E+07	5E+07	5E+07	4E+07	3.8E+07	4.7E+07	47806000	38708000	46758000	45935000	49356000	38336000	46786000	39148000	3E+07	40169000	31194000
MEDP135	Myoinositol	Carbohydrate r	39966125	4E+07	3E+07	4E+07	42330000	41789000	2.7E+07	4.1E+07	4.3E+07	4.8E+07	4E+07	4E+07	4E+07	4E+07	3.1E+07	4.1E+07	41364000	32255000	42023000	41589000	40002000	32158000	39165000	33832000	3E+07	35771000	26326000
MEDP137	Protocatechui	Phenols And Its	17050875	2E+07	2E+07	2E+07	16937000	19570000	1.9E+07	1.2E+07	1.9E+07	1.2E+07	2E+07	2E+07	2E+07	2E+07	1.9E+07	1.5E+07	12684000	22785000	19708000	26567000	10853000	14391000	12155000	22095000	2E+07	17046000	20689000
MEDP146	1-Methyladen	Nucleotide met	4949625	5E+06	5E+06	5E+06	5103000	5197000	4921000	4474000	4864600	5117900	5E+06	7E+06	7E+06	5E+06	4319200	4627800	5634600	4869200	3238200	6777300	4347800	4411600	7885000	4472300	5E+06	4026200	5126100
MEDP147	1-Methylhistic	Amino Acid m	910606.3	1E+06	762974	1E+06	1654600	737770	699110	856390	799600	925680	603300	2E+06	2E+06	1E+06	1129700	1400400	891070	632350	1023900	910940	632640	708310	910750	696380	768430	793430	682910
MEDP149	2'-Deoxyaden	Nucleotide met	7800020	6E+06	6E+06	9E+06	19015000	1189300	485060	1569300	1.1E+07	2E+07	1E+06	442150	1E+07	1E+07	6633600	6647900	9	9	6273200	23483000	11656000	1099000	4533700	2165400	340540	1906200	831400
MEDP150	2'-Deoxyinosi	Nucleotide met	4065613	5E+06	4E+06	6E+06	8866800	2091600	1724000	1502200	4495700	6513300	1E+06	1E+06	1E+07	8E+06	2855600	4850100	3061700	1043600	2247800	11637000	5354100	1250400	4820000	16282	2E+06	1390600	2970300
MEDP151	2-Hydroxy-6-	Nucleotide met	8354288	1E+07	2E+06	1E+07	10766000	12016000	7979900	3649100	8442700	6648500	7E+06	1E+07	2E+07	9E+06	1.1E+07	8967000	6517100	10835000	6009900	3895000	966250	3843300	1222600	1936400	3E+06	1787100	3107100
MEDP152	3'-Aenylic Aci	Nucleotide met	4041509	4E+06	6E+06	4E+06	2363600	1348800	807770	5464700	8290100	8045400	2E+06	893580	3E+06	3E+06	9572200	4842700	546100	417810	6063000	6675400	4356700	26342000	2601300	1059900	688990	2325000	989690
MEDP154	5,6-Dihydro-5	Nucleotide met	612751.3	699261	782994	709830	966390	880500	471950	407060	370190	549790	546300	518580	618420	665620	366980	1142400	746640	864060	671390	2171900	400170	602920	989040	622550	307160	463320	706890
MEDP156	5-Methyluridi	Nucleotide met	2354338	2E+06	878288	4E+06	4062600	1891700	1780800	1140100	1474300	3920000	845000	977760	3E+06	3E+06	1351000	3238000	1725400	993600	1645300	523430	778900	508150	430460	1303300	854060	1421900	1206100
MEDP157	7-Methylxantl	Nucleotide met	5827988	7E+06	4E+06	6E+06	8195200	5162200	3853100	5247000	6571800	7589100	4E+06	8E+06	1E+07	7E+06	5259100	7852500	5078000	4902400	4540700	5506600	3660000	3217000	5766500	3679000	3E+06	3777100	3635200
MEDP158	8-Hydroxygua	Nucleotide met	1182733	1E+06	1E+06	1E+06	1663900	1189400	878590	1227700	756570	1279500	1E+06	2E+06	2E+06	1E+06	1417100	1115400	1378900	797870	1117900	744370	1277000	960230	1470500	1060400	870070	1291100	836270
MEDP159	Adenine	Nucleotide met	10181213	1E+07	3E+07	9E+06	7607500	12189000	9762000	1.7E+07	8455400	7919900	1E+07	7E+06	7E+06	8E+06	1.1E+07	1.3E+07	10757000	14163000	6565800	90469000	17891000	30746000	72703000	16678000	1E+07	5622100	12448000
MEDP160	Adenosine	Nucleotide met	1.15E+08	1E+08	9E+07	1E+08	95437000	148620000	9.1E+07	1.2E+08	1.4E+08	1.3E+08	8E+07	7E+07	1E+08	1E+08	1.3E+08	1.2E+08	71748000	78411000	128590000	1.36E+08	58520000	263310000	56698000	65565000	6E+07	49235000	63983000
MEDP161	Adenosine 5'-	Nucleotide met	126537.4	110745	173083	115850	125970	70089	102330	128440	156120	182590	130910	39249	104690	202270	160500	109610	34970	36081	198590	101460	115160	781190	91840	73465	65834	67705	88013
MEDP162	C-Di-Amp	Nucleotide met	70783.38	61013	70935	113760	34517	88612	70683	9	106590	51556	100540	79272	25400	7262.3	126920	9956.1	96803	142480	9	64941	9	81302	64723	94511	107490	45855	108650
MEDP163	Cytidine	Nucleotide met	10138425	1E+07	6E+06	1E+07	6850500	15049000	9162800	9456600	1.2E+07	5605500	1E+07	1E+07	1E+07	1E+07	1.3E+07	9432000	6971100	13034000	5157700	9866800	4260400	8720800	5094800	4787000	6E+06	3265900	5087000
MEDP165	Cytosine	Nucleotide met	3650013	4E+06	2E+06	4E+06	7468300	2318600	1818100	1819000	4624600	5201100	2E+06	3E+06	8E+06	5E+06	3149500	3946200	2896400	2513800	1680900	4068400	2362000	944780	2319400	2722700	2E+06	1376900	3483000
MEDP166	Deoxyguanosi	Nucleotide met	8762425	1E+07	8E+06	1E+07	21788000	2695400	3720000	2780500	9669100	1.5E+07	2E+06	4E+06	3E+07	2E+07	4715500	1.1E+07	9270400	3576500	4582600	13513000	11299000	1985800	6863100	10377000	5E+06	3817200	7957000
MEDP167	Guanosine	Nucleotide met	27291000	3E+07	9E+06	3E+07	32767000	39095000	2.7E+07	1.2E+07	2.9E+07	2.4E+07	2E+07	3E+07	5E+07	3E+07	3.3E+07	3E+07	23570000	37340000	18302000	14263000	3631800	14021000	3524100	7782700	9E+06	6079900	11102000
MEDP170	Hypoxanthine	Nucleotide met	87079750	8E+07	1E+08	1E+08	73863000	106340000	1E+08	8.9E+07	6E+07	7.2E+07	9E+07	6E+07	5E+07	6E+07	8.8E+07	8.9E+07	84700000	1.01E+08	78242000	1.52E+08	137600000	40329000	1.27E+08	157070000	9E+07	77789000	1.18E+08
MEDP174	Purine	Nucleotide met	170036.3	204130	144868	188360	168020	189020	197010	179380	106810	155100	176590	178920	138740	185250	156080	186760	281220	368000	138070	162130	70173	147920	79522	189520	184120	115480	210080
MEDP176	Thymidine	Nucleotide met	1300585	1E+06	1E+06	2E+06	2977800	519790	587420	1040500	1158500	1975700	520570	938550	3E+06	2E+06	711980	1501000	1608200	856830	1095400	1336000	1493900	764810	632680	1656600	852050	879310	1218500
MEDP177	Thymine	Nucleotide met	15439500	1E+07	2E+07	2E+07	26293000	13682000	1.1E+07	1.3E+07	1.1E+07	2E+07	1E+07	7E+06	1E+07	2E+07	1E+07	1.9E+07	13907000	7383500	9519200	25610000	20585000	4799500	21708000	29872000	1E+07	11910000	17414000
MEDP180	B-Nicotinamic	Nucleotide met	1021294	558564	3E+06	561260	465350	1038500	1394400	1253300	801340	1173100	1E+06	318340	360160	893900	474750	562030	304890	273540	1280900	565710	2031900	14912000	695850	688640	667220	550280	715080
MEDP184	3,3',5-Triiodo-	Hormones	85590.38	93027	95668	103550	95271	100200	67523	86781	73784	75248	82366	89593	98851	84526	91046	88092	133630	90497	67978	67059	68058	111750	137700	84703	92802	94633	108640
MEDP185	Epinephrine	Hormones	6625900	7E+06	7E+06	6E+06	7186100	5504200	6276400	6725300	6524300	8595800	6E+06	7E+06	1E+07	8E+06	6137500	4855400	9044700	4841900	8824800	5426000	10080000	7225900	7532200	7410600	8E+06	8596800	5952500
MEDP186	L-Thyroxine	Hormones	272736.3	309445	304045	353790	317320	360910	235510	218870	225050	244180	226260	362730	310330	260520	300140	349510	333570	330410	228350	275480	181420	310010	396280	329910	314030	297850	327380
MEDP188	Norepinephrir	Hormones	2526725	2E+06	2E+06	3E+06	3108800	2821900	2371000	2195900	2220400	2396200	2E+06	2E+06	2E+06	2E+06	2086700	2530700	1860400	2365600	2300900	3381700	1904600	2131800	2466400	2473600	2E+06	2090100	2009300
MEDP189	Progesterone	Hormones	143256.3	163319	160811	120980	110820	155940	149040	178740	155220	127440	147870	158700	181070	172890	187050	174930	146880	137260	147770	139810	149720	150860	145140	170530	172240	154900	203290
MEDP205	L-Carnitine	Camitine	12389175	1E+07	2E+07	1E+07	18364000	11763000	8669600	1.1E+07	8746000	1.9E+07	8E+06	7E+06	2E+07	2E+07	8426200	1.3E+07	14320000	6557600	18099000	13105000	22472000	15751000	24949000	12735000	8E+06	14488000	8924700
MEDP211	Melatonin	Tryptamines Ar	623268.8	740084	737201	957930	532000	789120	498340	656320	481120	605360	465960	635650	949410	797200	573330	808050	1265200	492390	399440	894210	538540	621470	669110	1238500	639000	693780	603000
MEDP212	N-Acetyl-5-H	Tryptamines Ar	263748.8	375330	192398	295250	316950	353520	175420	187880	410370	241300	129300	425380	823910	373610	192050	520390	306640	179750	180910	260720	82084	288590	139930	230190	185750	162410	189510
MEDP216	D-Arabitol	Carbohydrate r	221927	162873	115646	131670	213610	121660	428950	195720	136050	453050	94706	202020	276630	196800	67614	166260	184790	70869	138000	108480	158740	94697	188700	124240	120660	47624	82025
MEDP218	D-Mannitol	Carbohydrate r	103186	86109	103939	63679	85001	103060	90809	137130	116930	77639	151240	82532	87029	132780	70322	132270	60698	45839	77403	53494	123190	117780	182180	75574	89283	108750	81261
MEDP223	A-D-Glucose	Carbohydrate r	2.13E+08	2E+08	2E+08	2E+08	2.29E+08	217820000	1.7E+08	2E+08	2.2E+08	2.5E+08	2E+08	2E+08	2E+08	2E+08	1.7E+08	2.1E+08	220860000	1.77E+08	229930000	2.18E+08	214950000	192450000	2.21E+08	195090000	2E+08	188400000	1.51E+08
MEDP224	D-Fructose	Carbohydrate r	2.12E+08	2E+08	2E+08	2E+08	2.34E+08	219030000	1.7E+08	2.1E+08	2.2E+08	2.4E+08	2E+08	2E+08	2E+08	2E+08	1.7E+08	2.1E+08	219890000	1.82E+08	232640000	2.17E+08	213060000	199070000	2.18E+08	194240000	1E+08	187990000	1.59E+08
MEDP225	D-Galactose	Carbohydrate r	2.11E+08	2E+08	2E+08	2E+08	2.34E+08	217840000	1.6E+08	2E+08	2.2E+08	2.5E+08	2E+08	2E+08	2E+08	2E+08	1.7E+08	2.1E+08	222230000	1.79E+08	226020000	2.19E+08	210140000	187290000	2.21E+08	191200000	2E+08	193490000	1.56E+08
MEDP226	D-Glucose	Carbohydrate r	2.07E+08	2E+08	2E+08	2E+08	2.31E+08	215440000	1.6E+08	1.9E+08	2.1E+08	2.4E+08	2E+08	2E+08	2E+08	2E+08	1.7E+08	2.1E+08	213110000	1.82E+08	222640000	2.17E+08	204300000	187710000	2.17E+08	189750000	1E+08	187410000	1.53E+08
MEDP227	D-Mannose	Carbohydrate r	2.05E+08	2E+08	2E+08	2E+08	2.23E+08	213770000	1.6E+08	2E+08	2.1E+08	2.3E+08	2E+08	2E+08	2E+08	2E+08	1.7E+08	2.1E+08	218740000	1.75E+08	222520000	2.14E+08	200070000	186920000	2.16E+08	183660000	1E+08	176960000	1.5E+08
MEDP229	Lactose	Carbohydrate r	138800.5	133840	154176	147460	199320	104850	80274	132400	137060	182840	126200	106850	151240	137720	109230	136580	133120	75451	220530	187180	171880	184520	185200	154640	111170	127330	111490
MEDP230	Maltose	Carbohydrate r	60585.38	44758	46364	60951	69807	84198	67676	29420	56958	50708	64965	44756	60918	29017	35615	43981	43019	60564	40190	57405	31918	52058	26903	65290	56049	28572	52719
MEDP234	D-Glucono-1,	Carbohydrate r	277975	306310	221249	442690	267210	322640	170340	242180	290860	367990	119890	376610	228870	315320	138700	495210	402860	172970	319940	241360	206410	241880	297660	236090	175280	164940	206370
MEDP240	Pyridoxine	Co-Enzyme Fa	6095313	8E+06	5E+06	1E+07	8407500	4422100	5635300	4447300	5445100	5563300	5E+06	4E+06	1E+07	9E+06	4287800	8686700	4232600	5546700	12986000	10051000	3301400	6236600	2616800	6394400	4E+06	3844100	5927400

MEDP241	Orotic Acid	Co-Enzyme Fa	598480	619103	612654	559810	934900	465260	467710	531340	582220	728660	517940	775030	854140	671720	514920	560210	521630	509390	545780	570370	866200	431210	429240	643710	665790	650310	644400
MEDP242	Nicotinamide	Co-Enzyme Fa	385143.8	381764	1E+06	173810	218900	513710	426110	801940	210830	311950	423900	190200	651910	303450	275630	342180	332930	293160	664650	145440	220970	9484000	109070	139240	271560	286520	305580
MEDP244	All-Trans-13,1	Co-Enzyme Fa	390422.5	404255	510893	217180	248570	496870	344280	278520	299180	232680	1E+06	530070	400930	316920	774320	322760	337960	183520	367560	210670	134740	759640	300690	883550	542050	659740	596060
MEDP247	Nicotinuric Ac	Co-Enzyme Fa	819138.3	928268	9	9	9	4224200	9	1492200	9	355930	480740	9	9	1E+06	9	2115800	9	4059100	9	9	9	9	9	9	9	9	9
MEDP249	Pantothenol	Co-Enzyme Fa	31529.63	26571	31680	35693	33930	33380	23365	25029	30245	51074	19521	20968	32810	28051	23512	28275	26054	17456	35440	40284	23865	32679	29891	42555	24762	27238	32167
MEDP250	Riboflavin	Co-Enzyme Fa	2.29E+08	3E+08	2E+08	3E+08	3.13E+08	138190000	2.6E+08	2.6E+08	2.2E+08	2.4E+08	1E+08	3E+08	3E+08	3E+08	2.5E+08	2.3E+08	212020000	2.44E+08	221730000	2.42E+08	270020000	237770000	2.16E+08	257710000	2E+08	182540000	2.28E+08
MEDP251	Trigonelline	Co-Enzyme Fa	23469913	3E+07	5E+06	#####	48589000	11520000	8380300	1.7E+07	4.3E+07	3E+07	1E+07	3E+07	7E+07	4E+07	2.1E+07	2.7E+07	11935000	17481000	6808900	4933300	2243400	5742700	2882600	5059000	6E+06	5857800	6161300
MEDP252	Isonicotinamic	Co-Enzyme Fa	405058.8	358548	1E+06	227220	336600	465020	496710	757380	236910	226660	493970	175300	537780	297010	290490	176980	327490	373370	689960	154820	174710	9981600	122760	154840	199770	297080	214290
MEDP274	Indole-2-Carb	Indole And Its I	324518.8	327860	274733	347820	352940	297020	227790	351190	351370	417620	250400	288260	333710	345040	249710	471830	356330	194670	383330	328040	253880	337610	270160	304020	248490	217600	238060
MEDP275	Indole-3-Acet	Indole And Its I	2176163	2E+06	2E+06	2E+06	2380300	2204800	2055500	2350300	2030000	2502500	2E+06	2E+06	2E+06	2E+06	1620600	2410500	2554000	2184600	2235600	1392400	1426000	1588600	1685400	1859800	2E+06	1617300	1667800
MEDP276	Indole-3-Carb	Indole And Its I	317828.8	314760	223295	230930	128170	725750	473850	345460	177450	138140	322880	234870	207620	187320	128760	434110	378030	860670	86696	215700	80030	158820	123180	346790	414900	126490	320450
MEDP277	Methyl Indole	Indole And Its I	394537.5	364664	339484	353200	238740	369420	538750	594030	410410	276170	375580	316440	316670	646250	291340	257160	374370	566360	148720	476480	230920	208530	214270	265280	368190	188580	763620
MEDP278	N-(3-Indolyla	Indole And Its I	639045	2E+06	126150	998830	1331000	889980	380420	265160	567980	439370	239620	3E+06	5E+06	1E+06	783830	1724600	278890	465940	258020	223940	73644	76622	147270	160370	121490	106340	99521
MEDP280	1,3,7-Trimethy	Organic Acid A	64276.63	53172	53348	83039	72347	38241	63435	64465	39549	86095	67042	61801	59868	66007	36533	72932	37889	39073	51273	48443	39308	55506	54875	50165	53640	56388	68462
MEDP281	1,3-Diaminop	Organic Acid A	92830.25	86761	71203	106740	116170	77509	93971	69538	80464	145120	53130	77519	80417	106790	57331	108680	78084	81687	103580	120090	42036	90062	30650	77901	84910	35968	88004
MEDP285	2,6-Diaminoo	Organic Acid A	763072.5	698053	758030	677140	1090700	610120	716050	931100	489780	980810	608880	710880	797150	847590	511490	666450	511130	477730	1062000	627310	1137700	723170	1067300	550360	741900	742510	473990
MEDP289	3,4,5-Trimeth	Organic Acid A	414288.8	593250	343379	573880	621290	429140	449070	199570	550210	219840	271310	745420	1E+06	630510	433280	527040	521200	452420	230030	513140	125940	439950	114930	500690	360200	204400	487780
MEDP295	4-Acetamidob	Organic Acid A	811677.5	888014	654603	781480	938430	1106800	621810	879860	767960	730940	666140	594270	756380	890730	630510	1355100	713320	1090200	1073600	821070	563460	690070	546230	891740	577490	512420	634340
MEDP296	4-Guanidinob	Organic Acid A	69839625	7E+07	4E+07	9E+07	90933000	77368000	4.1E+07	6.6E+07	5.8E+07	8.1E+07	6E+07	9E+07	8E+07	4E+07	8.2E+07	7.2E+07	47994000	54296000	122520000	37257000	41116000	29898000	43791000	36085000	4E+07	54384000	39840000
MEDP297	5-Aminovaleri	Organic Acid A	2.24E+08	2E+08	2E+08	2E+08	2.22E+08	238440000	1.9E+08	2.2E+08	2.6E+08	2.4E+08	2E+08	2E+08	2E+08	2E+08	2.2E+08	2.2E+08	263390000	1.85E+08	210280000	2.48E+08	223790000	227730000	2.12E+08	213420000	2E+08	267840000	2.09E+08
MEDP298	6-Aminocapro	Organic Acid A	9039988	1E+07	9E+06	1E+07	9581100	9820400	9367500	8149500	5847400	1E+07	9E+06	9E+06	9E+06	1E+07	7365900	1.1E+07	15371000	20724000	8962000	8464300	5336700	8094000	6427000	12644000	1E+07	6072100	11331000
MEDP299	7-Methyluric /	Organic Acid A	269471.3	268743	260986	258800	284700	251730	288460	232850	277640	312160	249430	237870	281010	284700	255400	270060	229900	282990	308010	217850	296460	285690	264870	236210	243200	230380	313230
MEDP300	Adipic Acid	Organic Acid A	1022334	1E+06	1E+06	880510	1140300	883250	1104500	1032200	930290	993820	1E+06	830640	1E+06	928850	790720	938600	1121800	1344600	1185300	1188300	1056900	1260400	1206700	853280	878820	1198800	1192900
MEDP303	Chlorogenic A	Organic Acid A	13124288	2E+07	1E+07	1E+07	16030000	17900000	1.2E+07	2E+07	6667400	9042900	1E+07	2E+07	2E+07	1E+07	1.5E+07	2E+07	38164000	24708000	6751800	7387600	8769300	7599000	18562000	21110000	1E+07	8981300	5187900
MEDP305	Creatinine	Organic Acid A	38103875	5E+07	4E+07	4E+07	62145000	25534000	3E+07	4.9E+07	4.9E+07	1.8E+07	3E+07	7E+07	9E+07	7E+07	4.5E+07	4.5E+07	52021000	31619000	38521000	28336000	48473000	35353000	47027000	30939000	3E+07	42626000	41883000
MEDP306	D-2-Aminobu	Organic Acid A	2505850	3E+06	3E+06	2E+06	2152200	2934300	2387600	3504800	2010000	2429600	2E+06	3E+06	3E+06	5E+06	2683400	3960600	2704600	1921400	2439800	1891400	2292900	3480000	3671400	3034100	2E+06	3371700	2485700
MEDP309	D-Pantotheni	Organic Acid A	275018.8	498305	188438	250290	319180	232330	144640	300930	382090	430720	139970	500450	1E+06	492290	170830	450790	439190	383090	147100	126350	126410	185570	176540	198780	305300	132850	255700
MEDP310	D-Pipecolinic	Organic Acid A	10260500	1E+07	1E+07	7E+06	13159000	7884200	8810900	1.2E+07	9425900	1.4E+07	9E+06	9E+06	1E+07	1E+07	9569400	1.1E+07	9021700	9288700	13399000	8674400	15144000	13863000	13552000	9250900	9E+06	11069000	9504800
MEDP313	Guanidineacet	Organic Acid A	2.27E+08	2E+08	2E+08	2E+08	2.19E+08	233660000	2.1E+08	2.3E+08	2.7E+08	2.4E+08	2E+08	2E+08	2E+08	2E+08	2.2E+08	2.2E+08	238240000	1.84E+08	213770000	2.46E+08	223740000	246390000	2.16E+08	211120000	2E+08	269030000	2.11E+08
MEDP317	Hydrocinnami	Organic Acid A	30149.63	47633	66456	25205	58800	38080	66814	9	24922	9	27358	70157	49754	60819	46643	56247	9	43573	53865	83309	80017	33110	50665	93482	65563	55275	70224
MEDP318	Kinurenine	Organic Acid A	49806.75	61767	65237	46466	84343	40217	35382	67053	36437	88547	9	33615	80571	111510	36763	55329	52316	17499	106530	98046	67470	102960	67072	64727	31859	46985	42774
MEDP319	Kynurenic Aci	Organic Acid A	18402875	3E+07	1E+07	2E+07	32850000	18253000	1.3E+07	1.4E+07	2.2E+07	2.1E+07	1E+07	4E+07	6E+07	3E+07	1.7E+07	2.3E+07	13232000	17546000	17628000	16406000	9481600	13655000	10697000	13230000	1E+07	11210000	11818000
MEDP320	L-2-Aminobu	Organic Acid A	3729425	4E+06	4E+06	3E+06	3358000	4163900	3550000	4987500	3032000	3715100	4E+06	4E+06	4E+06	7E+06	4046200	5036400	3936400	3656800	3750900	3083500	3560500	6013500	4611100	4566000	4E+06	4465800	3211000
MEDP322	L-Homoserine	Organic Acid A	3087263	3E+06	4E+06	3E+06	2744300	2596600	3325200	4141800	1766100	3315700	4E+06	3E+06	3E+06	3E+06	3206900	2568200	4230500	3453800	4242000	3079000	7504300	3833700	5939600	3578200	4E+06	4558600	3219800
MEDP325	Maleic Acid	Organic Acid A	6614775	7E+06	8E+06	6E+06	6926700	7331400	6387200	6447700	5173900	9181200	5E+06	6E+06	9E+06	9E+06	5367600	7878600	7862400	5008000	7784000	9452500	9418500	7443400	9045500	7034900	6E+06	7179900	6245700
MEDP326	N'-Formylkyn	Organic Acid A	351506.3	303153	279208	442330	459660	250130	246040	360240	363160	436690	253800	296580	314980	428240	258510	387360	288690	191060	259800	349270	265700	291510	423570	254430	189980	255110	204090
MEDP327	N-Γ-Acetyl-N	Organic Acid A	168738.3	174371	95752	211160	251550	147560	107580	137100	178740	220870	95346	244330	389370	237900	200030	9	167060	9	156260	121020	79169	84375	113350	148920	101060	118110	9
MEDP328	P-Aminohippu	Organic Acid A	78183.38	153318	3055.5	61269	179940	131070	32569	52599	61650	81060	25310	242700	498720	153480	52800	171010	24552	54801	28481	24381	9	9	9	9	9	9	9
MEDP334	Vanillic Acid	Organic Acid A	175517.5	173949	157149	177280	160620	180070	240300	136890	144670	194650	169660	145100	133150	180270	153600	241810	146720	245030	145910	219230	119280	61263	109640	259650	182670	108830	196630
MEDP336	Lysopc 14:0	Lipids-Phospho	5185563	7E+06	8E+06	4E+06	2176200	6959400	4980400	6717900	4631400	2203100	9E+06	7E+06	4E+06	5E+06	5540500	4890100	8097600	16442000	2224700	4262100	4126900	17981000	3526900	13870000	9E+06	5377800	8096100
MEDP338	Lysopc 16:0	Lipids-Phospho	1.24E+08	1E+08	1E+08	1E+08	53728000	154520000	1.3E+08	1.4E+08	1.3E+08	5.3E+07	2E+08	2E+08	1E+08	9E+07	1.4E+08	1.1E+08	167490000	2.66E+08	43749000	75735000	70012000	182510000	64482000	204320000	2E+08	127790000	2.01E+08
MEDP340	Lysopc 16:1	Lipids-Phospho	11742800	2E+07	2E+07	1E+07	4577500	14145000	1.4E+07	1.5E+07	1.2E+07	4409500	2E+07	2E+07	1E+07	1E+07	1.3E+07	1E+07	21037000	37251000	5335300	9138700	6997900	24836000	5176200	25981000	2E+07	11137000	23182000
MEDP342	Lysopc 18:0	Lipids-Phospho	45931875	5E+07	6E+07	3E+07	17594000	55352000	4.4E+07	5.1E+07	3.9E+07	1.9E+07	1E+08	7E+07	3E+07	3E+07	5.9E+07	4.4E+07	48069000	1.04E+08	26403000	20963000	19776000	83170000	26920000	100790000	9E+07	59259000	62049000
MEDP344	Lysopc 18:1	Lipids-Phospho	74451750	9E+07	1E+08	5E+07	26922000	82612000	8E+07	8.9E+07	7.4E+07	2.7E+07	2E+08	1E+08	5E+07	6E+07	9.2E+07	6.8E+07	131180000	2.12E+08	20453000	40520000	46860000	127870000	26799000	155710000	2E+08	76057000	1.43E+08
MEDP346	Lysopc 18:2	Lipids-Phospho	46124125	5E+07	4E+07	6E+07	52552000	55719000	3.5E+07	3.5E+07	3.4E+07	4.3E+07	6E+07	6E+07	5E+07	6E+07	8.2E+07	4.8E+07	20414000	32201000	73175000	50712000	7321000	56849000	18934000	47896000	6E+07	31841000	56095000
MEDP350	Lysopc 20:1	Lipids-Phospho	1127253	1E+06	2E+06	851560	438680	1217100	1061800	1474300	919010	485370	3E+06	2E+06	717490	814070	1261000	902660	1654900	3286700	465690	492090	846100	2331500	789590	2384000	2E+06	1080600	1924200
MEDP352	Lysopc 20:2	Lipids-Phospho	1097241	1E+06	1E+06	730900	381810	1195700	936490	994160	947880	333890	3E+06	2E+06	616220	684770	1262400	980130	1043300	4679100	469160	531370	461930	2346100	433750	2577700	2E+06	1052500	1926600
MEDP357	Urocanic Acid	Organic Acid A	11137575	1E+07	1E+07	1E+07	12671000	15262000	1.2E+07	1E+07	8273600	1.3E+07	7E+06	1E+07	2E+07	2E+07	5153400	1.1E+07	11611000	8982200	6448000	16324000	6892400	5962400	12343000	14270000	1E+07	11455000	11225000
MEDP358	Pipecolinic Ac	Organic Acid A	1.48E+08	1E+08	2E+08	1E+08	1.63E+08	129430000	1.4E+08	1.7E+08	1.3E+08	1.7E+08	2E+08	1E+08	1E+08	1E+08	1.5E+08	1.2E+08	118370000	96790000	181040000	1.31E+08	230030000	166810000	1.87E+08	138340000	1E+08	169440000	1.34E+08
MEDP362	Neopterin	Pteridines and	141425.8	118704	121289	156370	156440	116550	97846	192340	106330	183200	122330	128040	139670	167790	94276	94105	87107	92601	146040	103480	151860	88380	116960	78954	123900	165510	141270
MEDP363	Biopterin	Pteridines and	280668.6	375350	91676	198600	188600	666100	9	436770	274920	266170	214180	291420	1E+06	9	201740	657750	374000	454270	9	210680	9	85088	180090	9	180530	76994	9
MEDP368	Taurocholic A	Organic Acid A	321291.3	337796	359700	312950	381040	267260	307260	349810	346470	307950	297590	391560	338550	330200	284600	337570	402490	237160	380240	419560	336190	303400	410750	311980	378650	344680	372390
MEDP370	2-(Formylamir	Benzene and su	13083400	2E+07	1E+07	1E+07	12381000	15449000	1.5E+07	1.3E+07	8869200	1.2E+07	2E+07	1E+07	1E+07	1E+07	1.2E+07	1.2E+07	19699000	29195000	9973700	9952000	5969800	8088700	7000500	15162000	1E+07	8841600	17491000
MEDP373	N-Acetylglycir	Amino Acid m	49105375	5E+07	5E+07	5E+07	52202000	45731000	3.2E+07	6.8E+07	4.7E+07	5.9E+07	3E+07	4E+07	5E+07	8E+07	4.7E+07	4.7E+07	37433000	20299000	62567000	53349000	39598000	49028000	49529000	57572000	6E+07	43284000	57174000
MEDP379	3-Hydroxyhip	Organic Acid A	11961400	2E+07	4E+06	1E+07	23001000	7370300	6342900	7981700	1.7E+07	1.5E+07	6E+06	3E+07	5E+07	2E+07	1.4E+07	9586400	4112400	1582400	6412400	4075500	2624000	3023000	5040900	5030400	5E+06	4677900	3901000
MEDP380	2-(Dimethylar	Nucleotide met	507798.8	552493	287804	423820	420910	1045400	444660	511780	350730	371210	493880	312660	335400	497830	310960	1215700	420970	950030	376390	240600	250360	383500	222810	324210	288410	236210	356330
MEDP381	7-Methylguan	Nucleotide met	32434250	4E+07	3E+07	3E+07	32165000	41382000	3.4E+07	3.1E+07	2.2E+07	3E+07	4E+07	3E+07	3E+07	4E+07	3E+07	3.4E+07	48985000	68291000	26380000	26863000	14788000	21808000	17528000	40539000	4E+07	23079000	43425000
MEDP382	8-Hydroxy-2-	Nucleotide met	84958.63	88508	53401	121530	201510	19457	62645	33348	113110	128060	9	102550	265780	132570	9	9	83833	76764	46546	62313	139930	9	71702	55944	37264	9	60039
MEDP384	Isoxanthopteri	Pteridines and	278490	378912	187919	370230	228490	252250	301990	318510	186050	229560	340840	512620	213380	95108	186800	875780	320730	437560	389320	247470	139550	167630	107780	236340	200460	118490	285630
MEDP385	L-Sepiapterin	Pteridines and	8659.5	635190	33126	9	9	9	9	12856	30566	25809	9	36243	30478	9	35638	2708700	2227500	9	42943	51361	26520	136290	24572	9	9	13108	13141
MEDP386	N-Acetylthrec	Amino Acid m	1370891	2E+06	2E+06	1E+06	1382900	1391300	1664200	1273600	1184400	995230	2E+06	1E+06	2E+06	2E+06	1529300	2002900	4267600	2461700	3181900	3920900	1490100	1385600	3090400	2044100	2E+06	1587600	1380100
MEDP387	H-Homoarg-(	Amino Acid m	63031.88	162402	202586	66056	235290	28985	30026	34780	9	109100	9	118160	253780	193810	97214	191510	141280	116870	186590	285570	346270	198100	333980	150730	79465	180660	45909
MEDP389	Pantetheine	Co-Enzyme Fa	7067000	7E+06	9E+06	7E+06	5676500	6265700	6566200	8080700	8712400	6512100	8E+06	9E+06	9E+06	7E+06	7377600	6223100	6791900	7503900	7501200	9439300	6673200	14756000	7958100	6349700	8E+06	7513900	8168000
MEDP393	Pyridoxine 5'-	Co-Enzyme Fa	631031.3	635111	658823	738740	771190	518290	463950	410900	633850	985690	525640	518780	643560	686590	637670	751280	536720	651430	654860	981520	1015100	666190	805870	500120	387610	587090	327080
MEDP401	5'-Deoxy-5'-(	Nucleotide met	9646588	8E+06	1E+07	7E+06	7518900	15386000	1.2E+07	8301700	1E+07	8733400	7E+06	4E+06	8E+06	9E+06	7051800	1.7E+07	8555500	6300400	5078900	19564000	8161900	18628000	8000100	9737300	7E+06	7412200	14752000
MEDP402	Guanine	Nucleotide met	13406950	2E+07	4E+06	2E+07	17273000	18622000	1.4E+07	5544100	1.4E+07	1.2E+07	1E+07	2E+07	2E+07	2E+07	1.8E+07	1.6E+07	10946000	20008000	9479100	6910600	1825400	6195400	1668700	3272900	4E+06	2926800	4035500
MEDP403	Deoxycytidine	Nucleotide met	1240503	1E+06	1E+06	2E+06	2422700	776650	727990	764900	1373200	1435800	612480	661640	3E+06	2E+06	1057100	1875300	1093700	686610	954780	2055800	1431900	654750	836110	1556700	702450	852380	1223800
MEDP404	Sarcosine	Amino Acid m	2849375	3E+06	5E+06	4E+06	2564200	2842000	2638200	2666600	3017800	2664200	3E+06	3E+06	3E+06	3E+06	3408100	3363100	3315700	3365700	3681500	3496800	3453200	6445200	4207200	4280600	5E+06	4821800	5472700
MEDP405	3-Aminoisobu	Amino Acid m	1365244	1E+06	1E+06	1E+06	1060500	1614300	1429100	1228100	1723300	895050	2E+06	2E+06	2E+06	1E+06	1386500	1772800	1513500	1010600	1255600	2267000	1209100	1134600	1159600	1748500	1E+06	1494100	1424300
MEDP407	Vitamin A	Co-Enzyme Fa	94299.88	66380	115389	67049	114990	96774	80219	86800	84447	112670	111450	42175	142380	79892	81241	9	83046	9	102290	133160	134150	102720	125320	114570	89005	113440	110750
MEDP410	Trans-Citridic	Organic Acid A	2054513	2E+06	2E+06	2E+06	2068800	2465500	1547300	1842200	2119600	2342900	2E+06	2E+06	2E+06	2E+06	1661200	2184600	2156600	1707000	2351600	2121500	2328800	2691800	2471400	2017600	2E+06	2833600	1920400
MEDP416	6-Phosphoglu	Carbohydrate r	15052125	3E+07	2E+07	1E+07	18016000	11035000	2.3E+07	1.4E+07	1.3E+07	1.9E+07	1E+07	6E+07	2E+07	2E+07	4.2E+07	1.8E+07	6583500	31100000	29374000	17403000	21862000	15781000	13451000	12560000	2E+07	19942000	32136000
MEDP417	D(-)-Threose	Carbohydrate r	951213.8	974039	887696	881610	1123500	1083000	840760	907190	785050	1261200	727400	957140	2E+06	1E+06	559560	906920	913840	891390	506160	1276200	617300	611330	1010300	996670	859320	827250	903200
MEDP423	Phosphoric Ac	Organic Acid A	9843975	7E+06	8E+06	1E+07	9259600	10851000	5336200	5485100	8422400	9801500	2E+07	4E+06	4E+06	5E+06	1.9E+07	4657200	1767300	2404000	12267000	12322000	8661100	7174700	11050000	6777500	3E+06	8224100	4214000
MEDP426	Xanthurenic A	Organic Acid A	1454300	2E+06	2E+06	1E+06	1230000	1345800	1591800	1682400	1586800	1327100	2E+06	1E+06	1E+06	2E+06	1898800	1219300	1917400	2088700	1191700	909830	1461700	1369300	1606400	1448800	2E+06	2062900	1837100

MEDP428	Cholesterol	Lipids	1342461	3E+06	2E+06	985620	1233500	1086200	2657700	697350	1211500	1874400	993420	4E+06	1E+06	3E+06	5669000	1493900	830960	5204800	3566500	1299100	1166000	1286600	1207000	1062500	1E+06	1781200	3546800
MEDP429	Punicic Acid	Lipids_Fatty Aci	7422025	8E+06	7E+06	4E+06	3283800	7551400	1E+07	1.1E+07	5615800	4020700	1E+07	9E+06	4E+06	7E+06	5633200	6988400	13857000	14051000	3793100	7427300	5777000	8900900	2347800	6027900	8E+06	5000200	8635000
MEDP430	2-Aminoadipi	Organic Acid A	6968088	7E+06	7E+06	5E+06	7897700	7771800	6523100	7014900	5405600	1E+07	6E+06	5E+06	8E+06	7E+06	5986000	6748600	5466500	4678300	10093000	5213200	9457600	6883500	9567100	6197800	5E+06	6068200	6177500
MEDP431	Hordenine	Benzene and su	161006.3	158208	140889	128280	184490	190420	160430	181890	125470	161520	155550	134100	200870	189860	118640	136690	168810	173950	142740	117570	105450	123950	134600	168380	176020	148790	152350
MEDP433	4-Aminoindol	Indole And Its I	1576938	2E+06	1E+06	2E+06	1860200	1604700	1367700	1403000	1505500	2022700	1E+06	2E+06	2E+06	2E+06	1297000	2205700	1991500	1127600	2586200	1474200	1362800	1255200	1269700	1653900	1E+06	1376100	1166800
MEDP434	Lysopc 17:0	Lipids-Phosphc	4477800	5E+06	6E+06	3E+06	1624000	7193500	4198900	4545600	3738400	1394200	1E+07	7E+06	3E+06	3E+06	5892600	4201100	4022300	15177000	2059300	2578900	2460400	8340100	2854800	11414000	8E+06	5360800	9275600
MEDP435	6-Methylmerc	Nucleotide met	8246238	1E+07	7E+06	8E+06	8492500	9991300	9045400	8147200	5429200	7241100	9E+06	8E+06	7E+06	9E+06	7451700	8606100	12789000	17069000	6402900	6136200	3737800	5454700	4419300	9795400	8E+06	5943600	10483000
MEDP436	N-Feruloyl Pu	Phenolamides	13050438	7E+06	1E+07	1E+07	14385000	9047500	1.4E+07	1.9E+07	1E+07	1.5E+07	1E+07	1E+07	1E+07	2E+07	6932500	7892500	1869200	5367700	201290	10690000	10975000	10511000	12075000	15226000	1E+07	9982400	12170000
MEDP437	Ergothioneine	Organic Acid A	276047.5	240160	227703	325310	457090	250220	291970	202650	222180	198550	260410	343000	253470	295890	217210	201410	192040	103890	314370	345010	251130	197090	286700	150590	149350	176990	264760
MEDP440	Methoxyindol	Indole And Its I	83641.75	84850	74882	108090	137430	88153	55602	53312	65187	128930	32430	56223	109740	95132	40588	131950	76060	50880	118230	129020	52962	107950	71055	79488	55647	43241	59693
MEDP441	N-Caffeoyl Pu	Phenolamides	4876500	3E+06	5E+06	5E+06	8560100	2818700	2085100	3881900	5041400	9662600	2E+06	1E+06	8E+06	5E+06	2601100	3538400	331420	91494	9	5412400	4587900	5563000	7298700	4920900	2E+06	5008000	2496900
MEDP442	Sn-Glycero-3	Cholines	13898013	8E+06	1E+07	1E+07	10992000	14159000	8507400	2.2E+07	9914700	1.5E+07	2E+07	8E+06	6E+06	8E+06	6604600	1E+07	6528300	7710700	10727000	5406100	12317000	19141000	8660200	12208000	9E+06	11417000	7644700
MEDP443	10-Formyl-Th	Pteridines and	64968125	4E+07	4E+07	9E+07	94953000	76129000	4.9E+07	2.9E+07	5.8E+07	9.4E+07	3E+07	3E+07	6E+07	4E+07	2.7E+07	7.5E+07	19250000	10718000	79679000	1.3E+08	31938000	37022000	32909000	44403000	2E+07	21476000	23699000
MEDP446	3-Hydroxykyn	Amino Acid m	108387.6	111030	117071	118890	147770	61263	54202	150620	97966	108740	127650	91848	166560	91597	110670	120860	120040	59837	126830	85527	117080	107980	168830	98052	139240	127720	92135
MEDP452	Syringaldehyd	Benzene and su	109577.3	118749	139708	138130	131060	61170	80599	60827	105970	208840	90022	93315	142280	145140	174740	140470	55621	84488	113940	117510	177940	78597	118450	158890	141830	152020	172430
MEDP453	Indole	Indole And Its I	3914763	4E+06	4E+06	4E+06	3699100	3829800	4061000	4119500	3889000	3713600	4E+06	4E+06	4E+06	4E+06	4187100	3552500	3477500	3871100	3815400	3292400	3945000	3367100	3875700	3780700	4E+06	4047200	3932400
MEDP454	Isoquinoline	Benzene and su	161352.4	136881	140933	127220	184740	162320	150780	163390	197770	215080	89519	60327	168130	150690	83445	236870	147180	81805	166600	203270	115850	105340	124770	230860	137000	79384	130990
MEDP456	Piperidine	Benzene and su	2159325	3E+06	2E+06	3E+06	2585600	2466500	2343700	2004200	1297100	2223000	2E+06	2E+06	3E+06	3E+06	1702300	2241100	3670000	4501500	1710000	1939900	1257200	1758900	1631400	2697100	2E+06	1271800	2645200
MEDP457	5-Aminolevuli	Organic Acid A	282342.5	293371	233147	268900	236600	295120	344990	248590	228680	330130	305730	318430	202540	304830	119680	254860	493130	647270	6225.4	249150	156140	177760	187170	359360	368530	75096	291970
MEDP459	Ip7G	Nucleotide met	573667.5	517690	699579	514480	641620	528740	467350	663680	561730	670530	541210	399120	434440	546250	561300	564940	747940	533730	353800	500850	606630	618990	730180	612270	479810	1505800	542100
MEDP494	Lysopc 15:0	Lipids-Phosphc	10717800	1E+07	2E+07	7E+06	3727000	20351000	1.1E+07	9993800	8708400	3386400	2E+07	1E+07	7E+06	7E+06	1.2E+07	9801200	11655000	37008000	3587600	8552100	8508600	20513000	8272600	26458000	2E+07	11665000	30744000
MEDP498	Lysopc 18:3	Lipids-Phosphc	38670250	5E+07	5E+07	3E+07	15034000	39565000	4.7E+07	4.2E+07	4.5E+07	1.6E+07	8E+07	7E+07	3E+07	3E+07	5E+07	3.6E+07	49677000	1.05E+08	14736000	23218000	23614000	51499000	19428000	76474000	7E+07	44890000	66117000
MEDP507	2-Hydroxycin	Benzene and su	7910575	9E+06	8E+06	7E+06	9125100	10445000	7608600	7503800	4956500	8446800	8E+06	7E+06	9E+06	8E+06	7108300	1E+07	10996000	16625000	7142100	6640100	6474000	8496300	5808300	9871700	8E+06	7095500	8611200
MEDP509	trans-3-Indole	Organic Acid A	895573.8	784841	767390	959680	1473800	888500	551940	507460	825110	1540300	417800	498770	984800	886350	416910	1354900	621520	381080	1134400	1400200	517030	1112300	581080	864650	543020	498650	622190
MEDP510	Acetyl-L-carn	Camitine	4119263	5E+06	9E+06	4E+06	3205100	2369500	3298300	5937800	4474300	4277900	5E+06	2E+06	2E+06	1E+07	1856200	4969800	13637000	6728900	1555800	13581000	5013900	6486400	3242300	18219000	8E+06	8205700	7868600
MEDP511	Jasmonic acid	Lipids_Fatty Aci	192209.6	348186	9	285630	569150	78245	111580	76183	180250	236630	9	796370	1E+06	430140	123220	262430	9	9	9	9	9	9	9	9	9	9	9
MEDP513	Hexadecanam	Lipids_Fatty Aci	2846325	4E+06	4E+06	2E+06	2413600	2423700	2985100	4117500	2596300	3306600	3E+06	4E+06	3E+06	4E+06	3846300	3090600	4260800	3124800	6833600	3230500	3590800	3408800	4782600	3967400	4E+06	4776400	4431500
MEDP514	Thiamine	Co-Enzyme Fa	89910625	1E+08	9E+07	9E+07	1.34E+08	73914000	8.5E+07	8.4E+07	8.3E+07	1E+08	7E+07	1E+08	1E+08	1E+08	6.3E+07	9.1E+07	93410000	72623000	116970000	87374000	128820000	83693000	79320000	80646000	7E+07	86977000	92220000
MEDP517	Palmitoylcarni	Camitine	41582625	4E+07	3E+07	5E+07	44407000	47358000	3.5E+07	3.5E+07	3.9E+07	4.4E+07	3E+07	4E+07	5E+07	4E+07	3.3E+07	4.5E+07	34128000	19583000	36395000	41916000	26011000	23370000	35326000	42708000	3E+07	36093000	30839000
MEDP518	DL-Stachydrir	Organic Acid A	23606375	3E+07	3E+07	2E+07	35316000	14117000	2.4E+07	2.4E+07	2.3E+07	3E+07	1E+07	3E+07	4E+07	4E+07	1.4E+07	3.3E+07	32239000	26963000	32803000	28903000	29280000	31086000	31855000	29199000	2E+07	23920000	18189000
MEDP519	L-Norleucine	Amino Acid m	1599513	1E+06	1E+06	2E+06	1918900	1668400	1484300	1346800	1346800	2078700	1E+06	936730	1E+06	1E+06	1000700	1448900	1398600	1286200	1405900	1066200	942900	1283500	1063400	1205300	1E+06	833160	1031000
MEDP523	DL-Carnitine	Camitine	34470375	4E+07	4E+07	4E+07	55751000	27117000	2.3E+07	3.1E+07	2.4E+07	5.5E+07	2E+07	2E+07	4E+07	5E+07	2.4E+07	3.2E+07	42172000	18220000	53254000	31583000	60740000	45273000	70273000	32973000	3E+07	39469000	22986000
MEDP524	DL-Arginine	Amino Acid m	3242725	3E+06	2E+06	3E+06	5706500	3100700	2088800	2098700	2303000	5406100	2E+06	3E+06	9	5E+06	2633500	4375800	3727600	4830000	4992900	2021500	9	2860200	9	2096200	2E+06	3479400	2269600
MEDP525	N-Acetyl-L-al	Organic Acid A	1.4E+08	1E+08	1E+08	#####	1.48E+08	168450000	1.1E+08	1.6E+08	1.2E+08	1E+08	1E+08	2E+08	2E+08	2E+08	1.5E+08	1.4E+08	100210000	1.07E+08	146700000	87228000	70510000	70220000	67465000	128940000	1E+08	140300000	1.06E+08
MEDP528	5-Aminosalicy	Organic Acid A	630527.5	714489	484721	1E+06	992370	370520	536720	645280	423650	594240	370140	316870	1E+06	462050	859640	1117900	497100	240750	1149200	479490	664980	281260	354280	962670	296720	558880	279490
MEDP529	Indole-3-acet	Indole And Its I	5047288	5E+06	4E+06	6E+06	6920500	4617000	3769400	3950600	4767600	7615800	3E+06	4E+06	7E+06	7E+06	4125100	6197200	3956600	2766100	7103900	5683600	3538100	3814400	5240600	4741000	3E+06	4318400	4060800
MEDP530	15-deoxy-δ-1	Lipids_Fatty Aci	441533.8	425456	2E+06	153180	159800	788580	548560	612370	213950	186550	869280	994130	203450	346280	163190	9	400080	1296500	9	191870	455320	933080	287620	1018400	7E+06	1703800	979530
MEDP531	(-)-trans-Cary	Lipids_Fatty Aci	136302.5	133341	142422	164370	164430	128220	132280	142130	95510	109530	153950	188380	113170	140340	81345	166040	107320	126770	143360	161490	105050	125280	97225	127830	202070	132510	187920
MEDP535	Triethyl phosp	Organic Acid A	201248.8	217873	223144	171090	177670	250860	200330	208240	245840	149980	205980	220560	176350	225990	246270	239000	209840	247290	177680	187200	217920	234240	300480	226500	163240	241370	214200
MEDP537	PAF C-16	Lipids	48895750	6E+07	6E+07	4E+07	18917000	58518000	4.9E+07	5.5E+07	4.1E+07	2E+07	1E+08	7E+07	3E+07	3E+07	6.3E+07	4.7E+07	50885000	1.17E+08	28004000	24289000	21641000	90564000	28340000	105480000	1E+08	62218000	68960000
MEDP540	7,8-dihydro-L	Pteridines and	49192.88	65889	31302	95357	78344	77017	51295	9	9	58880	32632	102200	62693	49444	69905	58012	47840	81275	55743	56901	15417	35859	71336	9	34742	36139	9
MEDP541	δ-Valerolactai	Pyridine And Py	4583488	6E+06	4E+06	5E+06	5816400	4535100	3066100	5719800	4484800	4886500	3E+06	3E+06	5E+06	7E+06	4599300	6222600	4876300	9547700	4904400	4057200	3624000	5071600	4323900	4220900	3E+06	3933900	3972500
MEDP545	2-Pyrrolidinor	Benzene and su	1905088	2E+06	2E+06	1E+06	1985600	1028700	1176000	3692200	1758300	2388800	2E+06	2E+06	911330	2E+06	1774300	2737700	2715800	1059200	470890	909030	1146100	583380	1715800	1683600	3E+06	686340	2690100
MEDP546	Oxindole	Indole And Its I	279413.8	307859	246246	270870	278980	331620	218360	228190	292060	354450	260780	232370	271270	298580	237880	358590	369240	210530	484410	268190	260330	245380	225870	301970	206010	240720	221500
MEDP551	N-METHYL (-	Benzene and su	577887.5	565719	496423	663560	687990	522680	429650	527620	616750	717980	456870	523930	604350	626820	443320	737610	635220	349250	605250	558290	453080	549160	470490	534180	492800	467600	445780
MEDP552	alantolactone	Terpenoid	425261.3	442268	176011	535210	400960	427310	318490	407140	463010	422050	427920	386820	426260	626210	573740	526180	518690	206560	273680	282740	9	231440	302380	238110	9	9	353390
MEDP560	Dihydrouracil	Nucleotide met	173522.6	181588	93385	119010	312680	174920	73585	157260	143730	334530	72466	157470	213410	286600	143600	169790	118260	138820	224750	46354	191330	99532	152920	124330	35969	96638	9
MEDP561	Farnesene	Lipids_Fatty Aci	13724.38	51291	159117	9	23849	9	9	66394	9	19507	9	9	9	9	9	9	9	197940	212330	78121	260510	315330	121920	86181	114400	161920	134550
MEDP562	3-(2-Hydroxy	Organic Acid A	581276.3	744059	380034	623680	721510	874190	472850	506200	547310	503080	401390	854380	1E+06	727200	511650	737550	396560	946320	460810	422620	231380	361840	270840	450310	505750	349700	447830
MEDP574	Oleamide	Lipids_Fatty Aci	7337100	9E+06	1E+07	4E+06	5996300	6582400	7162400	1.1E+07	7667800	7477000	9E+06	1E+07	5E+06	8E+06	9386800	9699600	12349000	8176800	10134000	11463000	7041100	9925200	11044000	10442000	1E+07	10762000	13041000
MEDP575	5-amino-1-[3	Nucleotide met	308198.8	272618	285270	275930	393110	355840	394130	224350	267090	258310	296830	247940	237450	259760	255790	304980	325200	200210	349610	434350	230380	260910	245670	275220	239030	292190	304410
MEDP576	N1-Acetylspei	Organic Acid A	78702.38	117134	75675	33678	88169	57942	110810	110790	68352	61979	97899	156230	105200	139800	86172	135580	113570	106570	93950	101950	96369	122600	51543	65829	79663	34488	52954
MEDP577	Isobutyryl carr	Camitine	30858625	4E+07	4E+07	4E+07	33149000	34326000	2.3E+07	3.2E+07	2.8E+07	3.8E+07	2E+07	3E+07	3E+07	5E+07	2.5E+07	4.4E+07	55765000	22910000	23893000	39981000	30855000	26505000	39368000	55108000	3E+07	43117000	24714000
MEDP578	alpha-Cadinei	Lipids_Fatty Aci	139985.6	188496	209438	97882	139560	129030	83353	241110	126620	156690	145640	220420	205450	261840	114270	218040	187760	166340	133850	143220	203750	453240	246260	130080	184110	166550	148290
MEDP583	Asp-Phe metl	Organic Acid A	88195375	1E+08	4E+07	1E+08	95868000	29032000	7E+07	9.6E+07	1.1E+08	1.5E+08	2E+07	8E+07	8E+07	1E+08	8.8E+07	1.7E+08	52152000	81040000	113970000	48242000	14439000	40187000	19995000	67249000	5E+07	48241000	45204000
MEDP584	Pterine	Pteridines and	64536.75	67602	62113	49284	66862	69436	52587	77856	68464	82367	49438	95294	58207	49391	45363	77123	73557	65175	76706	60652	100150	99459	68338	39178	40887	46910	41327
MEDP585	Stearidonic Ac	Lipids_Fatty Aci	135853.8	109588	52445	9	98888	9	83963	317860	111000	99171	375930	140890	62265	256630	9	97689	140750	70060	108410	9	108190	80264	9	9	231060	9	9
MEDP586	N-Acetylphen	Organic Acid A	197797.5	640840	251106	217550	179850	116110	228600	181970	100020	286030	272250	1E+06	216470	398210	135070	357250	415270	2170900	250550	131910	221060	260040	108740	527270	288650	100100	371080
MEDP587	N-Alpha-acet	Organic Acid A	20586500	2E+07	2E+07	2E+07	24610000	20210000	1.9E+07	2E+07	1.8E+07	3E+07	1E+07	2E+07	2E+07	3E+07	1.5E+07	2.8E+07	18339000	17937000	29742000	22412000	22075000	19673000	24310000	21654000	2E+07	18055000	17538000
MEDP590	p-Mentha-1,3	Lipids_Fatty Aci	6028650	7E+06	5E+06	8E+06	8768900	3264800	1.1E+07	2008700	2180100	1969300	1E+07	1E+07	1E+07	3E+06	2642300	3110400	11218000	2155800	2115000	3190100	17613000	4697700	2187500	3898200	3E+06	2960500	2521200
MEDP593	Thioguanine	Nucleotide met	364397.5	407723	253283	405140	549110	300860	251850	305480	444470	430850	227420	454740	655370	433360	310350	462540	389790	254860	300770	375670	220470	174560	304710	284980	199540	244490	221840
MEDP598	2'-Hydroxy-5'	Benzene and su	95597.25	146994	163629	122350	122350	104680	132340	90162	59322	75978	57596	142060	153890	148890	166410	144340	89202	168300	162860	220270	223610	69093	93361	231290	164260	116830	190320
MEDP601	Spermidine	Polyamine	6312163	7E+06	5E+06	8E+06	10443000	5438600	7041300	2137800	5304300	1E+07	2E+06	5E+06	1E+07	8E+06	2771300	8721500	7113400	5288300	10771000	10663000	4139800	5234400	4783200	6449000	3E+06	2322600	3211000
MEDP602	1-(4-Methoxy	Benzene and su	10576425	1E+07	8E+06	1E+07	12764000	11233000	1E+07	8358300	1.2E+07	9796600	9E+06	1E+07	2E+07	1E+07	9076400	1.5E+07	8475000	10596000	10731000	11513000	5090400	7796700	5983000	9063900	9E+06	8325700	9858100
MEDP603	Cuminaldehyc	Lipids_Fatty Aci	1365276	2E+06	1E+06	1E+06	1481300	1666200	1402500	1350400	911910	1267300	1E+06	1E+06	1E+06	1E+06	1290400	1509900	2010500	2818400	1150500	1069900	674670	989880	789550	1686000	2E+06	1072800	1693900
MEDP606	2-(3,4-dimeth	Benzene and su	8467500	1E+07	8E+06	8E+06	9708100	11275000	8428900	8227000	5306000	8573200	8E+06	7E+06	9E+06	8E+06	8251700	1.1E+07	11380000	17773000	8199700	6949000	6614500	8733000	6433300	9618000	8E+06	7817300	9166800
MEDP611	N-Acetyl-L-H	Organic Acid A	3079875	3E+06	4E+06	3E+06	2400600	3893300	3646000	2991200	1985200	2279000	5E+06	3E+06	2E+06	3E+06	2657500	2866500	2677500	7798100	2643800	5367300	3878600	3008300	6664500	3329800	4E+06	2152000	4016700
MEDP614	N-Methyl-a-a	Organic Acid A	19800375	2E+07	2E+07	2E+07	22382000	19461000	1.6E+07	2.2E+07	2.1E+07	2E+07	2E+07	2E+07	3E+07	2E+07	1.9E+07	2.3E+07	23483000	16593000	19597000	20210000	18745000	19032000	18435000	18096000	2E+07	22951000	19058000
MEDP616	N-Acetyl-D-p	Amino Acid m	201246.3	658248	254184	175490	210800	131320	267740	170400	123640	251730	278850	1E+06	241080	422410	113940	416130	412070	2252400	243950	105340	211850	226780	98674	549310	336680	100030	404810
MEDP617	2-Furoylglycin	Amino Acid m	115460.8	216664	9	9	9	426710	29683	304250	9	42576	120440	9	9	157730	9	310440	9	1265100	9	9	9	9	9	9	9	9	9
MEDP618	2-Methylbuty	Lipids_Fatty Aci	4570788	4E+06	4E+06	5E+06	5655100	4436400	2494000	4296100	4106900	7706800	3E+06	4E+06	5E+06	5E+06	4128400	4973000	5030600	1672900	3778400	2922200	4572500	4057600	5406300	6652700	3E+06	4108600	2471200
MEDP627	Isonicotinic ac	Organic Acid A	20918000	2E+07	2E+07	3E+07	29724000	15976000	1.4E+07	1.7E+07	2E+07	2.7E+07	1E+07	1E+07	2E+07	2E+07	7249800	1.5E+07	19880000	8864000	1.80E+07	21049000	14925000	13073000	15397000	19334000	1E+07	11486000	15098000
MEDP631	Dimethyl fum	Lipids_Fatty Aci	24312625	3E+07	3E+07	3E+07	37321000	13942000	2.3E+07	2.3E+07	2.4E+07	3E+07	2E+07	3E+07	5E+07	4E+07	1.5E+07	3.7E+07	34419000	28606000	32059000	29252000	34277000	32870000	33160000	26182000	2E+07	24799000	17882000
MEDP632	beta-Cubeber	Lipids_Fatty Aci	145034.8	199215	216615	79115	147570	170060	93373	247880	126150	157200	138930	270340	174510	249310	130200	226880	235370	156450	150660	154680	251670	481230	225220	139070	145000	170550	165500
MEDP636	Furfural	Organic Acid A	187877.5	179740	174878	138610	177550	134390	228670	181400	186660	157590	298150	195430	154940	201730	157440	135040	244930	218840	129570	181490	200580	100350	137520	155780	230700	176880	215720

MEDP637	L-phenylalany	Organic Acid A	1107101	1E+06	1E+06	1E+06	1515200	1052500	837540	1114400	897760	1375700	834410	1E+06	2E+06	2E+06	1011600	1310300	1901600	1089100	1493200	1642000	1197600	1258900	1143600	1555300	1E+06	958230	1090300
MEDP638	LysoPE(16:1(9.	Lipids_Fatty Aci	770683.8	754368	560879	579410	366990	583960	608890	1066000	1903100	603990	453130	1E+06	766330	622330	961550	817580	706150	655020	423780	699560	281430	495920	371310	612990	650640	388970	986210
MEDP642	3-Hydroxy-DI	Amino Acid m	221853.8	237928	249861	242890	228120	243400	186100	247340	232510	260890	133580	176640	262010	286890	175080	282320	243670	154360	322450	282710	294380	311070	190660	222200	284030	241940	171900
MEDP656	Isoamyl butyra	Fatty acyls	1.05E+08	1E+08	1E+08	1E+08	1.07E+08	117240000	7.5E+07	1E+08	1.1E+08	1.2E+08	1E+08	1E+08	1E+08	1E+08	8.7E+07	1.1E+08	110880000	97288000	122430000	1.16E+08	114750000	124720000	1.15E+08	101940000	8E+07	100180000	84295000
MEDP657	EthylHeptanoa	Fatty acyls	98723750	1E+08	1E+08	9E+07	1E+08	103080000	7.2E+07	9.6E+07	9.8E+07	1.2E+08	1E+08	1E+08	1E+08	1E+08	8.4E+07	1.1E+08	101760000	87345000	117480000	1.04E+08	101290000	112890000	1.07E+08	93962000	8E+07	93873000	80370000
MEDP658	Ethyl dodecan	Fatty acyls	242738.8	255311	260339	241120	213240	225790	296630	294860	210850	227740	231680	269360	299920	242080	254700	225260	252180	276710	222280	275490	287410	228840	267310	265420	223870	315950	218420
MEDP659	Propylpropion	Fatty acyls	17968625	1E+07	1E+07	2E+07	23486000	20275000	1.2E+07	1E+07	2.4E+07	1.9E+07	2E+07	1E+07	1E+07	1E+07	9554200	1.5E+07	10469000	8845200	13646000	16065000	26372000	10966000	19077000	11323000	9E+06	10965000	8594900
MEDP660	1,4-Xylene	Benzene and su	120422.5	120463	133233	126240	147360	120600	112270	95163	124220	93407	144120	108410	104760	111940	139480	154160	77611	120570	146770	113820	133080	221360	142280	90572	121220	141500	102030
MEDP661	Parachlorophe	Phenols And Its	1253613	2E+06	2E+06	1E+06	1248200	1265500	1100800	1150900	1251200	1202000	2E+06	1E+06	2E+06	2E+06	1373700	1829200	1683900	1522900	1674100	1262800	1893700	3569000	2076200	1722000	2E+06	2022700	2228300
MEDP662	1,2-Dichloroe	Hydrocarbon d	3714838	3E+06	3E+06	4E+06	3896200	4150200	4020800	3592200	3148800	3207500	4E+06	4E+06	3E+06	3E+06	3047000	3469600	2998100	2469200	2578100	3500000	3508300	2988200	3333700	3376600	3E+06	2448900	3012400
MEDP664	L- tyrosine me	Amino Acid m	97320.75	128464	123377	108390	85344	93085	51928	130040	105990	133690	70099	140200	137990	134580	68465	118970	175510	101540	150460	85596	94567	360870	136570	99879	57853	82451	69233
MEDP666	Imidodicarbor	Others	1554010	1E+06	1E+06	1E+06	1676400	965430	974890	2389600	780560	1442500	3E+06	1E+06	1E+06	2E+06	1012900	1875000	1227300	1030000	1851800	1370700	1195700	1135500	1023600	1311300	892570	1020000	945360
MEDP667	m-Xylene	Benzene and su	129877.8	122398	139604	140170	119610	98932	125790	104670	146730	166800	136320	133300	151340	150050	130510	121100	74563	108990	109330	132060	146720	236570	166150	102670	113770	125580	93309
MEDP668	m-Cresol	Phenols And Its	1897988	2E+06	2E+06	2E+06	1857900	1398200	2330300	1464200	2280400	2116300	2E+06	2E+06	2E+06	2E+06	2025100	1521100	2291900	2131100	2086700	2125400	2016600	1099500	1860500	1901100	2E+06	2095800	1383200
MEDP670	2,6-Dimethylp	Pyridine And Py	64901.38	63618	51374	73124	76093	46545	54495	69655	59862	76427	63010	88743	110390	63284	29693	72707	46654	40928	56541	53989	49640	60081	52713	33197	49540	53179	58651
MEDP671	2,6-Dimethylp	Heterocyclic co	1471690	2E+06	1E+06	2E+06	1730000	992020	1518500	1389500	1561500	1801100	1E+06	2E+06	2E+06	2E+06	1846500	1258200	1597400	1662800	1679800	1557700	1624000	1068600	1575200	1663400	1E+06	1715500	1154300
MEDP673	Cyclohexanon	Ketones	1.5E+08	1E+08	1E+08	2E+08	1.59E+08	158020000	1.1E+08	1.5E+08	1.5E+08	1.8E+08	1E+08	2E+08	2E+08	2E+08	1.2E+08	1.5E+08	154370000	1.27E+08	164120000	1.55E+08	156910000	138720000	1.6E+08	138450000	1E+08	127300000	1.05E+08
MEDP674	3-Methylpyric	Pyridine And Py	421357.5	473658	304954	434470	603400	267320	411270	350840	399080	375160	529320	563200	779990	433900	312510	381140	238170	753060	327290	266460	166720	499960	206010	383160	321940	261140	334240
MEDP675	2-Picoline	Pyridine And Py	349927.5	392135	299524	305280	543350	251780	353020	301230	236260	312300	496200	433130	562010	357520	256950	292850	240690	622590	371340	275680	250990	498120	160070	362750	325780	213490	309310
MEDP676	Butyl 3-methy	Fatty acyls	67097375	7E+07	7E+07	7E+07	63979000	73091000	5.3E+07	6.1E+07	7.1E+07	8E+07	7E+07	8E+07	8E+07	7E+07	5.1E+07	7.2E+07	72410000	55477000	78243000	72807000	72862000	79375000	75955000	66401000	6E+07	68291000	56390000
MEDP677	Butylamine	Amines	316633.8	337808	439165	354310	424620	389340	247800	376720	132840	301510	305930	396210	343730	472920	243400	221840	271080	497090	256190	462530	675710	330690	548690	377190	#####	419470	399040
MEDP681	2,2,2-Trichlorc	Alcohol	1670850	2E+06	3E+06	2E+06	1433700	1565900	1438800	1697400	1717000	2035600	2E+06	2E+06	2E+06	2E+06	2260600	1572500	2387900	2067700	2223100	1690000	1825500	5465200	2593300	1894400	2E+06	2027900	2522800
MEDP682	Hydroxyaceto	Ketones	143171.6	149804	129575	132700	87319	219790	176990	103740	181710	84714	158410	130500	96673	443480	86358	124610	89795	137400	89614	221490	70220	133780	142830	181410	64453	150530	71885
MEDP684	Octisalate	Organic Acid A	788213.8	804283	817529	794970	748980	833480	776920	729120	870050	799010	753180	843610	793340	798360	816880	708950	744820	871960	856340	820130	790470	817030	879610	826880	792110	726040	887960
MEDP685	Methylcysteine	Amino Acid m	5216875	6E+06	1E+07	5E+06	5015900	6894400	5183100	6400300	3801700	4658700	5E+06	4E+06	5E+06	5E+06	5483500	7128100	6751900	9596800	4121700	25485000	6764600	10659000	21148000	7623700	6E+06	3784400	5769700
MEDP686	2-Acetylfuran	Heterocyclic co	19948250	2E+07	2E+07	2E+07	23572000	14144000	1.9E+07	######	1.8E+07	2.7E+07	2E+07	2E+07	2E+07	2E+07	2.7E+07	1.6E+07	26129000	28634000	27704000	27345000	25721000	21658000	27149000	28688000	2E+07	27551000	18710000
MEDP687	2-Acetyl-5-m	Heterocyclic co	152742.5	150993	133794	181290	160410	154800	179450	116440	171080	118340	140130	157740	159380	170530	108890	187390	157560	126130	140320	217310	66325	144230	48724	206810	135360	110130	141460
MEDP689	TranexamicAc	Organic Acid A	134118	119275	117889	135490	208200	156090	76020	114400	133650	196750	52344	176500	133870	97229	102740	77728	119130	62259	184740	101600	104560	171310	156100	133790	86876	107940	80935
MEDP692	Triethylamine	Hydrocarbon d	377503.8	388025	555073	387500	292630	433150	407260	380140	376120	295660	447570	349860	250900	314500	621830	318360	396690	441650	410410	450090	293530	736720	279450	575920	668630	612520	823720
MEDP697	2,6-Di-tert-bu	Benzene and su	514421.3	624000	576585	605120	547900	297010	336970	282390	434920	1097400	513660	375930	677500	907640	943170	713480	164210	457010	753060	540940	787710	317430	426480	732390	550790	596280	660660
MEDP699	D-(+)-Galactc	Carbohydrate r	242807.5	282903	268288	257760	218780	237930	228080	312360	190440	276460	220650	227940	430400	422700	303930	256230	173450	244140	204430	225030	246830	259930	368300	233070	260580	301060	251500
MEDP700	Methylbeta-D	Carbohydrate r	637947.5	580265	764610	506930	641780	674420	530130	842040	658250	736350	513680	632400	638010	712840	517310	491240	739860	309710	600750	970040	834260	1055100	839300	514990	707000	621950	574240
MEDP703	Atrazine	Heterocyclic co	191416.3	184161	199768	181290	198120	185350	169940	243100	168750	193890	190890	229700	154510	172020	159030	149660	241140	193920	173310	204170	218540	153950	212430	197040	179200	219500	213310
MEDP704	N-Methylacet	Amines	3273450	3E+06	4E+06	3E+06	2689200	3384600	3512700	3204000	3600100	2708000	4E+06	3E+06	2E+06	3E+06	4878100	2885100	3146700	3746000	3653800	3794100	2875600	4983300	3249700	4716300	5E+06	4455500	5043500
MEDP706	9,12-Octadeca	Lipids	81790.25	73308	98995	51149	54926	85612	98656	78731	97245	74863	113140	70289	60969	87534	80686	83608	68916	73020	61438	128190	54616	103880	60213	94282	115460	87236	148080
MEDP707	2-Pentadecan	Ketones	364986.3	416126	339369	367790	348150	406160	419130	376040	298030	366210	338380	385650	787910	443910	397470	352850	257050	411710	292460	303970	308910	254040	411900	332020	341470	403970	358670
MEDP709	Methyl dihydr	Organic Acid A	517161.3	641846	529753	516770	495270	617750	564960	497060	390670	494580	560230	557490	1E+06	772540	640470	468750	391490	584950	453380	513910	537980	406190	662330	474970	566620	543960	532060
MEDP715	2-Methylfurar	Heterocyclic co	118935.1	131198	96102	123190	139950	110010	117970	111560	152330	109110	87361	136640	171460	145690	91573	172970	112710	97180	121360	136550	66446	135570	82575	109260	78949	66763	92701
MEDP716	cis-Citral	Aldehyde	114264.3	70982	67696	224180	83926	40440	26554	46531	200790	268070	23623	95761	90936	80523	52250	77440	53652	20216	97074	65632	44267	34000	74610	51693	55479	198740	17150
MEDP717	2-Heptanol	Alcohol	15666375	2E+07	2E+07	2E+07	12038000	16570000	1.2E+07	1.3E+07	2.5E+07	1.8E+07	1E+07	2E+07	3E+07	6E+07	8406500	1.6E+07	22235000	12550000	25377000	18118000	34304000	12823000	15327000	12978000	2E+07	13905000	8732500
MEDP718	Methylisobuty	Organic Acid A	80957.75	89679	175981	82755	30287	123940	93541	71666	85503	59730	100240	61500	44322	64996	172850	84681	93575	103600	91909	108900	129930	232110	111560	145330	233410	221510	225100
MEDP719	N-Nitrosodiet	Polyamine	629105	908545	2E+06	526090	762380	762430	712040	504600	842060	519340	403900	899920	620670	1E+06	722480	1142000	1236500	1025700	588390	925430	1225700	2548600	1468900	1250200	2E+06	1993100	2147100
MEDP724	3,5-Dinitrosali	Organic Acid A	287445	318733	327149	346590	238630	243370	283720	391340	282990	315830	197090	279650	303080	370150	352480	287120	370140	263610	323630	316140	293190	328030	383530	346530	262340	342370	345060
MEDP725	DL-Norleucine	Amino Acid m	70579000	9E+07	6E+07	6E+07	64994000	87703000	6.7E+07	7.5E+07	5.3E+07	7.1E+07	8E+07	7E+07	6E+07	9E+07	6.5E+07	8E+07	118750000	1.64E+08	42140000	60977000	39557000	57552000	44444000	85549000	9E+07	45071000	94809000
MEDP726	3-Methylthiop	Heterocyclic co	1.73E+08	2E+08	2E+08	2E+08	1.88E+08	181670000	1.3E+08	1.7E+08	1.8E+08	2E+08	2E+08	2E+08	2E+08	2E+08	1.4E+08	1.8E+08	179750000	1.47E+08	192980000	1.78E+08	169200000	162820000	1.9E+08	159660000	1E+08	147740000	1.26E+08
MEDP729	Barbituric acid	Heterocyclic co	8997550	1E+07	2E+07	9E+06	10195000	7907000	8957300	1.1E+07	1E+07	6341200	8E+06	1E+07	1E+07	1E+07	9328100	1.4E+07	16940000	11917000	12885000	11842000	15101000	21953000	19812000	19371000	2E+07	22381000	23739000
MEDP732	2,4-Dichlorop	Benzene and su	355221.3	399593	424520	301640	382280	358650	347560	399940	309540	434470	307690	376110	394830	436390	414050	367280	347140	459780	401160	436270	516960	370080	444280	320270	367190	464040	477070
MEDP733	o-Xylene	Benzene and su	98724	120118	125356	107170	130630	76337	92845	99684	93605	96903	92618	125800	134020	93385	133660	99991	106450	141840	125800	128280	130620	226630	97597	100960	98458	116100	104200
MEDP734	2-Aminophen	Phenols And Its	73266.88	79920	78583	70500	77463	99035	71597	58361	63652	61022	84505	67430	87384	70192	56315	100500	29288	120980	107270	110970	64785	68058	63807	96268	71954	57116	95702
MEDP735	methyl α-D-g	Carbohydrate r	870875	695423	947885	829020	1105400	847340	834980	773130	986370	1009400	581360	662620	899360	848330	567360	615140	775590	377650	817330	926350	1010400	1074500	1050900	674560	715470	1066000	1064900
MEDP741	2,4,6-Trimethy	Phenols And Its	1700	2015.9	3052.8	1841.9	9	3120.3	2318.2	9	1900.5	2119.3	2281.8	2700.2	9	3129.3	2298.7	2251.9	1297.9	1853.9	2586.3	4090.5	2425.5	2937.8	2187.3	3292.1	3060.4	3719.4	2709.6
MEDP747	alpha-Terpine	Terpenoid	28211.5	30589	32617	24584	29167	32355	21366	26302	24262	34673	32983	35877	30065	33857	25413	22203	29571	32744	34985	29302	24228	45657	31756	26293	41058	30085	32555
MEDP749	ChloralHydrate	Aldehyde	2.95E+08	3E+08	3E+08	3E+08	3.89E+08	220650000	2.9E+08	######	2.9E+08	3.5E+08	2E+08	3E+08	4E+08	4E+08	2.1E+08	3.5E+08	342440000	3.09E+08	343030000	3.2E+08	325200000	297200000	3.34E+08	313330000	3E+08	261880000	2.26E+08
MEDP751	3-(Methylthio)	Aldehyde	544982.5	528921	583286	496000	585020	616860	488470	584580	418230	617810	552890	546780	535350	694450	402040	571900	479840	513280	487730	693420	415170	519970	626620	601430	627860	496010	685810
MEDP752	DL-Leucine	Amino Acid m	79371000	9E+07	7E+07	8E+07	71527000	96254000	9.1E+07	8.4E+07	5.6E+07	7E+07	9E+07	8E+07	7E+07	1E+08	6.5E+07	8.6E+07	134010000	1.64E+08	45430000	69301000	42252000	62460000	51204000	98078000	1E+08	49246000	97384000
MEDP759	Glycyrrhetinic	Terpenoid	1054110	1E+06	1E+06	888360	995990	875990	832640	1283900	995100	1182000	1E+06	2E+06	1E+06	1E+06	1292900	1151700	1561300	1547400	917910	741590	770970	1364000	1136500	1166200	1E+06	1197300	1267500
MEDP765	3-(Methylthio)	Alcohol	128241.4	125254	156513	109700	132300	113900	117190	85801	132410	166570	168060	123100	148420	111040	153930	104010	95504	149180	116850	149830	154230	302060	136830	120780	93092	149090	146190
MEDP767	Aspirin	Organic Acid A	1.68E+08	2E+08	2E+08	2E+08	1.85E+08	182420000	1.2E+08	1.5E+08	1.7E+08	2E+08	2E+08	2E+08	2E+08	2E+08	1.3E+08	1.7E+08	171400000	1.39E+08	184670000	1.68E+08	177090000	145870000	1.76E+08	154590000	1E+08	144730000	1.21E+08
MEDP770	2-Aminoethyl	Benzene and su	168325	197388	173629	160760	144470	186170	198200	166610	149930	173800	166660	176860	354290	254140	198760	169190	119080	190450	116330	167740	168520	123280	208580	154620	161590	228560	176140
MEDP772	p-Menth-1-e	Terpenoid	81940	99898	81988	66806	101840	80650	93324	59688	74298	109620	69294	84638	71004	123280	108720	124200	111780	71933	103630	97582	76151	100010	63054	82364	81582	68893	86267
MEDP776	gamma-Nona	Lactone	9	5760.8	9	9	9	9	9	9	9	9	9	9	9	9	9	9	9	23763	22269	9	9	9	9	9	9	9	9
MEDP778	Ethyl butyrate	Fatty acyls	4170850	4E+06	5E+06	4E+06	3436900	3020900	2196700	2702200	4616100	7427700	6E+06	5E+06	4E+06	5E+06	2493100	4365800	3961900	2938300	6630400	3588200	4553800	6635800	7545600	4006300	2E+06	5084600	3927000
MEDP779	1-Heptanol	Alcohol	11791600	1E+07	1E+07	1E+07	10218000	10054000	8601500	1E+07	1.5E+07	1.9E+07	1E+07	2E+07	9E+06	1E+07	7675000	1.2E+07	13958000	6819600	10084000	12895000	17851000	8369900	10670000	12010000	6E+06	9973000	13231000
MEDP781	2-Undecanon	Ketones	5795663	6E+06	5E+06	7E+06	5232600	6840000	6571100	3993700	7649100	4356200	5E+06	6E+06	7E+06	6E+06	3989500	7520400	5975200	5993200	4964000	8900700	2616700	5691800	2322700	7715300	5E+06	3723300	5695800
MEDP782	1-DECANOL	Alcohol	37679625	4E+07	4E+07	4E+07	38141000	41097000	2.9E+07	3.6E+07	3.8E+07	4.3E+07	4E+07	4E+07	4E+07	4E+07	3.3E+07	3.8E+07	40995000	34652000	46750000	38733000	40596000	44049000	43050000	37596000	3E+07	37373000	31441000
MEDP783	10-UNDECEN	Fatty acyls	9303613	1E+07	1E+07	1E+07	13324000	6046200	9100500	9381800	8924000	1.2E+07	6E+06	1E+07	2E+07	1E+07	5803400	1.2E+07	13116000	10472000	12229000	10539000	12120000	10981000	11198000	10754000	9E+06	8927800	7144200
MEDP784	Undecanal	Aldehyde	3788850	4E+06	3E+06	5E+06	3416900	4514100	4260000	2640400	4976400	2873800	3E+06	4E+06	4E+06	4E+06	2612400	4992700	3849000	3813300	3286300	5784100	1703300	3718200	1587900	5150700	3E+06	2394700	3744300
MEDP785	2,6,6-Trimethy	Ketones	53355.5	63377	75711	65898	74694	65442	52996	50239	9	49721	67845	73597	9	62226	55059	52110	90803	102090	71125	44682	97702	91001	81287	76658	72313	71803	70239
MEDP789	2-Pentyl-3-pl	Aldehyde	100104.8	108977	115279	101530	98481	108520	97930	96099	95204	106700	96374	101600	97783	119600	126500	110870	88099	108970	118390	113940	151560	104330	100660	109830	105300	109580	127030
MEDP792	PYRROLIDINE	Heterocyclic co	259125	338099	286971	285120	259140	270330	295190	234910	172310	234510	321490	310280	194000	216670	229580	334440	488510	641820	289490	227500	199750	196670	271850	391430	349830	266970	391770
MEDP794	Octanal	Aldehyde	558101.3	732085	1E+06	545930	574590	520270	550160	582240	614740	618710	458170	612190	786110	688660	716730	818310	734600	625690	874390	891490	799100	1523800	902870	1041800	1E+06	1154200	1105500
MEDP796	Pyrene	Benzene and su	104440.9	110740	127941	101230	113740	99336	84491	109460	111620	106390	109260	90890	113640	108630	117880	121590	89743	122810	120740	144920	175670	94779	111680	105970	102660	130010	157840
MEDP799	(-)-Menthone	Ketones	7501875	7E+06	8E+06	8E+06	7160400	7055100	8381500	7856000	7557100	6955900	7E+06	7E+06	8E+06	7E+06	7231900	7611400	6625200	7165200	7778200	7307300	5656200	7577000	6574100	8495000	9E+06	7546400	8203900
MEDP800	MESITYL OXID	Ketones	1.5E+08	1E+08	1E+08	2E+08	1.65E+08	162630000	1.1E+08	1.4E+08	1.6E+08	1.7E+08	1E+08	2E+08	2E+08	2E+08	1.2E+08	1.5E+08	158540000	1.29E+08	166340000	1.55E+08	149470000	133050000	1.62E+08	136590000	1E+08	129300000	1.07E+08
MEDP804	Dimethyl azela	Fatty acyls	7951138	8E+06	9E+06	6E+06	8055100	9369400	6712500	8964800	7268200	9159100	8E+06	5E+06	7E+06	9E+06	8844100	7600000	5888200	5921300	10872000	10055000	8289800	8536900	7261900	7866400	9E+06	6641500	11778000

MEDP805	DL-2-Aminob	Organic Acid A	12864625	2E+07	1E+07	1E+07	10492000	16335000	1.1E+07	1.9E+07	1.1E+07	1.1E+07	1E+07	1E+07	1E+07	3E+07	1.4E+07	1.9E+07	13860000	11984000	11882000	11002000	12547000	16671000	17806000	17410000	1E+07	17341000	13163000
MEDP806	Monopalmitin	Lipids	914395	906954	888731	964790	907400	931570	963840	868470	927350	844680	907060	843600	930750	942120	972710	894560	765660	933410	972820	836120	1000800	777180	980660	907120	830380	863240	914350
MEDP808	Sulfaguanidine	Benzene and su	917677.5	952438	953363	847720	831780	932970	680070	1109600	1056800	969210	913270	1E+06	915370	1E+06	872300	970120	974850	772220	880440	1086400	839770	899980	1136600	802120	1E+06	946020	884910
MEDP810	Linalyl oxide	Alcohol	171227.5	169255	148869	205040	161080	205860	200440	106370	223800	130420	136810	164120	188500	170760	122340	212360	183740	166070	146150	251600	82935	166030	71906	193850	140250	123000	161380
MEDP812	5-METHYLFUI	Aldehyde	541585	549996	564081	602440	568410	630490	381000	409980	550430	675750	514180	618960	487790	609820	411140	576180	552460	489170	654450	565920	641970	636760	650140	533860	402160	604420	477420
MEDP813	3-Nitro-L-tyro	Amino Acid m	2111200	2E+06	3E+06	2E+06	2691700	2148700	1797000	2019000	2193200	2512600	1E+06	2E+06	3E+06	3E+06	1833300	1900900	1845700	1822000	2735900	4281900	3615800	2883700	3833300	2223300	2E+06	2012900	1719900
MEDP814	METHYL VALE	Fatty acyls	2858738	3E+06	2E+06	2E+06	2077900	2240600	5164000	1837300	1812400	5277700	2E+06	5E+06	5E+06	2E+06	2142400	1692100	2954600	2826400	1823000	2326900	1514500	1521800	3113900	1878700	1E+06	1461900	1620600
MEDP817	PROPYL HEXA	Fatty acyls	11206663	1E+07	1E+07	1E+07	10561000	13338000	8281300	1.1E+07	1.1E+07	1.3E+07	1E+07	1E+07	1E+07	1E+07	9363000	1.1E+07	11890000	9620200	13545000	11703000	11651000	13560000	13639000	10478000	9E+06	10854000	9244500
MEDP818	1-PENTADEC	Alcohol	304473.8	312725	320395	342920	293630	304970	302100	356000	201970	297820	336380	318840	234590	382630	308440	332150	394300	267170	263680	315750	304000	318770	340450	285250	302310	319100	377530
MEDP820	Gamma-Capr	Lactone	80272.25	76852	80791	58822	160760	56428	54931	66623	74524	100730	69360	69664	95559	102720	59841	111310	55483	38129	82106	61001	75820	64880	161800	49636	88207	73952	71033
MEDP821	6-Methylnicot	Pyridine And Py	42513000	5E+07	2E+07	5E+07	66829000	33115000	2.6E+07	3.7E+07	4.6E+07	6.3E+07	2E+07	4E+07	6E+07	6E+07	3.3E+07	5.7E+07	29700000	19141000	68312000	9	8871600	32983000	1627200	12421000	1E+07	36634000	17434000
MEDP822	1-butanol	Alcohol	74234.63	96189	92324	87388	61179	126940	48326	9	116720	48795	104520	146240	9	74172	81084	105310	216080	55995	90624	92527	97284	186110	54274	94575	48016	64397	101410
MEDP824	Trimethoprim	Benzene and su	34148.13	31977	34073	53869	83029	25599	16517	26160	18931	49071	9	40484	30659	31265	28483	36639	35955	9	52320	26669	42585	59176	46550	32676	19929	20141	24855
MEDP826	Vitamin E acet	Co-Enzyme Fac	1501625	2E+06	2E+06	1E+06	1714400	1594000	1485600	1448100	1397800	1456800	2E+06	######	2E+06	2E+06	1764200	1576500	1623100	1388900	1713700	1493200	1893000	2341300	2249600	1944700	2E+06	1837400	2069800
MEDP828	2-Methyl-1-p	Alcohol	78493.88	64636	63288	44113	84771	110080	64478	36191	76024	43244	169050	161740	27550	25020	79479	56026	63182	52455	51632	44957	34271	111240	69010	49853	76685	52268	68016
MEDP829	1,2-DIAMINO	Polyamine	10734.21	12068	13258	13301	14533	14973	7375.7	9	6007.7	23089	6585.3	9559.2	5358.4	14375	7145.1	12817	6980.2	23633	16676	8359	17256	11459	9368.4	17477	12381	12602	17161
MEDP830	2-Butanol	Alcohol	117772.5	82003	99100	100580	92109	111970	150220	52672	105460	72379	256790	90131	64496	51273	130590	80145	114650	56320	68420	77172	170590	85170	124660	83151	64340	116800	70919
MEDP831	1-Aminoprop	Alcohol	2467278	3E+06	122695	150740	91044	12130000	421510	4293800	99750	891380	2E+06	97887	95520	3E+06	220200	4484800	225210	12413000	223630	256840	124850	131750	132110	149130	44798	56929	85151
MEDP833	Sulfamethoxy	Benzene and su	1623775	2E+06	2E+06	1E+06	1625700	1391800	1555900	1730100	1699900	1801100	2E+06	2E+06	2E+06	2E+06	1884500	1887500	2006100	2012200	1961100	1683200	2054100	2682900	2244700	2200200	2E+06	2473900	2393600
MEDP834	3-Methylsalicy	Organic Acid A	160850	141359	161068	143130	183630	168450	168410	170970	123140	144670	184400	136980	126280	129370	149210	178380	134690	143130	132830	141320	179140	160070	130880	148710	189460	137930	201030
MEDP835	DIISOBUTYL P	Benzene and su	8446738	9E+06	1E+07	7E+06	7650600	7416300	7726800	9795900	7797500	1E+07	1E+07	9E+06	9E+06	9E+06	1E+07	9019400	8595200	8589700	7761800	10426000	1.10E+07	21301000	10646000	8494100	1E+07	9539200	10136000
MEDP836	Dibutyl phthal	Benzene and su	4737513	5E+06	6E+06	4E+06	4397400	4328600	4663400	4653200	5392700	4781800	6E+06	5E+06	5E+06	5E+06	5055400	4773100	4901900	5138200	3519300	4662300	5163900	10970000	4842900	5570600	6E+06	5614700	5668400
MEDP839	Pulegone	Ketones	130780	120595	129466	110860	104880	133170	153040	149310	147160	115230	132590	109270	136870	132190	109320	146470	108260	113030	109350	110760	113480	147180	146170	114900	115620	119500	168120
MEDP840	Naphthalene	Benzene and su	1271938	2E+06	2E+06	1E+06	1242300	1114300	1248700	1229800	1213200	1586300	1E+06	2E+06	2E+06	1E+06	1843200	1629900	1964300	1414400	1983100	1774600	1784900	3314200	2033700	2500300	2E+06	1883000	2451800
MEDP842	1,3-Di-o-tolyl	Benzene and su	26157.5	32399	9	33520	74713	20391	9	18009	36062	26547	9	51186	88697	45293	33414	40577	9	9	9	9	9	9	9	9	9	9	9
MEDP844	FURFURYL AL	Alcohol	22489000	2E+07	2E+07	2E+07	23947000	25460000	1.6E+07	2.1E+07	2.3E+07	2.6E+07	2E+07	2E+07	2E+07	2E+07	1.8E+07	2.1E+07	22990000	18600000	24488000	22435000	21961000	20281000	24674000	20916000	2E+07	18864000	16312000
MEDP845	1-Phenylethar	Benzene and su	2.58E+08	3E+08	3E+08	3E+08	3.55E+08	187950000	2.5E+08	2.5E+08	2.5E+08	3E+08	2E+08	3E+08	4E+08	3E+08	1.8E+08	3.1E+08	319910000	2.78E+08	310130000	2.82E+08	281580000	261520000	2.97E+08	280320000	2E+08	231120000	1.96E+08
MEDP847	Cinnabarinic a	Organic Acid A	701005	614794	506028	657820	842460	821830	546760	707640	650540	872940	508050	582790	620370	876210	610700	795080	478280	336020	618900	459000	420800	458660	535800	553380	574970	555120	490490
MEDP848	D-(-)-3-phos	Others	1.33E+08	9E+07	1E+08	2E+08	69328000	181030000	1.6E+08	2.6E+07	1.6E+08	9.9E+07	2E+08	1E+08	4E+07	2E+07	2.1E+08	2.8E+07	125520000	1.94E+08	16629000	1.47E+08	33354000	133100000	94986000	132080000	2E+08	112560000	1.99E+08
MEDP849	Nα-Acetyl-L-	Amino Acid m	89236750	9E+07	9E+07	1E+08	1.2E+08	88227000	7.5E+07	7.4E+07	7.6E+07	1.2E+08	6E+07	8E+07	1E+08	#####	6.7E+07	9.8E+07	67473000	49229000	136810000	1.05E+08	109300000	79766000	1.14E+08	89742000	8E+07	82962000	59618000
MEDP850	Betaine hydro	Organic Acid A	55289750	5E+07	5E+07	6E+07	56413000	52836000	3.7E+07	8.4E+07	5.4E+07	6.1E+07	4E+07	4E+07	5E+07	8E+07	4.8E+07	5.3E+07	39185000	21866000	68975000	53612000	47620000	51249000	48350000	63129000	6E+07	48817000	61519000
MEDP851	D-Lysine mon	Amino Acid m	13228213	2E+07	2E+07	1E+07	19344000	10428000	9412100	1.4E+07	1.3E+07	2.3E+07	5E+06	6E+06	2E+07	2E+07	6001400	2.5E+07	11991000	8968100	24310000	15465000	36495000	12480000	24894000	14170000	9E+06	12461000	8002600
MEDP852	D-Histidine m	Amino Acid m	10945438	1E+07	9E+06	1E+07	19909000	9057100	8812500	6886500	1.1E+07	1.3E+07	8E+06	2E+07	3E+07	2E+07	1E+07	1E+07	7011900	7376100	8244700	10634000	10475000	6949500	10517000	8175300	8E+06	7323400	6620100
MEDP853	Choline chlori	Others	45319000	6E+07	5E+07	4E+07	31556000	57269000	4.3E+07	6.5E+07	3.9E+07	4.1E+07	4E+07	5E+07	5E+07	9E+07	5.3E+07	6.7E+07	52626000	42575000	38299000	40053000	49660000	57136000	64768000	60161000	5E+07	62976000	47671000

Table S3. The number of mice in each group for various analysis.

Experiment	Number of mice
Histopathological staining	n = 4
Laser Doppler perfusion monitoring	n = 4
Immunohistochemistry on spinal cord	n = 4
Western blotting analysis of spinal cord	n = 4
Qualitative analysis of Evan’s Blue	n = 3
Quantitative analysis of Evan’s Blue	n = 4

In total, 92 mice were used in this study, with 23 animals in each of the 4 groups. And the feces of the same batch of mice were used for metabolic profiling (sham: n = 8; SCI: n = 8; SCI+FMT: n = 8).

Table S4. The number of mice in each group for different analysis.

Experiment	Number of mice
Qualitative analysis of Evan’s Blue	n = 3
Quantitative analysis of Evan’s Blue	n = 4
Western blotting analysis of spinal cord	n = 4
Histopathological staining	n = 4

In total, 60 mice were used in this study, with 15 animals in each of the 4 groups. And the same batch of mice were used for analysis of BMS score and BMS subscore（Sham: n = 6; Sham+ALA: n = 6; SCI: n = 6; SCI+ALA: n = 6).
